# Leveraging Substrate Promiscuity of a Radical *S*-Adenosyl-L-methionine RiPP Maturase toward Intramolecular Peptide Cross-Linking Applications

**DOI:** 10.1021/acscentsci.2c00501

**Published:** 2022-08-01

**Authors:** Karsten A. S. Eastman, William M. Kincannon, Vahe Bandarian

**Affiliations:** Department of Chemistry, University of Utah, 315 South 1400 East, Salt Lake City, Utah 84112, United States

## Abstract

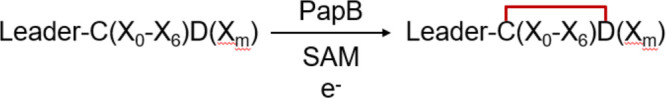

Radical *S*-adenosyl-l-methionine (RS) enzymes operate on a variety of substrates and catalyze a wide range of complex radical-mediated transformations. Radical non-α-carbon thioether peptides (ranthipeptides) are a class of ribosomally synthesized and post-translationally modified peptides (RiPPs). The RS enzyme PapB catalyzes the formation of thioether cross-links between Cys/Asp (or Cys/Glu) residues located in six Cys-X_3_-Asp/Glu motifs. In this report, using a minimal substrate that contains a single cross-link motif, we explore the substrate scope of the PapB and show that the enzyme is highly promiscuous and will accept a variety of Cys-X_*n*_-Asp sequences where *n* = 0–6. Moreover, we show that the enzyme will introduce in-line and nested thioether cross-links independently in peptide sequences that contain two motifs derived from the wild-type sequence. Additionally, the enzyme accepts peptides that contain d-amino acids at either the Cys or the Asp position. These observations are leveraged to produce a thioether cyclized analogue of the FDA-approved therapeutic agent octreotide, with a Cys-Glu cross-link replacing the disulfide that is found in the drug. These findings highlight the remarkable substrate tolerance of PapB and show the utility of RS RiPP maturases in biotechnological applications.

## Abstract 2

PapB modifies a variety of peptide substrates. The promiscuity of PapB was leveraged toward the *in vitro* synthesis of a thioether macrocyclized analogue of the FDA-approved drug octreotide.

## Introduction

Nature can access vast chemical space through enzymatic reactions to produce structurally diverse natural products that are essential *in vivo* for cellular function and survival. Recent advances in bioinformatic methods and the proliferation of publicly available bacterial genome databases have led to a substantial number of new peptide-based natural products, whose discovery is often accelerated by the clustering of biosynthetic genes in bacterial genomes.^1^ Many peptidic^2^ natural products are produced in the cell by modifications of a ribosomally synthesized and post-translationally modified polypeptide (RiPP) by one or more enzymatic steps.^3^ Unlike the nonribosomal peptide synthetases (NRPSs), where introducing sequence diversity is quite challenging,^4^ in principle, RiPPs can be readily engineered by nonsynonymous mutations in the genomic sequence of the ribosomally synthesized precursor peptide.

Posttranslational modification of amino acid residues is generally challenging using traditional synthetic chemistry methods.^5^ In most cases, notably with aliphatic carbons, the energetic barriers for substitutions that involve insertion into C–H bonds are quite high. Consequently, a growing number of RiPP maturases with this type of reactivity are garnering attention as potential tools in the arsenal of peptide chemists. Some RiPP maturases overcome the challenges by employing direct activation of the site of chemistry through radical means using enzymes in the radical *S*-adenosyl-L-methionine (SAM) superfamily.^6^

The radical SAM (RS) enzyme superfamily was first described by Sofia based on a conserved CX_3_CX_2_C motif.^7^ In the active sites of these enzymes, the Cys thiolate side chains coordinate three iron atoms of a [4Fe-4S] cubane-like cluster.^8^ The fourth Fe ion is uniquely coordinated by the amino and carboxyl groups of SAM. In the resting state, the cluster is in the +2 oxidation state. When reduced to the +1 state, the metallocenter reductively cleaves the C–S bond to the adenosyl moiety to generate methionine and 5′-deoxyadenosyl radical (dAdo·), the latter initiating catalysis by abstracting a H atom from the substrate.^8^ This superfamily has been implicated in a variety of RiPP modifications, including C–C,^9−12^ C–O,^13,14^ C–S,^15−19^ and C–H^20−22^ bond formation at unactivated carbon centers. RiPPs that are produced in pathways that involve RS enzymes vary significantly in peptide length, structure, and biological function.^1^ Most recently, even a SeCys containing RiPP that is matured by a RS enzyme has been described.^23^

PapB is a RS RiPP maturase that catalyzes the insertion of six thioether cross-links in the PapA polypeptide.^24^ PapB catalyzes the insertion of links between the Cys thiol and the β-carbon of the Asp, where the residues being linked are in a CX_3_D motif. Prior studies have shown that the enzyme can also accept Glu at the modification site and that PapB introduces the cross-link to the chemically analogous γ-carbon.^24^ In addition, PapB has also been shown to accept a shorter minimal substrate (msPapA), which only has a single pair of cross-linking amino acids in the CX_3_D motif.^24^ In this manuscript, we describe the preparation of highly active PapB that has permitted detailed *in vitro* studies to establish substrate tolerance. We show that contrary to generally accepted conventions for enzymatic transformations, PapB is promiscuous and will process a variety of peptides, including sequences that contain d-amino acids at the cross-linking site, or between Cys and Asp residues that are either directly adjacent or as far as six residues removed. We leverage these observations toward the synthesis of an analogue of the FDA-approved drug octreotide, where instead of a disulfide linkage, a C-to-E linkage is used to circularize the peptide. The implications of these observations with regard to RS RiPP maturase enzymes are discussed.

## Results

### Characterization of Purified PapB

All PapB-expressing strains were cotransformed with a plasmid containing the *suf* operon^25,26^ from *Escherichia coli*. PapB was produced as a MBP-His_6_ fusion, with a tobacco etch virus (TEV) cleavage site for removal of the tags. Cofactor-replete PapB was purified to homogeneity using His_6_ affinity chromatography for the initial separation, amylose column for orthogonal purification, followed by TEV treatment to cleave the purification tags. PapB was purified away from the tag by a second His_6_ affinity column and reconstituted by Fe/S. Gel filtration was used to remove higher molecular weight complexes that form during reconstitution (Figure S1). Since previous sequence analysis and ferrozine assays indicate that PapB likely has three [4Fe-4S] clusters, a 12-fold molar excess of iron and sulfide was added to the maturase for reconstitution. Amino acid and ICP-MS analyses of protein from multiple independent purifications show that the purified protein obtained by this procedure contains 13.5 ± 0.3 mol of iron per mol of PapB. This is consistent with three [4Fe-4S] clusters per polypeptide chain.

**Figure 1:**
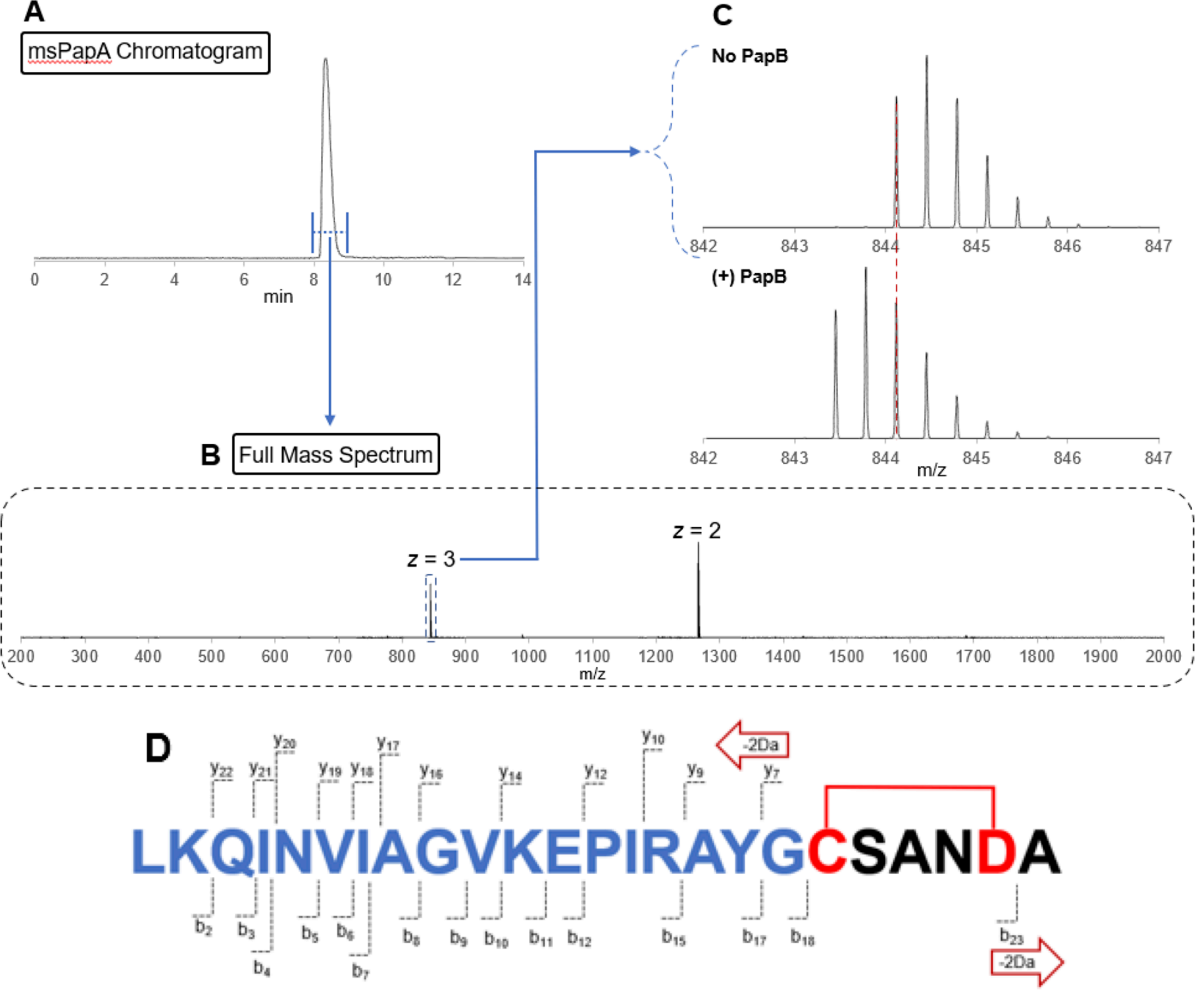
PapB introduces a single cross-link into the minimal PapA substrate (msPapA). (A) TIC of msPapA chromatographed on a C18 HPLC column. The peptide elutes at 8.1 min. (B) The mass spectrum corresponding to the peak eluting at 8.1 min is shown. The *z* = 3 charge state was chosen for most peptide mass envelope comparisons shown in this manuscript. (C) Comparison of the *z* = 3 charge state envelopes of unreacted and reacted msPapA ± PapB. A 2 Da loss upon thioether formation is apparent in the isotopic envelope after the addition of PapB. (D) Sequence of cross-linked PapB showing all of the observed *b* and *y* ions. The residues in blue represent the leader peptide sequence. The *y*-fragments after the cysteine residue highlighted in red display a loss of 2 Da upon reaction with PapB. The 2 Da loss is seen in *b*-fragments after C-terminal aspartate. This general pattern is seen in each msPapA variant processed by PapB.

The enzymatic activity of PapB was established with HPLC-purified msPapA (Figure 1). The peptide elutes at 8 min under the conditions used in the separation (Figure 1A), and HR-MS/MS reveals two clearly visible charge states (Figure 1B). Expansion of the +3-charge state (Figure 1C) reveals an isotopic envelope with the monoisotopic peak at *m/*z of 844.1201, which is <0.5 ppm of the calculated unmodified peptide (calc: *m*/*z* 844.1197). In the presence of PapB, dithionite (dT), and SAM, the monoisotopic peak of the +3 charge state shifts by 0.6716, which corresponds to a loss of 2 Da from the peptide. This is <0.8 ppm of the expected mass for a singly cross-linked peptide. We note that under these conditions, we routinely observe complete conversion of msPapA to a singly cross-linked peptide using 0.1 nmol PapB and 20 μmol msPapA in 5 min. To validate that a thioether cross-link had formed, the modification reaction was carried out in bulk, and the resulting sample was desalted and subjected to HR-MS/MS analysis. As expected for a cross-linked ranthipeptide,^24^ no fragments are seen between the C19 and D23 in the reacted peptide. Additionally, a 2 Da loss was observed in the *b*_23_ ion and in every *y* series ion above *y*_7_ (Figures 1D and S2).

We next assessed the kinetics of the modification reaction catalyzed by PapB in the presence of dT or a biological reducing system (FldA/FPR/NADPH) (Figure S3). In these experiments, the enzyme concentration was kept low (430 nM) relative to the peptide (191 μM; established by tryptophan absorbance). Under these conditions, both reduction conditions show robust turnover, with dT showing roughly three-fold faster kinetics than that observed with the biological reducing system. At 15 s, two- and four-fold increases in the concentration of PapB result in conversion of unmodified msPapA to modified msPapA that is roughly two- and four-fold greater than initial conditions, suggesting that activity is proportional to PapB concentration (Figure S4). However, increasing the msPapA concentration by two- and four-fold did not alter the distribution of the reaction, suggesting that peptide concentration was saturating (Figure S5). Therefore, the rate that is measured in these experiments is a good approximation of *k*_cat_ for PapB. Using the linear portions of the curves, we estimate turnover numbers of 7.4 ± 0.1 s^–1^ with dT and 2.6 ± 0.2 s^–1^ with the biological reducing system.

### PapB Modifies Expanded and Contracted C(X_3_)D Motifs

**Figure 2:**
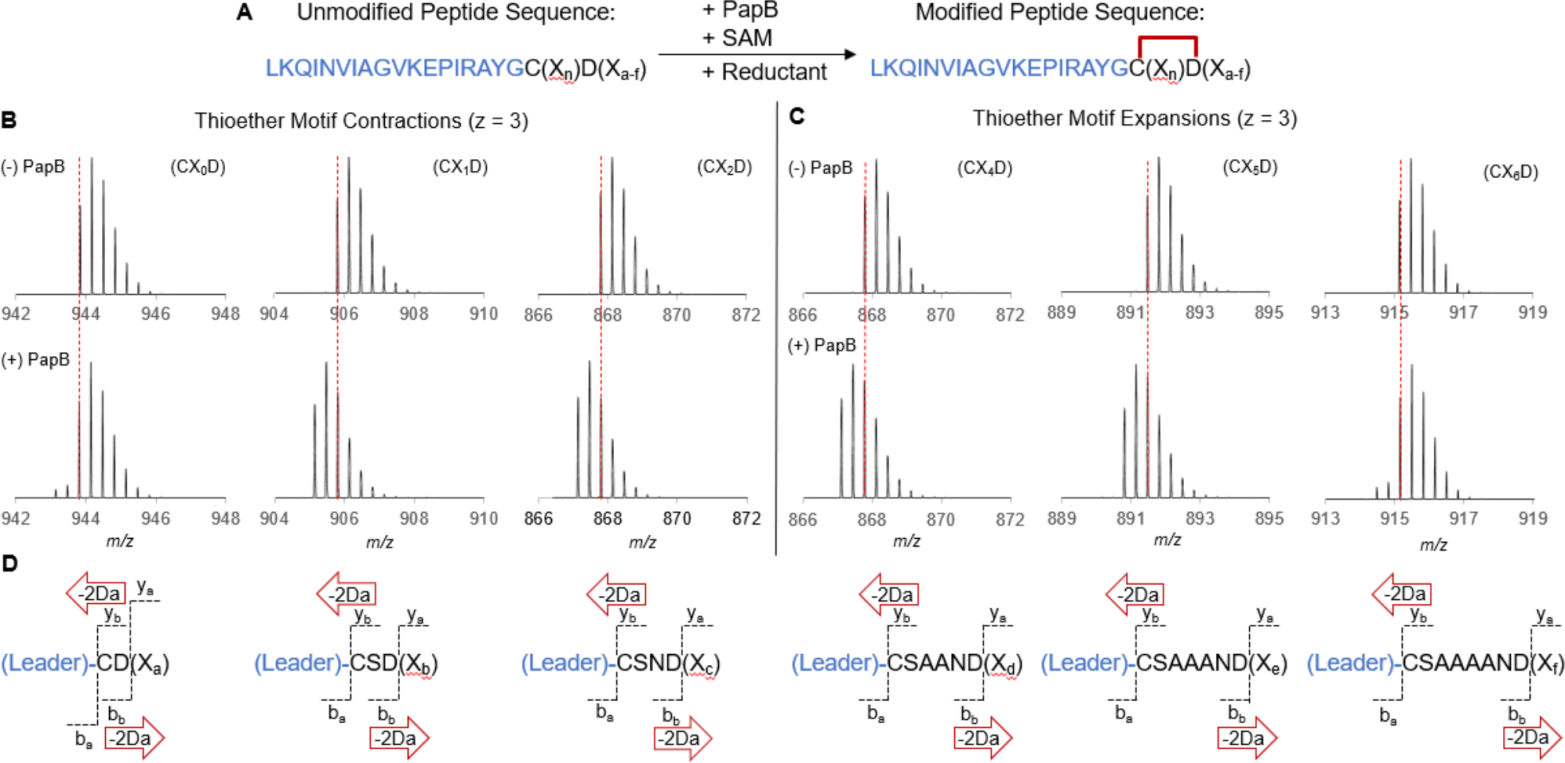
PapB recognizes and modifies expanded and contracted motifs in msPapA peptides. (A) The cross-linking reaction catalyzed by PapB requires SAM and reducing systems. The mass spectra shown in (B) and (C) highlight that PapB introduces cross-links in a PapB-dependent manner leading to a 2 Da loss. The reactions with substrates containing X = 1–5 intervening residues go to completion under these conditions. CX_0_D and CX_6_D reactions did not go to completion, but the anticipated monoisotopic masses for a 2 Da loss are still detectable. The dashed red lines represent the positions of the monoisotopic masses of the unmodified substrates. (D) The expected 2 Da loss is seen in each *b* and *y* fragment. The full tandem mass spectra, as well as tables of all observed *b* and *y* ions, are shown in Figures S13–S18. The C-terminal sequences in these peptides are as follows: X_a_ = SNNAANA, X_b_ = NNAAA, X_c_ = AAA, X_d_ = A, X_e_ = A, X_f_ = A.

To assess the sequence dependence of the modification, minimal substrates containing 0–6 amino acids between the cross-linked Cys and Asp were synthesized and incubated with PapB (Figure 2A). Remarkably, in each case, a loss of 2 Da is observed upon the addition of PapB (compare Figure 2B,C). While the reactions with 1–5 intervening residues appear to go to completion, CX_0_D (Figure 2B) and CX_6_D (Figure 2C) did not fully react—suggesting that PapB does not processes these motifs efficiently. The observed monoisotopic masses for each processed and unprocessed species of peptide agree (to <4 ppm error) with the expected monoisotopic masses (Table S6). Treatment with iodoacetic acid (IAC) suggests that no free thiols are present in the treated samples, other than the C in the unmodified portion of CX_0_D and CX_6_D (Figures S7–S12). This shows that PapB has introduced a thioether cross-link in each peptide irrespective of the length of the intervening sequence between the Cys and Asp residues.

The location of modification in each msPapA peptide variant was investigated by collision-induced dissociation (CID) MS/MS. The modified msPapA peptides were analyzed and compared to the unmodified control peptides. In each case, the samples were introduced to the mass spectrometer by direct infusion after quenching with TCA and removal of excess salts. The +3 charge state envelope was isolated and fragmented in the CID cell of the instrument. The fragmentation data showing all *b* and *y* ions that could be identified are shown in Figures S13–S18. In general, the unmodified peptides all displayed fragmentation between Cys and Asp residues. Upon modification by the addition of PapB, no fragmentation peaks are observable between those two residues. In the case of the *b* fragments, no change of mass is observed until after the Asp residue, after which a −2 Da loss is seen in each fragment. By contrast, a −2 Da loss is observed after the Cys residue in each *y* fragment.

The MS/MS data are consistent with the formation of thioether cross-links in non-α positions. Under mild CID conditions, the MS/MS spectra of sactipeptides (sulfur-to-α carbon thioether cross-linked peptides) generally exhibit fragments at each residue but show a 2 Da loss at the acceptor (non-Cys) residue due to cleavage of the thioether bridge to release the Cys to form a dehydro- residue at the acceptor site.^27,28^ By contrast, Cβ- and Cγ-thioether cross-linked peptides do not produce fragments within the macrocycle under mild CID conditions.^18^ Previous calculations of the zero-point energies of Cα- and Cβ-thioether cross-links show the Cβ-linkage to be 12 kcal/mol more stable than Cα-linkage.^18^ This stability provides a rationale for the difference in the MS/MS spectra for these classes of RiPPs. All MS/MS spectra of the msPapA thioether motif expansions and contractions demonstrate a stable macrocycle—i.e., no evidence of fragmentation between the Cys and Asp residues is observed in the data (see Figures S13–S18).

The reactions with CX_0_D and CX_6_D peptides did not go to completion; therefore, unmodified peptide fragments are also seen in these reactions, revealing cleavage between the C and D residues in the unmodified portion of the isolated envelope, serving as internal controls (Figures 2D, S13, and S18).

### PapB Tolerates Extensions from the Leader Peptide and Processes In-Line and Nested Cross-Links

**Figure 3:**
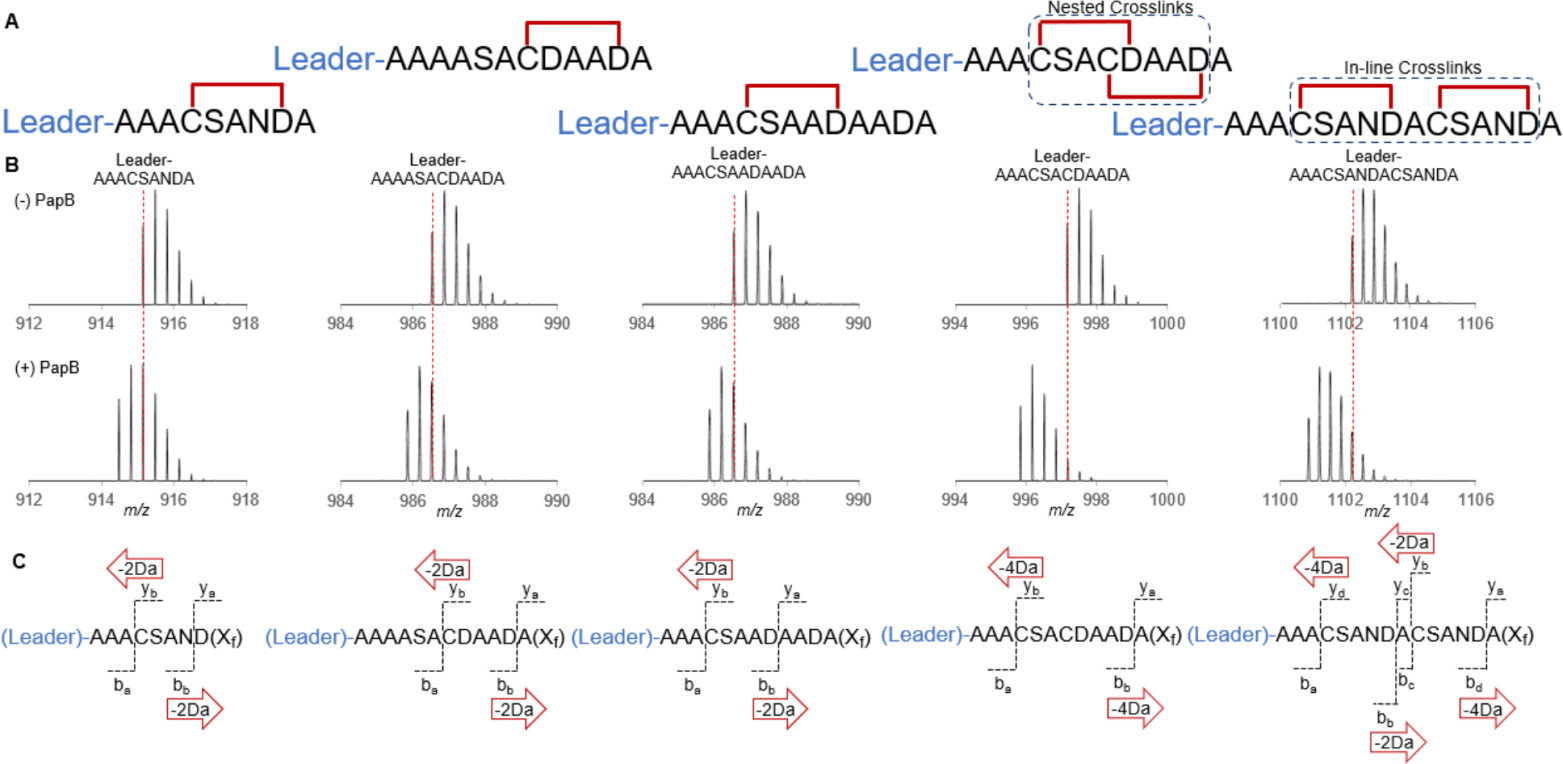
Leader extensions with single, nested, and in-line cross-links. PapB can process single CX_3_D motifs with a poly-A spacer from the leader sequence, as well as peptides that contain two motifs that are either in-line or nested. (A) The apparent cross-link locations remain consistent after distancing the thioether motifs from the leader peptide. (B) The isotopic distributions of the peptides; a shift of 2 Da in the case of single thioether motifs or 4 Da with double thioether motifs upon addition of PapB. (C) A representation of the tandem mass spectrometry results. To validate the location of the cross-links in the nested peptide, two different versions were created with a single C → A replacement. The CX_3_D thioether motif was favored over the possible CX_0_D and CX_6_D motifs in the nested variants (Figures S20–S24).

We next explored whether the sequence context of the CX_3_D sequence in the natural peptide and the specific amino acids within the motif are essential for recognition and cross-linking (Figure 3A). We did not test an exhaustive number of modifications, as the addition of three or four Ala residues immediately adjacent to the recognition motif clearly did not impair cross-linking activity. As Figure 3B shows, all the peptides that were examined were efficiently cross-linked by the enzyme. Figure 3C demonstrates that the cross-link occurs within the CX_3_D sequence, even if an alternate D residue is available downstream.

The naturally occurring PapA peptide is processed by PapB to introduce six ranthionine linkages, which are either in line with the Cys and Asp residues within a CX_3_D motif being cross-linked, or nested with the C residue occurring within one CX_3_D motif cross-linking with an Asp residue located C-terminal to it.^24^ As Figure 3 shows, we were able to cross-link both nested and in-line variants of the peptide by simply repositioning the CX_3_D element within the peptide. In the case of the in-line and nested cross-links, the treatment with PapB results in the loss of 4 Da from the peptide (Figure 3B). The observed monoisotopic masses for these species are <5 ppm of the calculated values (Figure S19). Tandem mass spectrometry reveals a similar pattern in the *b* and *y* fragments; a mass loss of 2 Da is seen in each *b* fragment after Asp and in each *y* fragment after Cys. The fragmentation data for all identifiable peaks are shown in Tables S20–S24. As with the data in Figure 2, no fragments are observed that correspond to cleavage between Cys and Asp. Finally, treatment with IAC resulted in no carboxymethylation of the modified peptide (Figures S25–S29), further supporting formation of the thioether linkage.

The results with expansions of the CX_*n*_D motif in the previous section demonstrate a lack of defined specificity in the recognition sequence, beyond the preference for Cys and Asp. The data with the nested cross-links above extend this to include distance from the leader peptide recognition sequence, as well as the individual amino acids within the processed peptide. These observations suggest that the *only* elements that guide binding and cross-linking activity is the presence of proximal Cys and Asp residues, and the leader sequence, which is presumed to be required for RiPP maturases.^24^ These observations support the notion that PapB may be able to be used widely to introduce thioether cross-links in peptides that are completely unrelated to the naturally occurring PapA substrate.

**Figure 4:**
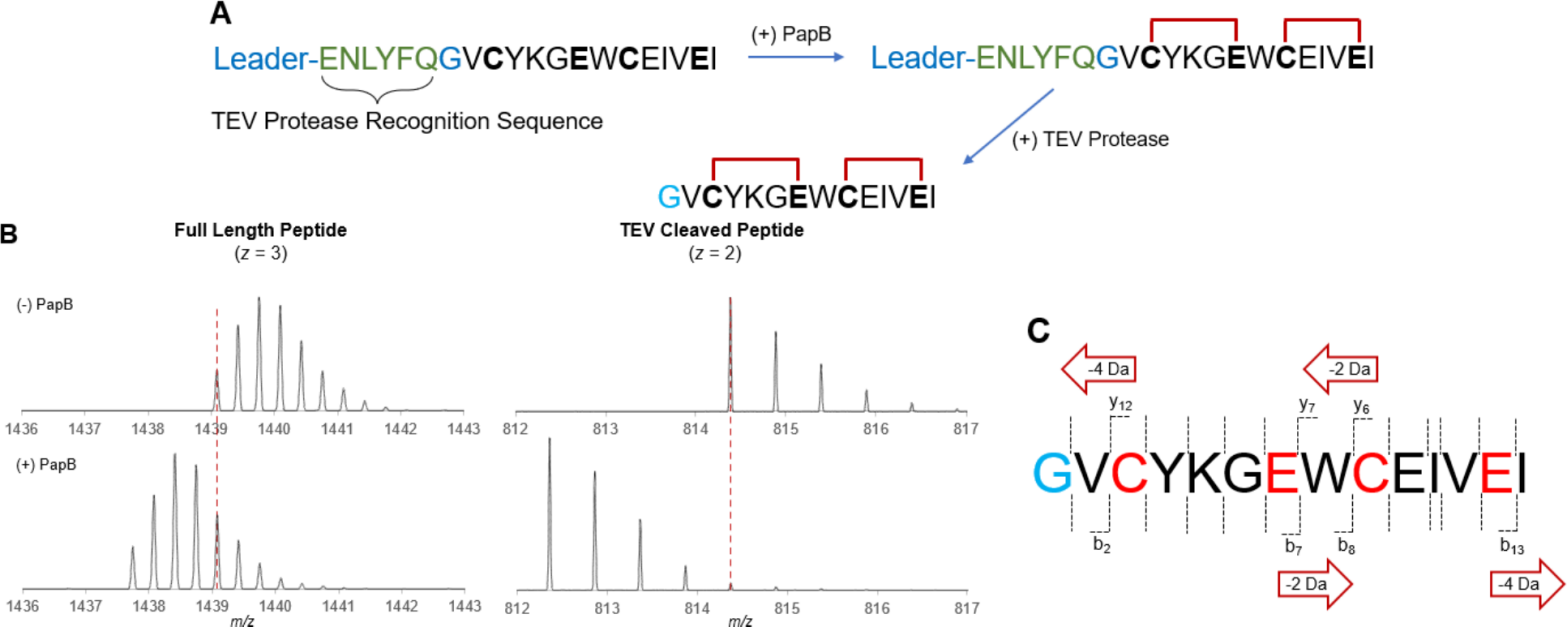
PapB produces two thioether cross-links in the AMK-1057^29^ precursor peptide *in vitro.* (A) The AMK-1057 precursor peptide contains the leader peptide sequence, a TEV protease recognition sequence, and two CX_3_E motifs. (B) Upon reaction with PapB in an *in vitro* assay, two cross-links form. Additional processing with TEV protease produces the expected dicyclized peptide. The isotopic distributions of the full-length peptide as well as the TEV-cleaved product, both unreacted and reacted, are shown. (C) Tandem mass spectrometry confirms the topology of the bonds. See Figure S31 for all observed peptide fragments.

Indeed, PapB has been used recently to prepare peptide products that are capable of binding single protein targets, such as the SARS-CoV-2 spike receptor-binding domain.^29^ The peptide in that design (AMK-1057) contained a leader sequence, which through a TEV protease recognition sequence is connected to two CX_3_E motifs. The initial report on PapB had demonstrated that both Asp and Glu are cross-linked by the enzyme.^24^ In the more recent article, however, while the peptide contained two potential cross-linking motifs, only a single cross-link was observed. Considering our *in vitro* data with the highly active protein shows that we can essentially direct the topology the modification, we revisited this result to determine if the absence of the second cross-link reflects the *in vivo* system employed rather than inherent to PapB. A synthetic peptide that is identical to the unnatural peptide reportedly used to target the SARS-CoV-2 spike receptor-binding domain^29^ was synthesized and treated with the PapB as described above (Figure 4A). PapB installs *two* cross-links in the peptide as evidenced by the loss of 4 Da in the modified peptide (Figure 4B). We next carried out TEV cleavage of the resulting product to release mature peptide, and as the MS shows, it also exhibits a 4 Da loss, localizing the modification to the peptide. The observed monoisotopic masses for both the full length and the TEV-cleaved peptide are <3 ppm of the expected values (Table S30). Tandem mass spectrometry shows a fragmentation pattern that is indicative of two thioether events occurring, one between Cys3 and Glu7, and the other between Cys9 and Glu13 (Figures 4C and S31). Therefore, the presence of a single cross-link in the reported peptide was likely due to the *in vivo* conditions employed.

### d-Amino Acids Are Processed by PapB

In initial experiments with PapA/PapB, we were intrigued by the CX_3_D spacing, which suggested that the enzyme recognizes the Cys and Asp residues as part of a helical fragment since Cys and Asp side chains would be expected to be located on the same face of an α helix. However, the ability to expand and contract the CX_3_D motif (see Figure 2) demonstrated that the three amino acid spacing of the Cys and Asp residues in the motif is not required. Indeed, the expansion and contraction results suggest that only the identity of the amino acid or specific chemical moieties is important. Therefore, we next explored if PapB can process msPapA when Cys and Asp are replaced with their dextrorotatory enantiomers (Figure S32A). Remarkably, with the leader-^D^CSANDA peptide, full conversion to the cross-linked peptide is seen, as evidenced by the loss of 2 Da (Figure S32B). With the leader-CSAN^D^DA peptide, significant substrate turnover is observed as well, but the conversion is not complete (Figure S32B). The leader-^D^CSAN^D^DA is processed inefficiently under these conditions, though some product is clearly observed in the MS. While it is possible to suggest that the small amount of product observed with this peptide is due to contaminating l-amino acids in the commercially available sources, that impurity would only amount to 1–2% of product formed. On the basis of the MS data, we observe at least ∼15% of the substrate is converted to product, arguing that the modification represents a *bona fide*^D^Cys to ^D^Asp thioether cross-link. Finally, CID MS/MS spectrometry shows a loss of 2 Da in each *y*-fragment after the C residue and in the single *b*-fragment after the d-residue in all three d-peptide scenarios (Figures S32C and S33–S35). Control experiments show that treatment with IAC results in no carboxymethylation in the C19^D^C peptide (Figure S36). In the case of the D23^D^D and C19^D^C/D23^D^D peptides, carboxymethylation is present upon IAC treatment due to incomplete turnover (Figures S37–S38). However, the carboxymethylated species do not show any evidence of a 2 Da loss, which is further evidence that the Cys thiol is participating in the newly installed bond in these unnatural peptides.

We next attempted to transpose the Cys and Asp residues by using a DSANCA motif attached to the leader. However, we were unable to observe any cross-linked product with the transposed peptides, either with l- or d-amino acids (Figure S39). These data support the notion that the active site has substantial flexibility with regard to the Cys but that the interaction with Asp limits the range of available productive conformations. Previous studies have shown that mutation of a conserved Arg residue in PapB (Arg372) to an Ala abolishes activity.^24^ While there are no structures of the substrate-bound enzyme, structural models suggest that this Arg residue could be near the carboxylate moiety of PapA. Inversion of the side chain would similarly eliminate the interaction leading to no cross-linking.

### PapB Processes Sequences Unrelated to the Wild-Type Peptide Sequence

The results presented in the previous sections highlight the remarkable lack of sequence specificity in PapB, suggesting that the enzyme may be able to cross-link virtually any sequence that is tethered to the leader sequence, so long as a Cys and a downstream Asp/Glu residue is present. As a proof of concept, we explored the use of PapB to generate an analogue of octreotide.

Octreotide is an FDA-approved drug used to treat excessive human growth hormone production, to control symptoms in several types of cancers, and to treat gastrointestinal bleeding.^30^ Octreotide has two d-amino acids, making it less susceptible to protease degradation *in vivo*.^31^

**Figure 5:**
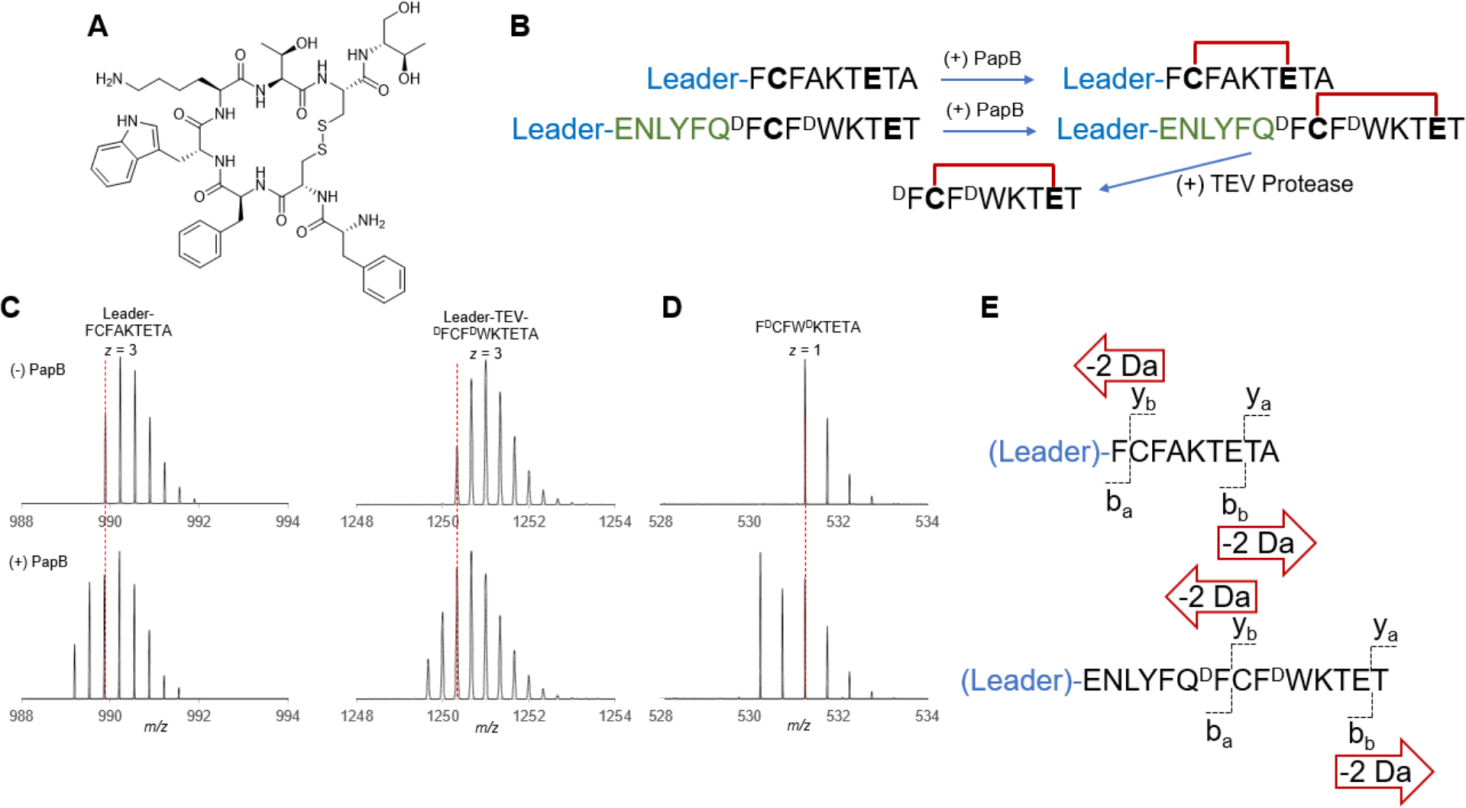
PapB catalyzes formation of octreotide analogues. (A) Structure of the FDA-approved therapeutic octreotide. (B) Schematic description of the designed peptides and the expected sites of modification. A TEV cleavage site is included in the second peptide to allow for cleavage upon modification by PapB. (C) The isotopic envelope of these peptides indicate that a mixed population of processed and unprocessed peptides is present after modification by PapB. (D) the TEV-cleaved peptide isotopic envelope reveals the anticipated 2 Da mass shift. (E) Tandem mass spectrometry shows the anticipated loss of 2 Da in each *y* fragment after the C and in each *b* fragment after the C-terminal E. A full description of the observed peptide fragments is seen in Figures S40–S41.

Octreotide is an 8-mer peptide with the sequence ^D^FCF^D^WKTCT, with d-amino acids at the first and fourth positions. The two C residues form a disulfide-linked macrocycle. The six cross-linking motifs in WT-PapA contain positively charged, nonpolar, polar uncharged, and bulky side chain residues,^24^ which, when taken with the successful cross-linking of expanded and contracted motifs shown above, suggested to us that PapB may be able to introduce disulfide mimetic bonds via a thioether in a variety of peptide substrates so long as a thiol and carboxylate moiety are present. As a proof of concept, we synthesized two octreotide analogues. Both designs dispensed with the C-terminal Cys in favor of an Glu, which we use to cross-link to the Cys with PapB. In the first design attempt, we simplified the sequence further by replacing ^D^W4 with Ala (Figure 5A). The octreotide analogue sequence was covalently attached to the PapA leader peptide by solid-phase peptide synthesis (SPPS). The second design contained only the C7E replacement, but to facilitate removal of the leader peptide, we incorporated an ENLYFQ sequence between the leader and the peptide to provide a TEV cleavage. The incubation of either of the designed octreotide analogues with PapB leads to formation of a new product. In each case, the product is 2 Da lighter than the starting material, consistent with the formation of a cross-link (Figure 5B). An intrapeptide disulfide can be eliminated as the source of this loss because only one Cys residue is present in the peptide. We note that the reaction is ∼75% complete with this analogue, as assessed from the isotopic envelope. However, the observed monoisotopic masses for each peptide products species are in good agreement with the expected monoisotopic masses for a single cross-link (<3 ppm, Figure 5B,C, Table S40). Subsequent MS/MS analysis corroborates the initial mass spectrometry data; the fragmentation pattern of the peptide depicts small fragments between the cross-linked Cys and Glu due to incomplete cross-linking. There is a clear 2 Da loss pattern in every *y*-fragment after the Cys and a 2 Da loss in every *b*-fragment after the C-terminal Glu with the modified peptide (Figures S41–S42).

Next, we attempted to use TEV protease to release the modified peptide to show the feasibility of the use of this method to generate a novel octreotide analogue. Other than Pro, the TEV protease can accommodate other amino acids at the P1′ position,^32^ but G or S are preferred. A crystal structure of a catalytically inactive form of TEV protease that was cocrystallized with an oligopeptide substrate revealed that the side chain of the residue at the P1′ position is partially exposed to solvent.^33^ To the best of our knowledge, d-amino acids have not been tested at the P1′ position. When treated with TEV protease, the peptide containing the TEV cleavage site undergoes cleavage to release the C-terminal fragment (Figure 5C). Our results highlight that ^D^F is tolerated the P1′ position. This proof-of-concept experiment demonstrates that PapB and TEV protease can be used together to generate therapeutic analogues from synthetic peptide substrates that contain both Cys and Asp/Glu residues, where PapB installs thioether bond(s) between Cys and Asp/Glu to replace disulfide bridges.

These findings support the notion that PapB can modify peptides with large spacing between the thiol and carboxylate moieties as well as sequences unrelated to PapA. These initial results indicate that PapB has utility as a bimoiety-dependent thioether installation tool. Some of the factors that make PapB an attractive tool include the observations that (1) it is tolerant of a variety of side chains spanning the peptide between the donor and acceptor Cys and Asp/Glu residues, (2) it shows flexibility toward the orientation as well as spacing of the carboxylate and thiol moieties, and (3) it tolerates substitutions outside the cross-linking motif allowing TEV recognition sequences being introduced to liberate the modified product.

## Discussion

In the 20 years since Sofia and co-workers established the RS superfamily,^7^ there has been an explosion of complex transformations that are attributable to RS enzymes. RS enzymes vastly expand the biochemical reaction repertoire because of their ability to activate C–H bonds for a variety of transformations, which can range from epimerizations to bonds to other carbon atoms or to heteroatoms. PapB catalyzes one such transformation, which entails activation of the carbon adjacent to a carboxylate moiety to cross-link to the thiol side chain of Cys.^24^ The mechanistic details of thioether cross-link formation remain to be elucidated. However, this manuscript highlights hitherto unknown promiscuity in PapA/B that will have implications in mechanism of substrate recognition. It remains to be seen to what extent the observations with PapA/B can be generalized to the RS enzymes that catalyze other transformations.

On the basis of all available structural and biochemical data on RiPP maturase proteins, one would expect that the leader sequence binds to the RiPP recognition element (RRE) domain and directs the peptide to the active site of the protein to be modified. Implicit in this is the assumption that the specificity in the substrate selection is governed by the binding energy of interactions with the leader sequence to the RRE domain. A conserved Asn side chain in the leader sequence has been proposed previously as being required for the peptide–RRE interaction.^24^ Our results that show PapB can accept substrates with Cys-to-Asp separation ranging from zero to six amino acids, and perhaps there is evidence for this in that the binding energy for interactions with the leader sequence is leveraged toward reactivity. However, when we initially began these studies, we assumed that the three amino acid separation likely meant that the peptide has a helical structure, as has been proposed previously,^29^ which would place the side chains of the Cys and Asp residues near one another in three-dimensional space. The observation that the enzyme can accept substrates with variable Cys-to-Asp spacing, however, suggests that the recognition relies on the specific side chain and not on the secondary structure. In other words, the enzyme specifically recognizes the Cys and Asp/Glu side chains. While there are no structural data, the PapB is homologous to SPASM superfamily enzymes that in addition to the RS cluster that binds and activates SAM also house at least two additional Fe/S clusters.^36−38^ It has been proposed that in the thioether cross-linking enzymes, the thiolate of the Cys can interact with one of the auxiliary Fe/S clusters.^19,34−38^ One can imagine that the recognition of the Asp/Glu may involve a hydrophilic or positively charged patch of residues. An Arg residue in PapB (Arg372) has previously been implicated by sequence alignments, a mutation of which abolished cross-linking activity.^24^ Therefore, the model for recognition that best fits our data is one where the peptide to be modified has only two albeit very specific interactions with the enzymes, outside of the leader sequence.

The observation that cross-linking efficiency is decreased when the separation is zero or six likely results from either constraint on the degrees of freedom in the shorter span or the presence of too many possible conformations in the longer separation, both of which would lead to fewer productive interactions between the residues to be cross-linked and the specific locations in which they bind. Additional evidence for the absence of significant sequence dependence, other than the identity of the Cys and Asp/Glu, is the fact that d-amino acids are tolerated. Outside of the leader sequence, we propose there are no specific interactions between the enzyme and the rest of the peptide other than the binding of the thiolate and carboxylate. As with other RS enzymes, one can anticipate that the binding occurs to place the 5′-position of SAM within or near van Der Waals radius of the H atom to be abstracted, which is in turn, within close proximity of the cross-linking Cys sulfur.

## Conclusions

While there have been several previous reports suggesting that RS RiPP maturases have varying levels of promiscuity,^29,39−42^ the range of substrates that are processed by PapB, which include not just modified donor and acceptor residues and sequence context, but also the ability to accept unnatural amino acids, foreshadows a much larger substrate scope. We should point out that perhaps our ability to observe this expanded range of reactivity is fortuitous and related to access to a highly active protein. Indeed, our estimate of turnover number for PapB in the s^–1^ range is only second to the most highly active RS enzymes, lysine aminomutase^43^ and pyruvate-formate lyase activating enzyme.^44^ This high level of activity allows us to detect even small amounts of turnover, though as has been shown throughout the paper, the level of activity for all substrates tested here is substantial.

To our knowledge, this study represents the first example of the use of a RS RiPP maturase toward synthesis of a molecule resembling a therapeutic agent. We do not know if the octreotide analogue synthesized here, where the disulfide is replaced with a thioether, will have biological activity. However, the ability to synthesize it in this manner underscores the potential for PapB and other related enzymes as tools in synthetic approaches to therapeutics

## Supporting Information Available

Detailed methods, chemical and biological reducing system kinetics experiments, results of d-amino acid cross-linking experiments, expected and observed monoisotopic masses for processed and unprocessed peptides, mass spectrometry data for IAC treated peptides, and the results of tandem mass spectrometry experiments (PDF)
Transparent Peer Review report available (PDF)

## Abbreviations

CID: collision-induced dissociation
CV: column volume
dAdo·: 5′-deoxyadenosyl radical
dT: dithionite
DTT: dithiothreitol
IAC: iodoacetic acid
msPapA: minimal substrate PapA
NRPS: nonribosomal peptide synthetases
ranthipeptides: radical non-α-carbon thioether peptides
RiPP: ribosomally synthesized and post-translationally modified peptide
RS: radical *S*-adenosyl-l-methionine;
