# Supplementary material for: Leveraging Substrate Promiscuity of a Radical S-Adenosyl-L-methionine RiPP Maturase toward Intramolecular Peptide Cross-Linking Applications
Source: ACS Cent Sci. 2022 Aug 1;8(8):1209–17. doi: 10.1021/acscentsci.2c00501 (PMC9413430; doi:10.1021/acscentsci.2c00501)
Supplement: Supplementary file 1 — oc2c00501_si_001.pdf [file oc2c00501_si_001.pdf]

Supporting Information

Leveraging substrate promiscuity of a radical SAM RiPP maturase towards intramolecular peptide crosslinking applications

Karsten A. S. Eastman, William M. Kincannon†, and Vahe Bandarian*

Department of Chemistry, University of Utah, 315 South 1400 East, Salt Lake City, Utah 84112, United States

Email: vahe@chem.utah.edu

Table of Contents

Methods Pages S4-S8

Figure S1. SDS-PAGE analysis of purified and reconstituted PapB Page S9

Figure S2. Fragmentation of msPapA Peptide Page: S10-S11

Figure S3. Time course of msPapA and PapB using dithionite or FldA/FPR/NADPH as reductant Page S12

Figure S4. Product formation is proportional with PapB concentration Page: S13

Figure S5. PapB is operating under saturating substrate conditions Page: S13

Table S6. Expected and observed monoisotopic masses for ring contraction and expansions Page: S14

Figure S7. Iodoacetic Acid Treatment for CX_0D recognition sequence Page: S15

Figure S8. Iodoacetic Acid Treatment for CX_1D recognition sequence Page: S16

Figure S9. Iodoacetic Acid Treatment for CX_2D recognition sequence Page: S17

Figure S10. Iodoacetic Acid Treatment for CX_4D recognition sequence Page: S18

Figure S11. Iodoacetic Acid Treatment for CX_5D recognition sequence Page: S19

Figure S12. Iodoacetic Acid Treatment for CX_6D recognition sequence Page: S20

Figure S13. Tandem mass spectrometry of unmodified and modified Leader-CX_0D Peptides Page: S21-S23

Figure S14. Tandem mass spectrometry of unmodified and modified Leader-CX_1D Peptides Page: S24-S26

Figure S15. Tandem mass spectrometry of unmodified and modified Leader-CX_2D Peptides Page: S27-S29

Figure S16. Tandem mass spectrometry of unmodified and modified Leader-CX_4D Peptides Page: S30-S32

Figure S17. Tandem mass spectrometry of unmodified and modified Leader-CX_5D Peptides Page: S33-S35

Figure S18. Tandem mass spectrometry of unmodified and modified Leader-CX_6D Peptides Page: S36-S39

Table S19. Expected and Observed Monoisotopic Masses for Leader Extension Peptides Page: S40

Figure S20. Tandem mass spectrometry analysis of unmodified and modified Leader-AAACSANDA peptides Page: S41-S44

Figure S21. Tandem mass spectrometry analysis of unmodified and modified Leader-AAACSANDACSANDA peptides Page: S45-S48

Figure S22. Tandem mass spectrometry analysis of unmodified and modified Leader-AAACSACDAADA peptides Page: S49-S52

Figure S23. Tandem mass spectrometry analysis of unmodified and modified Leader-AAAASACDAADA peptides Page: S53-S56

Figure S24. Tandem mass spectrometry analysis of unmodified and modified Leader-AAACSAADAAADA peptides Page: S57-S60

Figure S25. Iodoacetic acid treatment for unmodified and modified Leader-AAACSANDA peptides Page: S61

Figure S26. Iodoacetic acid treatment for unmodified and modified Leader-AAACSANDACSANDA peptides Page: S62

Figure S27. Iodoacetic acid treatment for unmodified and modified Leader-AAACSACDAADA peptides Page: S63

Figure S28. Iodoacetic acid treatment for unmodified and modified Leader-AAAASACDAADA peptides Page: S64

Figure S29. Iodoacetic acid treatment for unmodified and modified Leader-AAACSAADAADA peptides Page: S65

Table S30: Expected and observed monoisotopic masses of AMK-1057 and related peptides Page: S66

Figure S31: AMK-1057 *in vitro* modification tandem mass spectrometry Page: S66-S68

Figure S32. PapB Crosslinks ^{D}C and ^{D}D msPapA Peptides Page: S69

Figure S33. msPapA C19^{D}C Tandem Mass Spectrometry Page: S70-S72

Figure S34. msPapA D23^{D}D Tandem Mass Spectrometry Page: S73-S75

Figure S35. msPapA C19^{D}C/D23^{D}D Tandem Mass Spectrometry Page: S76-S78

Figure S36. Iodoacetic Acid Treatment for Leader-^{D}CSANDA peptide Page: S79

Figure S37. Iodoacetic Acid Treatment for Leader-CSAND^{D}A peptide Page: S80

Figure S38. Iodoacetic Acid Treatment for Leader-^{D}CSAND^{D}A peptide Page: S81

Figure S39. Mass Spectra of msPapA peptides with interchanged D and C residues Page: S81

Table S40. Expected and observed monoisotopic masses for octreotide analogs Page: S83

Figure S41. Tandem Mass Spectrometry analysis of the octreotide analog Leader-FCFAKTETA Page: S83-S84

Figure S42. Tandem Mass Spectrometric analysis of modified Leader-TEV-OctreotideC7E peptide Page: S85-S86

References Page: S87

Methods

No unusually high or unexpected safety hazards were encountered.

Cloning and Expression of PapB

The plasmids PapB[1] and pPH151 were co-transformed into *Escherichia coli* BL21 (DE3) T1 resistant cells (NEB C2527). The plasmid pPH151 contains the *suf* operon that encodes for sufABCDE proteins that aid in sulfur liberation, act as a Fe-S scaffold, and donate Fe-S clusters to apo proteins. The *suf* operon is frequently included with radical SAM enzymes as it assists in assembling iron-sulfur clusters in heterologously expressed proteins[2-3]. The transformation mixture was suspended in SOC recovery media and shaken at 200 rpm for 1 h at 37 °C. The mixture was plated on agar Lennox broth (LB) plates containing 34 µg/mL chloramphenicol and 34 µg/mL kanamycin and placed in an oven set to 37 °C for 16 h. An overnight culture (0.15 L) of LB containing 34 µg/mL chloramphenicol and 34 µg/mL kanamycin was inoculated with a single colony from the plate. Twelve aliquots (12 mL each) of overnight culture were used to inoculate twelve 2.8 L Fernbach flasks containing 1 L each of LB supplemented with 34 µg/mL chloramphenicol and 34 µg/mL kanamycin. The cultures were grown at 37 °C and 180 rpm to an OD600nm of ~0.35, at which point 0.1 mM iron(III) chloride (0.1 mM) and L-cysteine hydrochloride monohydrate (0.1 mM) were added. At OD_{600nm} of ~0.5, the flasks were immersed in an ice bath and cooled for 20 min before inducing with 1 mM isopropyl β-D-1-thiogalactopyranoside (IPTG). The cultures were grown overnight (~16 h), and the cells were harvested by centrifugation at 6500 x g. Typical yield is ~45 g of wet cell paste per 12 L of growth. The cell pellets were flash-frozen in liquid N2 and stored at -80 °C until use.

Purification of PapB

PapB was purified inside of a Coy Laboratories anaerobic chamber maintained with a 98% N_2/2% H_2 atmosphere. Cell paste (15 g) was resuspended in a metal beaker with 0.1 L of 0.05 M KPi (pH 7.4) buffer containing 0.5 M KCl, 0.05 M imidazole, 20% glycerol (v/v) 0.1 mg/mL lysozyme, 10 µg/mL DNAse and 2 cOmplete EDTA-free Protease Inhibitor Cocktail tablets (Fisher Scientific NC0939481). The suspension was stirred for 30 min on ice after which the cells were lysed with a Branson digital sonifier operated at 50% amplitude for a total of 17 min (25 s on/35 s off) while stirring on ice. The resulting liquid was centrifuged at 18,442 x g for 45 min at 4 °C. Three 5 mL HisTrap HP columns (GE healthcare) charged with nickel sulfate were serially connected and equilibrated with loading buffer containing 0.05 M KPi (pH 7.4), 0.5 M KCl, 20% glycerol (v/v) and 0.05 M imidazole. The clarified lysate was loaded onto the columns at 3 mL/min. The columns were washed with eight column volumes (CV) of loading buffer and PapB was eluted with a linear gradient over 8 CV to 0.5 M imidazole in the loading buffer. Fractions containing PapB were identified by brown color and sodium dodecyl sulfate polyacrylamide gel electrophoresis (SDS-PAGE) gel.

The pooled fractions were further purified by an amylose resin (NEB E8022S) column equilibrated in loading buffer containing 0.05 M KPi (pH 7.4), 0.5 M KCl, and 0.05 M imidazole. The column was washed with 0.15 L of loading buffer and eluted with loading buffer containing 10 mM maltose. The resulting dark brown fractions were pooled and solid dithiothreitol (DTT) powder was added to combined fractions to a final concentration of 2 mM. An aliquot (1 mL) of 90 µM TEV protease was added, and the mixture was stirred for 14 h at room temperature. The cleaved MBP was removed from PapB through three serially connected 5 mL HisTrap HP columns equilibrated in loading buffer. The flowthrough from this column contained cleaved PapB. The resulting PapB

protein was desalted into buffer containing 0.05 M PIPES•NaOH (pH 7.4), 0.3 M NaCl, 2 mM DTT, and 20% glycerol (v/v). The concentration of PapB was determined by the Bradford method using bovine serum albumin (BSA) as a standard. PapB was reconstituted by mixing 12 molar equivalents of 0.1 M $FeCl_3$ hexahydrate and Na_2S nonahydrate as follows. Aliquots (5 µL) of the $FeCl_3$ hexahydrate and Na_2S were added individually, allowing 15 sec between additions to ensure thorough mixing. The $FeCl_3$ was added to completion first, following by the addition of the Na_2S. The reconstitution mixture was stirred for 4 h at room temperature. The resulting solution was centrifuged for 10 min at 16,000 *xg* for 10 min to remove any debris and desalted on a BioGel P6 DG desalting gel 100-200 mesh (wet) (Bio-Rad) into buffer containing 0.05 M PIPES•NaOH (pH 7.4), 0.3 M NaCl, 2 mM DTT, and 20% glycerol (v/v). The protein was concentrated to ~3 mL with an Amicon concentrator under N_2 with a YM-10 membrane (Millipore).

The reconstituted PapB was further purified by a Cytiva XK26 (1000 mm) S-300 column equilibrated with buffer containing 0.05 M PIPES•NaOH (pH 7.4), 0.3 M KCl, 2 mM DTT, and 10% glycerol (v/v). The protein was eluted isocratically at 2.7 mL/min, and fractions containing PapB were identified by dark brown color and visual inspection of a Coomassie-stained SDS-PAGE gel. The pooled fractions were concentrated to ~0.5 mL. Aliquots were flash-frozen in liquid N2 and stored at -80 °C. PapB was quantified by Bradford assay with BSA as a standard. A typical yield from the purification outlined above is 16.5 mg pure protein for 15 g wet cell paste.

Amino Acid Analysis and Iron Concentration Determination

The correction factor for the Bradford assays was determined by direct amino acid analysis on three independent preparations of the protein. Amino acid analysis was carried out by the Molecular Structure Facility at the University of California-Davis as follows. A 0.1 mL aliquot of concentrated PapB was desalted into solution containing 10 mM NaOH using an Illustra NICK column (GE Healthcare). The protein samples were hydrolyzed in a solution containing 6 M HCl and 1% phenol at 110 °C in a vacuum and resuspended in a norleucine solution as an internal standard. The PapB samples were analyzed by Hitachi 8800 amino acid analyzer that was calibrated with amino acid standards for protein hydrolysate on the Na-based Hitachi 8800 (Sigma, A-9906). These standards were verified by the National Institute of Standards and Technology (NIST) standard reference material 2389a. PapB samples were sent through a Concise ion-exchange column (AminoSep Beckman Style Na+, part #AAA-99-6312) with a secondary ninhydrin reaction for detection using Pickering Na buffers. The correction factor for the Bradford assays was determined to be 0.60 based on results from the three independent purifications and this factor was used in all subsequent protein concentration determinations to correct the values obtained from the Bradford determinations.

The iron content of reconstituted PapB was determined through inductively coupled plasma-mass spectrometer (ICP-MS) on the same three separate enzyme preparations. This was done at the Center for Water, Ecosystems and Climate Science in the Department of Geology and Geophysics at the University of Utah as follows. PapB preparations were diluted to a concentration of 2-5 µM with 10% trace metal grade nitric acid before submission. The iron concentration was performed with a triple quadrupole inductively coupled plasma-mass spectrometer (ICP-MS, Agilent 8900, Santa Clara, CA). A 10 nm In/mL was added as an internal standard. An external calibration curve was prepared from 1000 mg/L single elemental standard (Inorganic Ventures, Christiansburg, VA). Fe Concentrations in six calibration solutions were 0, 8.3, 20.7, 66.2, 165.5 and 331.1 ng Fe/mL; all solutions contained 10 ng In/mL. The blanks, calibration solutions and diluted samples were run by ICP-MS using a double-pass quartz spray

chamber, PTFE nebulizer and dual-syringe introduction system (Teledyne, AVX72000), platinum cones, and sapphire injector in a quartz platinum-shielded torch. In and Fe were detected at masses of 115 and 56, with a flow of 8 mL He/min in a collision cell. The Certified Reference Manual CRM 1643f (National Institute of Standards and Technology, Gaithersburg, MD) was diluted 1:20 and run with the samples as well as the calibration curve as a quality control for the calibration. The Fe in the CRM 1643f was measured to be 10% within the certified value.

TEV Protease Purification

SG1200008 pRARE chemically competent cells were transformed with pNB512[4]. The transformation was suspended in SOC recovery media and shaken at 200 rpm for 45 min at 37 °C. The mixture was plated on agar Lennox broth (LB) plates containing 34 ug/mL chloramphenicol and 100 μg/mL ampicillin and placed in an oven set to 37 °C for 16 h. An overnight culture (0.15 L) of LB containing 34 μg/mL chloramphenicol and 34 μg/mL ampicillin was inoculated with a single colony from the plate. Twelve aliquots (0.010 L each) of overnight culture were used to inoculate twelve 2.8 L Fernbach flasks containing 1 L each of LB supplemented with 34 μg/mL chloramphenicol and 100 μg/mL ampicillin. The cultures were grown at 37 °C and 175 rpm to an OD_{600nm} of ~0.49, at which point 1 mM IPTG was added to each flask and the temperature was turned down to 16 °C. The cultures were grown overnight (~16 h), and the cells were harvested by centrifugation at 6500 *xg*. The cell pellets were flash-frozen in liquid N_2 and stored at -80 °C until use.

Cell paste (15 g) was resuspended in a metal beaker with 0.1 L of 0.05 M KPi (pH 7.4) buffer containing 0.5M KCl, 0.05 M imidazole, 100 μg/mL lysozyme, 10 μg/mL PMSF, and 20% (v/v) glycerol. The suspension was stirred for 2 h at 4 °C. The cells were lysed with a Branson digital sonifier operated at 50% amplitude for a total of 15 min (10 s on/20 s off) while stirring on ice. The resulting liquid was centrifuged at 18,442 *xg* for 50 min at 4 °C. Two 5 mL HisTrap HP columns (GE healthcare) charged with nickel sulfate were serially connected and equilibrated with loading buffer containing 0.05 M KPi (pH 7.4), 0.5 M KCl, and 0.05 M imidazole. The clarified lysate was loaded onto the columns at 3 mL/min. The columns were washed with eight column volumes (CV) of loading buffer and TEV protease was eluted with a linear gradient over 8 CV to 0.5 M imidazole in the loading buffer. Fractions containing TEV protease were identified by SDS-PAGE, pooled and dialyzed three times against 4 L of 0.05 M KPi (pH 7.4), 0.5 M KCl, 0.05 M imidazole and 20% glycerol. The dialyzed protein was concentrated to a minimal volume followed by the addition of glycerol to a final concentration of 50% glycerol (v/v). The aliquots were flash frozen in liquid nitrogen and stored at -80 °C until use.

Synthesis of Minimal Substrate PapA (msPapA) and Variants

PapA peptides were synthesized on either a PS3 peptide synthesizer (Protein Technologies Inc.) or a Prelude peptide synthesizer (Protein Technologies Inc.). Compared to the previously reported msPapA peptide[1], the N-terminal methionine was removed in all peptide syntheses. The syntheses used standard Fmoc procedures from the manufacturer and were carried out on a 0.025 mmol scale. All natural Fmoc-amino acids were purchased from Protein Technologies Inc. N-Alpha-Fmoc-S-trityl-D-cysteine and Fmoc-D-aspartic acid α-*tert*-butyl ester were purchased from Chem Impex (04314). For the synthesis,150 mg of 2-chlorotrityl chloride resin 100-200 mesh (ChemPep) was loaded with 9.3 mg of Fmoc-Ala-OH (~0.2 mmol/g resin). The resin was washed three times with 5 mL DMF and three times with 5 mL dichloromethane (DCM). The 9.3 mg of Fmoc-Ala-OH was dissolved in 1 mL of 1:1 dichloromethane (DCM):N,N-dimethylformaide (DMF)

with 0.15 mmol diisopropylethylamine (DIPEA). This solution was added to the resin and gently shaken for 1 h. The Fmoc-Ala/DIPEA solution was then removed, and the resin was washed three times with 5 mL of DCM. The uncapped sites on the resin were capped by washing the resin with 20 mL of 17:2:1 DCM:methanol:DIPEA. The resin was then washed three times with 5 mL of DCM and three times with 5 mL of DMF. The resin was then transferred to the reaction vessel.

All Fmoc-amino acids (0.15 mmol, 6 equivalents) were coupled by *in situ* activation with N-[(dimethylamino)-1H-1,2,3-triazo[4,5-b]pyridin-1-ylmethylene]-N-methylmethanaminium hexafluorophosphate-N-oxide (HATU) (0.15 mmol, 6 equivalents; ChemPep) in 0.6 M *N*-methylmorpholine. The peptides were deprotected and cleaved from the resin by adding 5 mL of cleavage solution (87.5% (v/v) TFA, 5% (v/v) thioanisole, 3% (v/v) ethane dithiol, 2.5% (v/v) triisopropylsilane, and 2% (v/v) anisole) followed by stirring for 2 h at room temperature. The cleavage reaction was filtered into 30 mL of ice-cold diethyl ether to precipitate the peptide. The solution was poured over a Büchner funnel filter and vacuumed to collect the peptide precipitate. The peptide dried on the vacuum for 15 min before being washed with 80 mL of ice-cold diethyl ether. After drying for an additional hour, the peptide was resuspended in 20 mL of water and sonicated for 15 min to aid in the dissolution of the peptide. The solution was then flash frozen in liquid nitrogen and lyophilized.

The peptides were purified using high-performance liquid chromatography (HPLC) with a Phenomenex Jupiter C18 preparative column (21.2 mm x 250 mm, 5 µm particle size, 300Å pore size) with buffer A as 0.1% trifluoroacetic acid (TFA, HPLC grade) in nanopure water and buffer B as 0.1% TFA (HPLC Grade) in acetonitrile (ACN, HPLC grade). The separation was carried out at a flow rate of 5 mL/min with a linear gradient of buffer A from 88 to 60% over 65 min. Fractions were analyzed by LC-MS using one of two HPLC-MS/MS setups (Vanquish UHPLC with a diode array detector connected to a Q-Exactive or an Ultimate 3000 HPLC with a diode array detector interfaced to a LTQ OrbiTrap XL mass spectrometer) fitted with a Hypersil GOLD C18 column (2.1 mm x 150 mm, 1.9 µm particle size) for separations at 0. 2mL/min. The LC-MS program for peptide fraction identification was set up as follows: buffer A was LC-MS Optima water (Fisher)/0.1% (v/v) LC-MS Optima TFA (Fisher) and buffer B was LC-MS Optima acetonitrile (Fisher)/0.1% (v/v) LC-MS Optima TFA (Fisher). The 12 min separation consisted of washing the column with 100% A for 3 min, followed by a linear gradient to 100% B from 3 to 6 min, followed by washing the column with 100% B from 6 to 9 min, and finally reequilibration in 100% A from 9 to 12 min. The MS detectors operated in positive ion mode and the FT analyzer settings are as follows: 70,000 resolution for the Q-Exactive and 100,000 resolution for the LTQ OrbiTrap, 1 microscan, and 200 ms maximum injection time. MS data analysis used Xcalibur software (Thermo Fisher).

Enzymatic Reactions of msPapA peptides with PapB

Assays were conducted in a Coy Laboratories anaerobic chamber with 98% N_2/2% H_2 atmosphere at room temperature. All reactions contained 0.05 M PIPES•NaOH (pH 7.4), 2mM DTT, 2.4 mM SAM (enzymatically synthesized and purified as previously described[5]), ~100-400 µM msPapA variants (concentration determined by peptide dry weight or by spectroscopic analysis in the case of Y19W), and 430 nM-10 µM PapB. Either dithionite (dT) or flavodoxin (FldA), flavodoxin reductase (FPR) and NADPH were used to reduce PapB. For the assays that used chemical reductant, the total concentration of dT was 2mM. For the assays that used the biological reducing system, the mixtures contained 25 µM FldA, 2 µM FPR, and 2 mM NADPH.

The total volume of the reactions ranged from 0.1 mL for initial screenings to 0.5 mL for MS/MS Collision Induced Dissociation (CID) fragmentation experiments described below. Control reactions in the absence of dT, SAM and PapB were also conducted. Reactions were initiated with the addition of PapB and quenched at times ranging from 15 s to 2 h by the addition of 10% of the reaction volume of 30% (w/v) trichloroacetic acid (TCA, ACS grade). The samples were centrifuged at 16,000 *xg* for 10 min in a microcentrifuge to pellet the precipitated PapB.

Alkylation of msPapA Peptides and Variants

After initial incubation, half of the enzymatic reaction and half of the control reaction was aliquoted for alkylation by iodoacetic acid. A 500 mM stock of iodoacetic acid (IAA) was prepared in the dark and added to the enzymatic reaction to a final concentration of 10mM (5-fold excess over the DTT concentration). These reactions were allowed to incubate in the dark for six additional hours before quenching with the addition of 10% of the reaction volume of 30% (w/v) TCA. The samples were then centrifuged at 16,000 *xg* for 10 min in a microcentrifuge to pellet the precipitated PapB.

TEV Protease Cleavage of Peptides

Where TEV cleavage was necessary, 90 µM TEV protease was added directly to the full PapB assay after initial incubation in a 1:1 (v/v) ratio. The TEV-assay combination incubated for 4 h before quenching by the addition of 10% of the reaction volume of 30% (w/v) TCA. The samples were then centrifuged at 16,000 *xg* for 10 min in a microcentrifuge to pellet the precipitated PapB and TEV protease.

U/HPLC-MS Analysis of Enzymatic Reactions and Controls

The assays were analyzed using either a Vanquish UHPLC with a diode-array detector connected to a Q-Exactive mass spectrometer or an Ultimate 3000 HPLC with a diode-array detector connected to a LTQ OrbiTrap XL mass spectrometer. Each was operated in positive ion mode, the FT analyzer was set to 100,000 resolution, 1 microscan, and 200 ms maximum injection time. Xcalibur software was used to analyze data. A 20 µL aliquot was injected onto a Hypersil GOLD C18 column (2.1 mm x 150 mm, 1.9 µm particle size) (Thermo Fisher) pre-equilibrated in 0.1% (v/v) LC-MS Optima TFA (Fisher in LC-MS Optima water (Fisher). Chromatographic steps were carried out at 0.2 mL/min with buffer A containing 0.1% (v/v) TFA in Optima water and buffer B containing Optima grade acetonitrile with 0.1% (v/v) TFA. The separation consisted of washing with 100% A from 0 to 3 min, followed by a linear gradient from 100% to 0% A from 3 to 6 min, washing with 0% A from 6 to 10 min, and re-equilibration with 100% A from 10 to 14 min.

Collision-Induced Dissociation (CID) Fragmentation of Unmodified and Modified PapB

Enzymatic reactions were conducted on a 0.5 mL scale as described above to obtain sufficient material. After quenching the reaction with TCA and centrifugation to remove precipitated protein, the reaction mixtures were desalted using C18 ZipTips (Millipore) following the manufacturer's protocols. The analyzer was first tuned to the mass of each msPapA peptide. The 3^+ charge state corresponding to each msPapA peptide was isolated in the CID cell using an isolation width of 1.7-2.4 *m/z* (depending on complete or incomplete peptide turnover), 0.1 ms activation time, a resolution of 70,000, and fragmented using a Normalized Collision Energy (NCE) of 25. The fragments were analyzed using mMass software.

Figure S1. SDS-PAGE analysis of purified and reconstituted PapB on a 12% crosslinked gel.

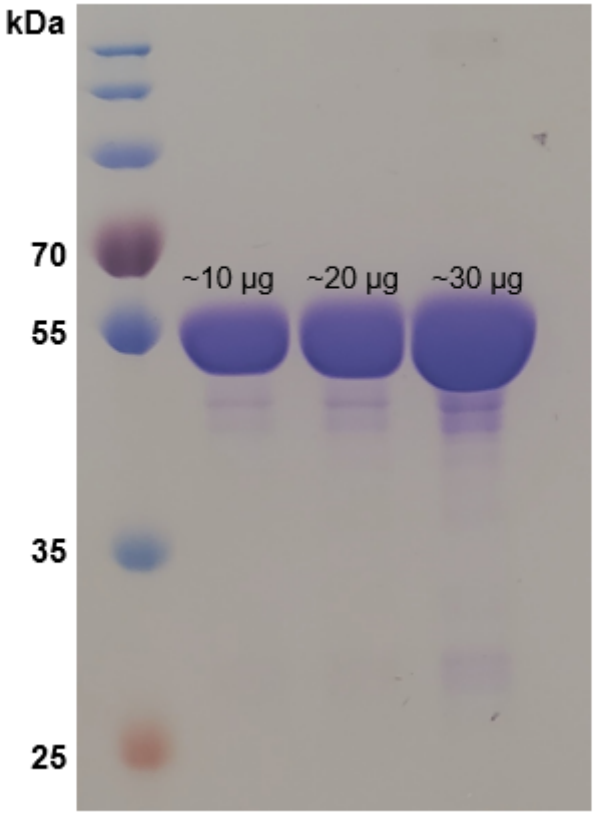

Figure S2. Fragmentation of msPapA Peptide

Leader-CSANDA Peptide: msPapA
Sequence: LKQINVIAGVKEPIRAYG**C**SAN**D**A

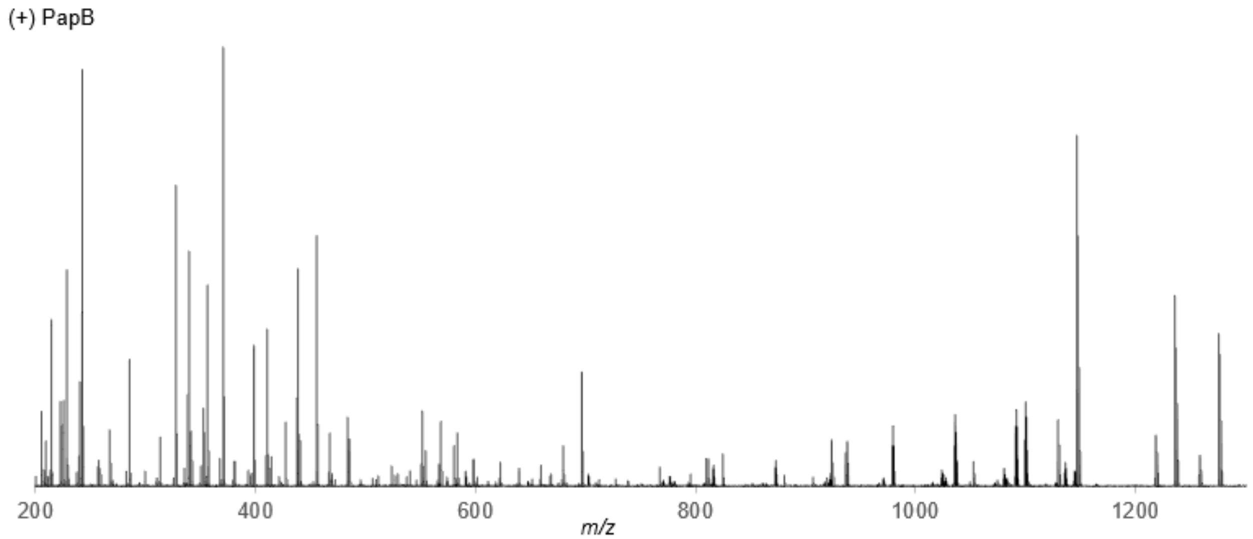

Unhighlighted indicates *z* = 1 charge state.
Gold highlight indicates *z* = 2 charge state.
Teal highlight indicates *z* = 3 charge state.
* Indicates the fragment originated from the modified peptide.

Ion	Sequence	Expected Monoisotopic Mass	Observed Monoisotopic Mass	Ppm Error
*b*2*	LK*	242.1863	242.1856	-2.89
*b*3*	LKQ*	370.2449	370.2442	-1.89
*b*4*	LKQI*	483.3289	483.3282	-1.44
*b*5*	LKQIN*	597.3719	597.3712	-1.17
*b*6*	LKQINV*	696.4403	696.4391	-1.72
*b*7*	LKQINVI*	809.5244	809.5232	-1.48
*b*8*	LKQINVIA*	880.5615	880.5606	-1.02
*b*9*	LKQINVIAG*	937.5829	937.5822	-0.74
*b*10*	LKQINVIAGV*	1036.6513	1036.6502	-1.06
*b*11*	LKQINVIAGVK*	1164.7463	1164.7461	-0.17
*b*12*	LKQINVIAGVKE*	1293.7889	1293.7885	-0.31
*b*13*	LKQINVIAGVKEP*	695.9245	695.9245	0
*b*14*	LKQINVIAGVKEPI*	752.4665	752.4646	-2.52
*b*15*	LKQINVIAGVKEPIR*	830.5171	830.5162	-1.08
*b*16*	LKQINVIAGVKEPIRA*	866.0356	866.0344	-1.39
*b*17*	LKQINVIAGVKEPIRAY*	947.5673	947.5666	-0.74
*b*18*	LKQINVIAGVKEPIRAYG*	976.0780	976.0778	-0.20
*b*19*	(-1 Da) LKQINVIAGVKEPIRAYG**C***	1027.0787	Not Found	N/A
*b*20*	(-1 Da) LKQINVIAGVKEPIRAYG**C**S*	1106.1133	Not Found	N/A
*b*21*	(-1 Da) LKQINVIAGVKEPIRAYG**C**SA*	1127.6043	Not Found	N/A

*b*22*	(-1 Da) LKQINVIAGVKEPIRAYG**C**SAN*	1163.1347	Not Found	N/A
*b*23*	(-2 Da) LKQINVIAGVKEPIRAYG**C**SAN**D***	813.7653	813.7641	-1.47
[M]	(-2 Da) LKQINVIAGVKEPIRAYG**C**SAN**D**A*	843.4478	843.4468	-1.18
*y*23*	(-2 Da) KQINVIAGVKEPIRAYG**C**SAN**D**A*	805.7532	805.7520	-1.49
*y*22*	(-2 Da) QINVIAGVKEPIRAYG**C**SAN**D**A*	1144.0786	1144.0780	-0.52
*y*21*	(-2 Da) INVIAGVKEPIRAYG**C**SAN**D**A*	1080.0493	1080.0490	-0.27
*y*20*	(-2 Da) NVIAGVKEPIRAYG**C**SAN**D**A*	1023.5073	1023.5069	-0.39
*y*19*	(-2 Da) VIAGVKEPIRAYG**C**SAN**D**A*	966.4858	966.4852	-0.62
*y*18*	(-2 Da) IAGVKEPIRAYG**C**SAN**D**A*	916.9516	916.9512	-0.43
*y*17*	(-2 Da) AGVKEPIRAYG**C**SAN**D**A*	860.4096	860.4087	-1.04
*y*16*	(-2 Da) GVKEPIRAYG**C**SAN**D**A*	824.8910	824.8902	-0.97
*y*15*	(-2 Da) VKEPIRAYG**C**SAN**D**A*	796.3803	796.3782	-2.63
*y*14*	(-2 Da) KEPIRAYG**C**SAN**D**A*	746.8461	746.8451	-1.34
*y*13*	(-2 Da) EPIRAYG**C**SAN**D**A*	682.7986	682.7983	-0.44
y12*	(-2 Da) PIRAYG**C**SAN**D**A*	1235.5473	1235.5469	-0.32
*y*11*	(-2 Da) IRAYG**C**SAN**D**A*	1138.4946	1138.4941	-0.44
*y*10*	(-2 Da) RAYG**C**SAN**D**A*	1025.4105	1025.4103	-0.19
*y*9*	(-2 Da) AYG**C**SAN**D**A*	869.3094	869.3081	-1.49
*y*8*	(-2 Da) YG**C**SAN**D**A*	798.2723	798.2708	-1.88
*y*7*	(-2 Da) G**C**SAN**D**A*	635.2090	635.2080	-1.57
*y*6*	(-2 Da) **C**SAN**D**A*	578.1875	Not Found	N/A
*y*5*	(-1 Da) SAN**D**A*	476.1861	Not Found	N/A
*y*4*	(-1 Da) AN**D**A*	389.1541	Not Found	N/A
*y*3*	(-1 Da) N**D**A*	318.1170	Not Found	N/A
*y*2*	(-1 Da) **D**A*	204.0741	Not Found	N/A

Figure S3 Comparison of activity of PapB processing Y17W msPapA with dithionite or FldA/FPR/NADPH as reductant.

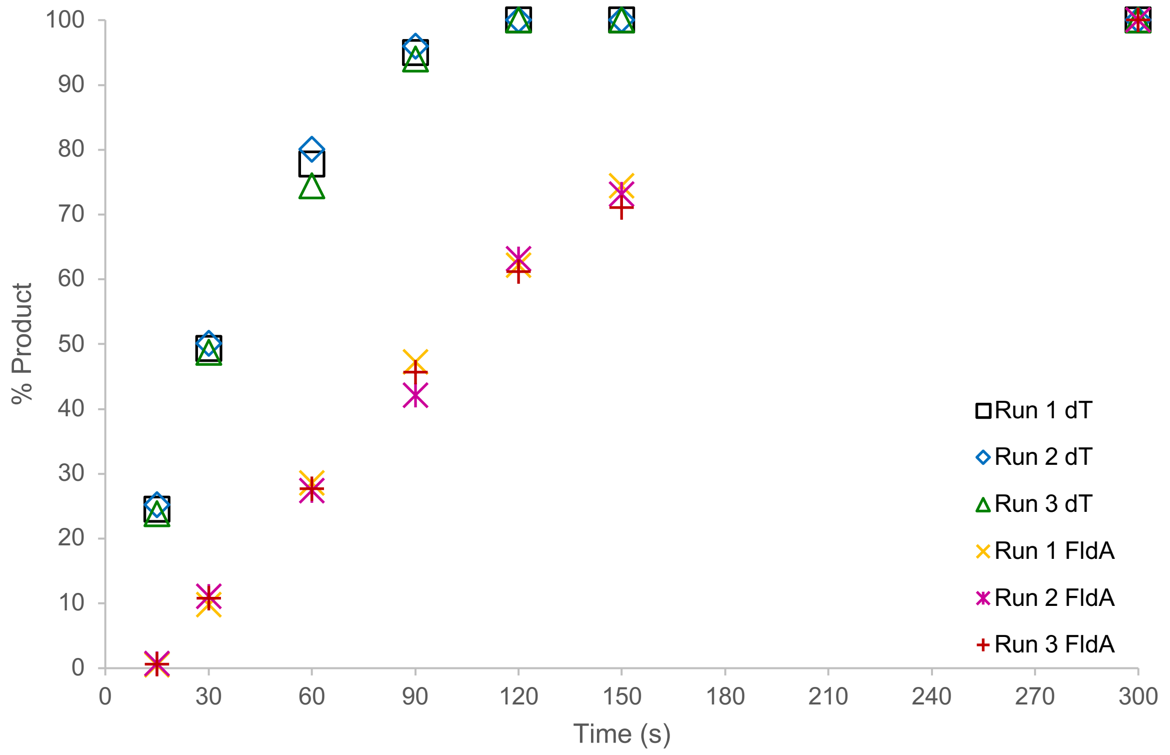

Three replicate runs were conducted using either dT or the biological reducing system of FldA/FPR/NADPH. At each timepoint, an aliquot was taken from the initial assay batch quenched with TCA. Both the biological reducing system and the chemical reducing system had 100% substrate conversion after 300 s.

Figure S4. Product formation is proportional with PapB concentration

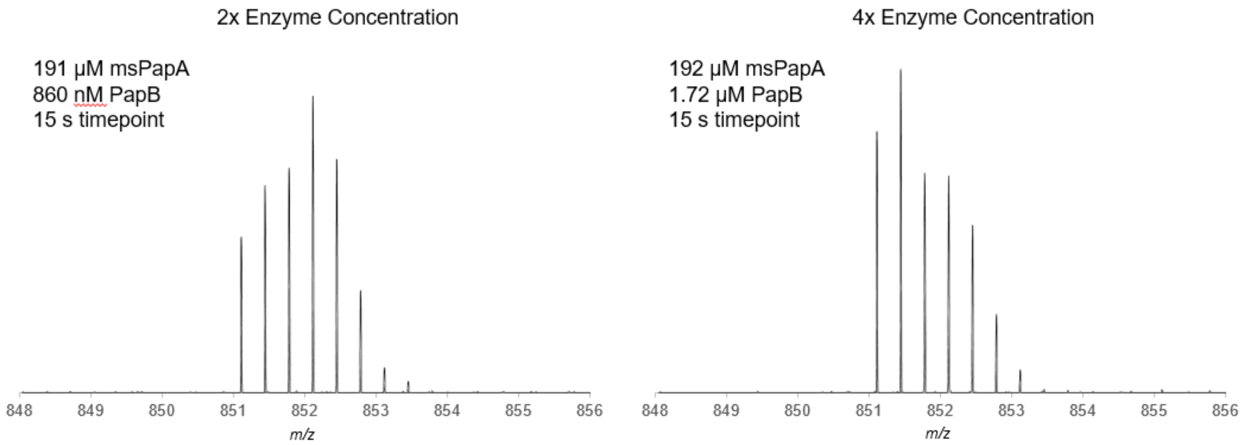

Figure S5. PapB is operating under saturating substrate conditions

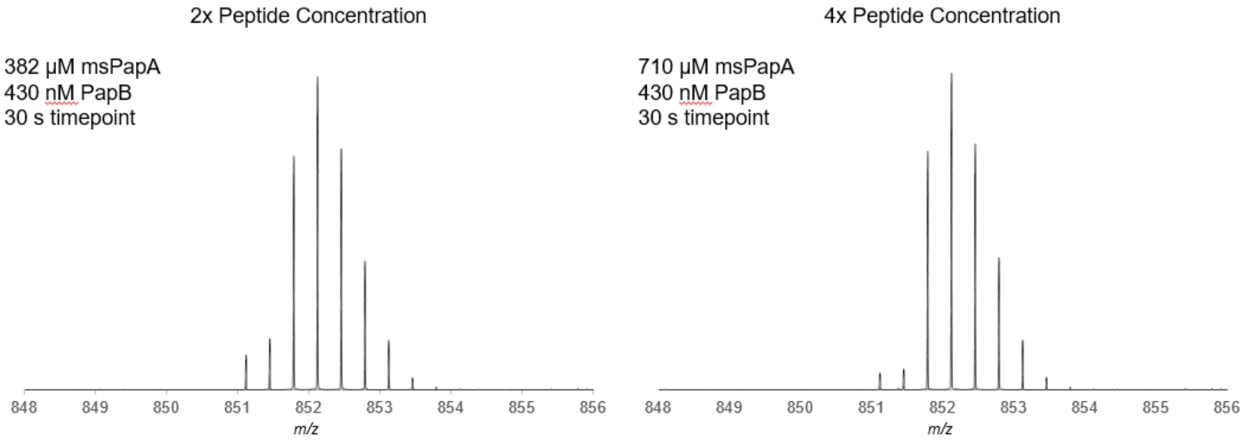

Table S6. Expected and observed monoisotopic masses for ring contraction and expansions
Leader = LKQINVIAGVKEPIRAYG

Sequence	Expected Monoisotopic Mass	Observed Monoisotopic Mass	Ppm Error
msPapA<br>(CX$_3$D)	(-) PapB: 844.1197<br>(+) PapB (-2Da): 843.4478	844.1201<br>843.4485	0.474<br>0.823
Leader-CDSNNAANA<br>(CX$_0$D)	(-) PapB: 943.8247<br>(+) PapB (-2Da): 943.1555	943.8253<br>943.1573	0.645<br>1.908
Leader-CSDNNAAA<br>(CX$_1$D)	(-) PapB: 905.8131<br>(+) PapB (-2Da): 905.1412	905.8166<br>905.1418	3.863<br>0.662
Leader-CSNDAAA<br>(CX$_2$D)	(-) PapB: 867.7988<br>(+) PapB (-2Da): 867.1269	867.7999<br>867.1278	1.267<br>1.038
Leader-CSAANDA<br>(CX$_4$D)	(-) PapB: 867.7988<br>(+) PapB (-2Da): 867.1269	867.7972<br>867.1287	-1.843<br>2.076
Leader-CSAAANDA<br>(CX$_5$D)	(-) PapB: 891.4778<br>(+) PapB (-2Da): 890.8059	891.4777<br>890.8065	-0.112<br>0.674
Leader-CSAAAANDA<br>(CX$_6$D)	(-) PapB: 915.1568<br>(+) PapB (-2Da): 914.4850	915.1564<br>914.4846	-0.437<br>-0.437

z = 3 in all cases

Figure S7. Iodoacetic acid treatment for CX_0D recognition sequence
Leader = LKQINVIAGVKEPIRAYG

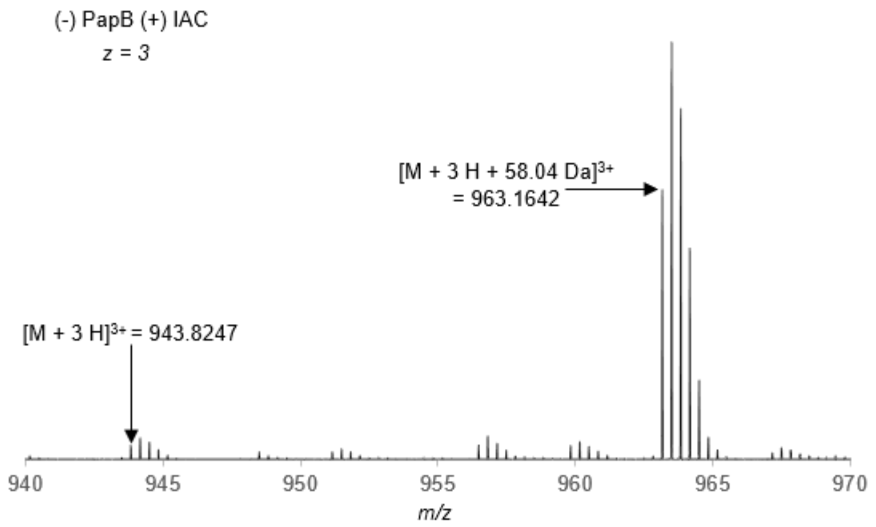

Sequence	Expected Monoisotopic Mass	Observed Monoisotopic Mass	Ppm Error
Leader-CDSNNAANA (CX_0D)	(-) PapB: 943.8247	943.8229	-1.90
Leader-CDSNNAANA (CX_0D) + S-carboxymethyl	(-) PapB + (58.04 Da): 963.1625	963.1642	1.77

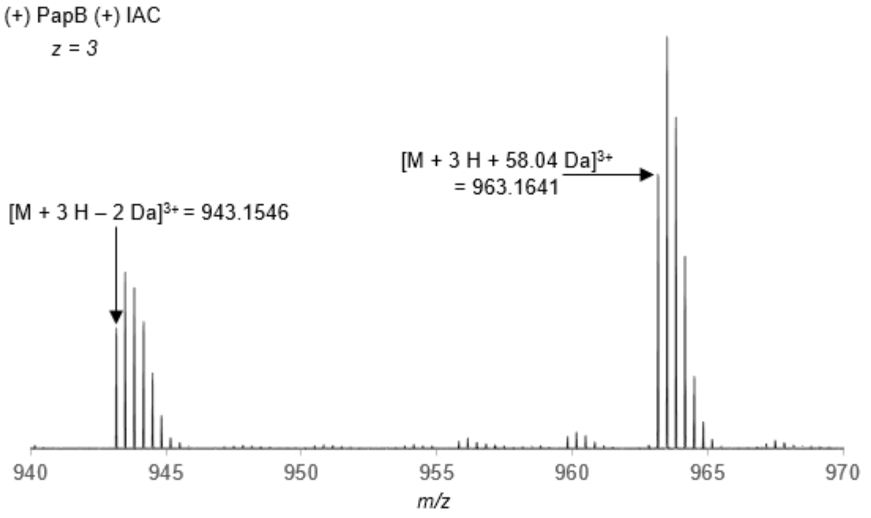

Sequence	Expected Monoisotopic Mass	Observed Monoisotopic Mass	Ppm Error
Leader-CDSNNAANA (CX_0D)	(+) PapB - (2 Da): 943.1555	943.1546	-0.95
Leader-CDSNNAANA (CX_0D) + S-carboxymethyl	(-) PapB + (58.04 Da): 963.1625	963.1641	1.66

Figure S8. Iodoacetic acid treatment for CX_1D recognition sequence
Leader = LKQINVIAGVKEPIRAYG

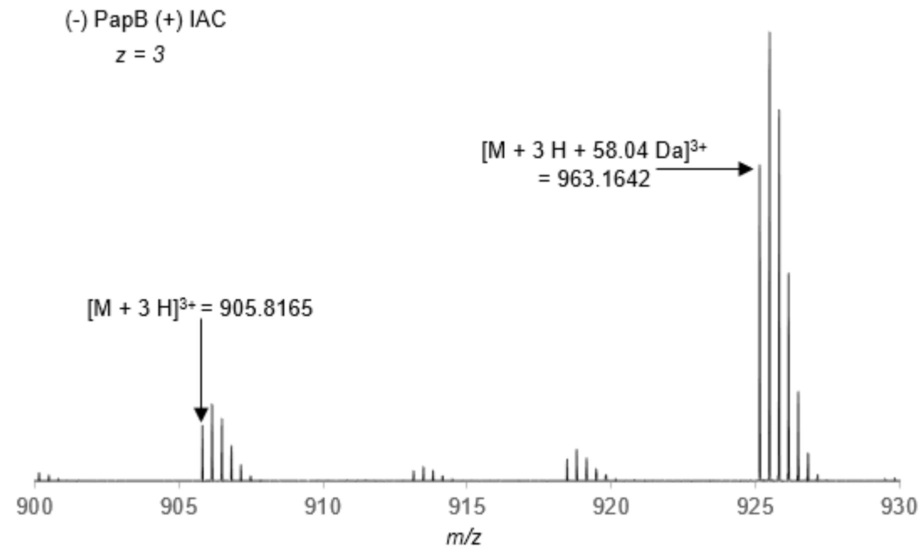

Sequence	Expected Monoisotopic Mass	Observed Monoisotopic Mass	Ppm Error
Leader-CSDNNAAA (CX_1D)	(-) PapB: 905.8131	905.8165	3.75
Leader-CSDNNAAA (CX_1D) + S-carboxymethyl	(-) PapB + (58.04 Da): 925.1482	925.1488	0.65

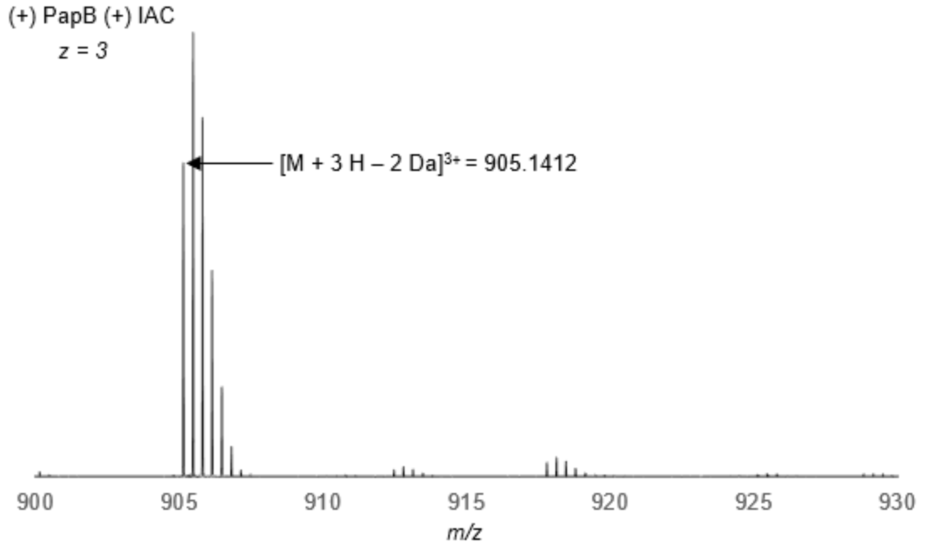

Sequence	Expected Monoisotopic Mass	Observed Monoisotopic Mass	Ppm Error
Leader-CSDNNAAA (CX_1D)	(+) PapB - (2 Da): 905.1412	905.1418	0.66
Leader-CSDNNAAA (CX_1D) + S-carboxymethyl	(-) PapB + (58.04 Da): 963.1625	Not Found	N/A

Figure S9. Iodoacetic acid treatment for CX_2D recognition sequence
Leader = LKQINVIAGVKEPIRAYG

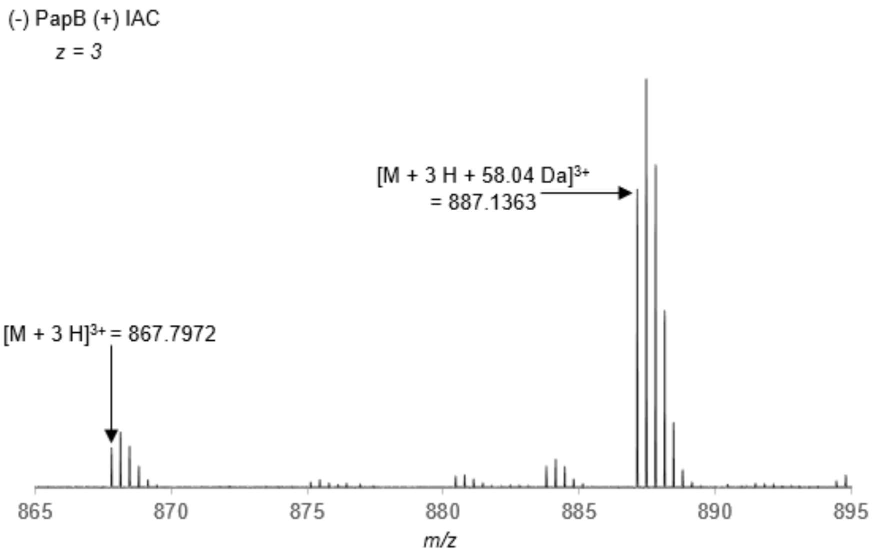

Sequence	Expected Monoisotopic Mass	Observed Monoisotopic Mass	Ppm Error
Leader-CSNDAAA (CX_2D)	(-) PapB: 867.7988	867.7972	-1.84
Leader-CSNDAAA (CX_2D) + S-carboxymethyl	(-) PapB + (58.04 Da): 887.1339	887.1363	2.71

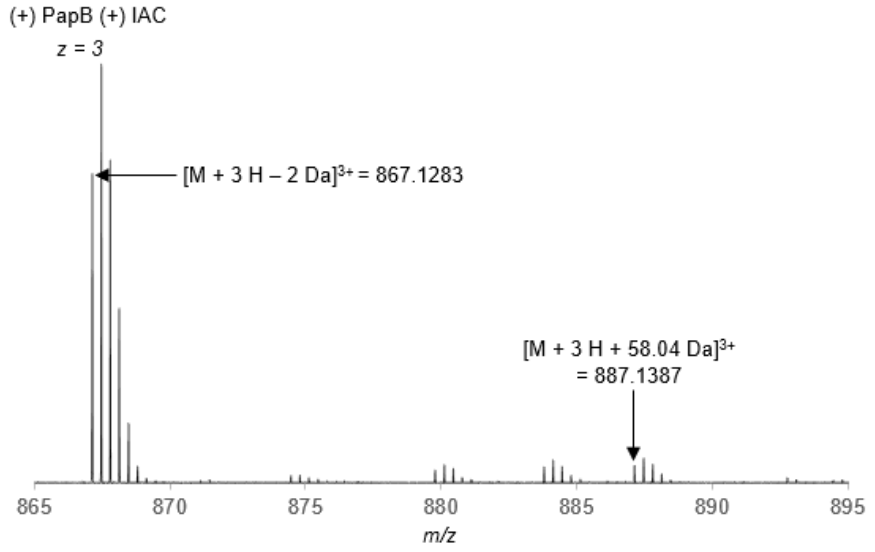

Sequence	Expected Monoisotopic Mass	Observed Monoisotopic Mass	Ppm Error
Leader-CSNDAAA (CX_2D)	(+) PapB - (2 Da): 867.1269	867.1283	1.61
Leader-CSNDAAA (CX_2D) + S-carboxymethyl	(-) PapB + (58.04 Da): 887.1339	887.1387	5.41

Figure S10. Iodoacetic acid treatment for CX_4D recognition sequence
Leader = LKQINVIAGVKEPIRAYG

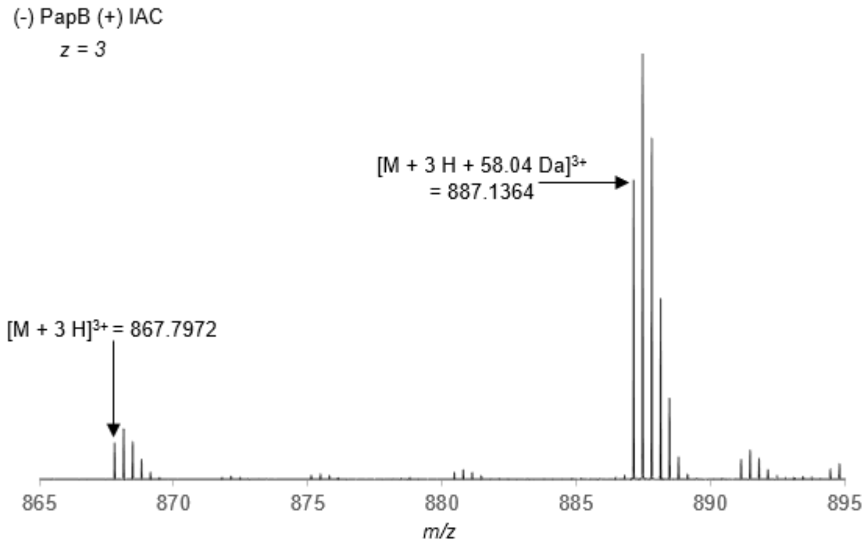

Sequence	Expected Monoisotopic Mass	Observed Monoisotopic Mass	Ppm Error
Leader-CSAANDA (CX_4D)	(-) PapB: 867.7988	867.7972	-1.84
Leader-CSAANDA (CX_4D) + S-carboxymethyl	(-) PapB + (58.04 Da): 887.1339	887.1364	2.81

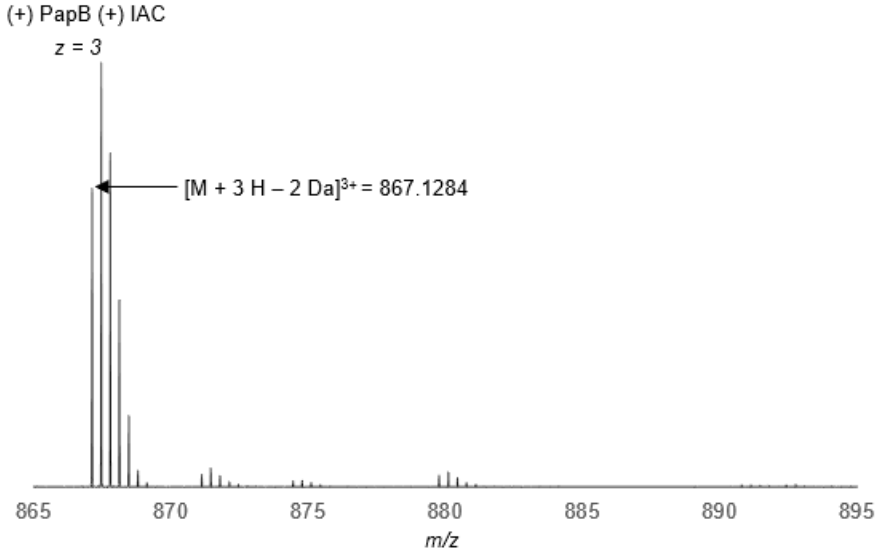

Sequence	Expected Monoisotopic Mass	Observed Monoisotopic Mass	Ppm Error
Leader-CSAANDA (CX_4D)	(+) PapB - (2 Da): 867.1269	867.1284	1.73
Leader-CSAANDA (CX_4D) + S-carboxymethyl	(-) PapB + (58.04 Da): 887.1339	Not Found	N/A

Figure S11. Iodoacetic acid treatment for CX_5D recognition sequence
Leader = LKQINVIAGVKEPIRAYG

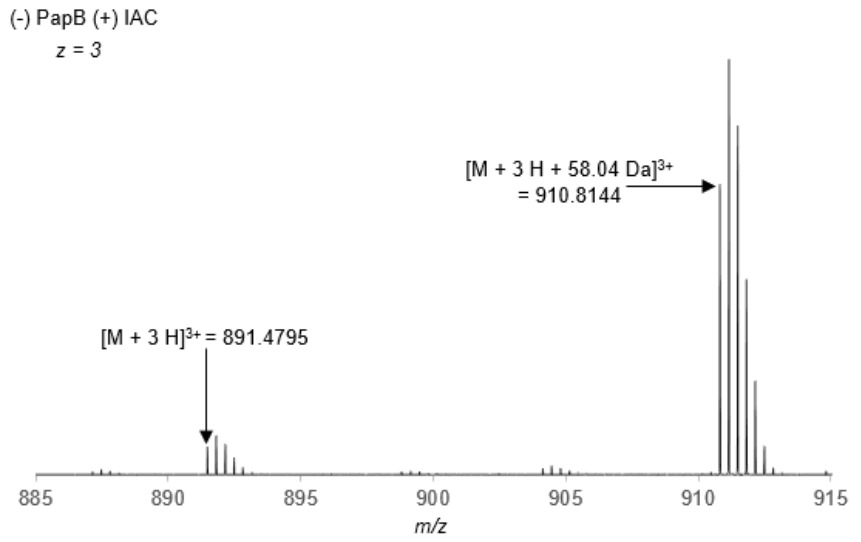

Sequence	Expected Monoisotopic Mass	Observed Monoisotopic Mass	Ppm Error
Leader-CSAAANDA (CX_5D)	(-) PapB: 891.4778	891.4795	1.91
Leader-CSAAANDA (CX_5D) + S-carboxymethyl	(-) PapB + (58.04 Da): 910.8130	910.8144	1.54

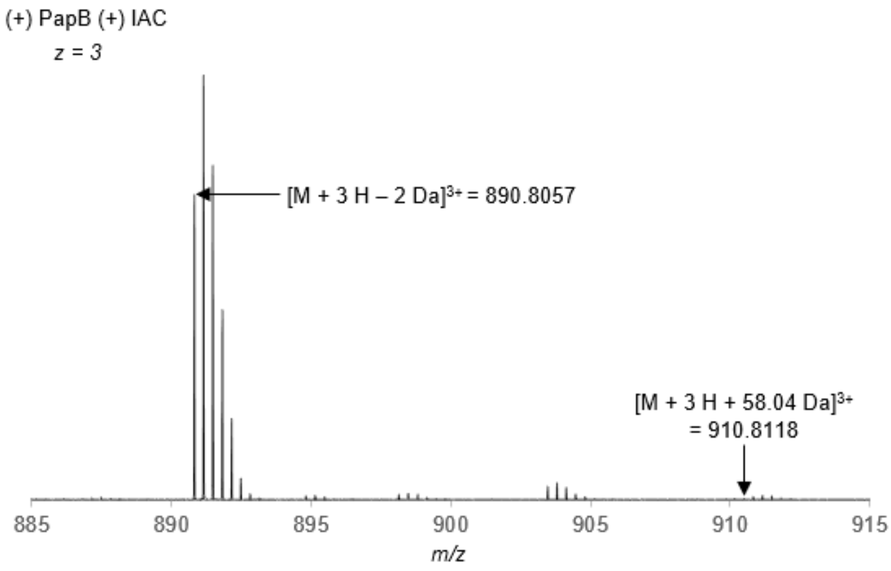

Sequence	Expected Monoisotopic Mass	Observed Monoisotopic Mass	Ppm Error
Leader-CSAAANDA (CX_5D)	(+) PapB - (2 Da): 890.8059	890.8057	-0.22
Leader-CSAAANDA (CX_5D) + S-carboxymethyl	(-) PapB + (58.04 Da): 910.8130	910.8118	-1.32

Figure S12. Iodoacetic acid treatment for CX_6D recognition sequence
Leader = LKQINVIAGVKEPIRAYG

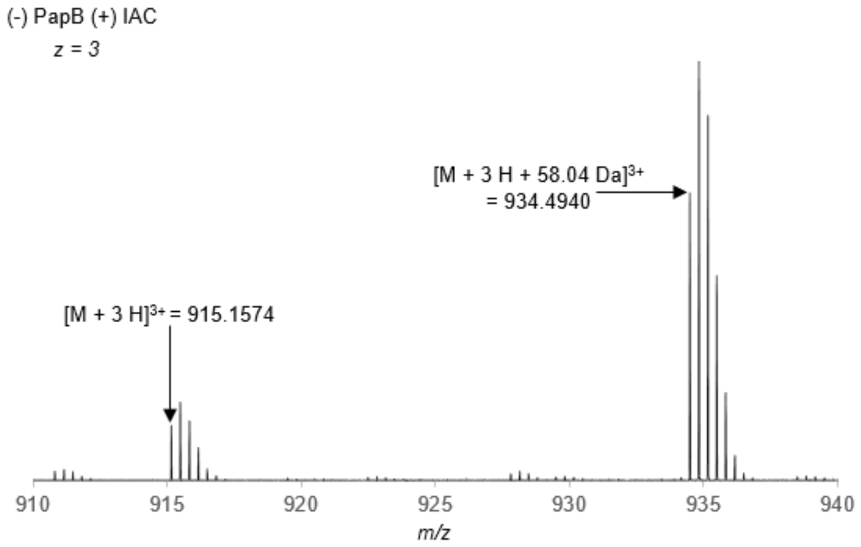

Sequence	Expected Monoisotopic Mass	Observed Monoisotopic Mass	Ppm Error
Leader-CSAAAANDA (CX_6D)	(-) PapB: 915.1568	915.1574	0.66
Leader-CSAAAANDA (CX_6D) + S-carboxymethyl	(-) PapB + (58.04 Da): 934.4920	934.4940	2.14

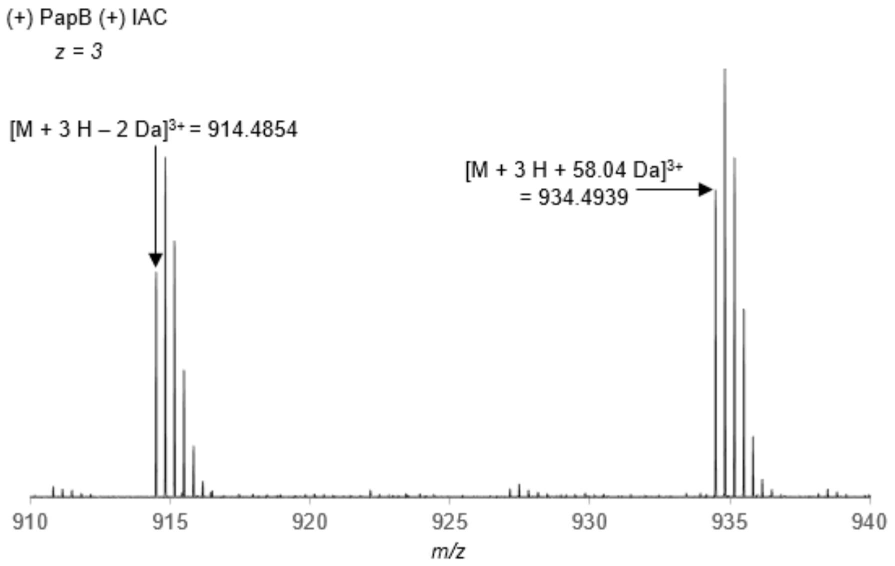

Sequence	Expected Monoisotopic Mass	Observed Monoisotopic Mass	Ppm Error
Leader-CSAAAANDA (CX_6D)	(+) PapB - (2 Da): 914.4850	914.4854	0.44
Leader-CSAAAANDA (CX_6D) + S-carboxymethyl	(-) PapB + (58.04 Da): 934.4920	934.4939	2.03

Figure S13. Tandem mass spectrometry of unmodified and modified Leader-CX_0D PeptidesSequence: LKQINVIAGVKEPIRAYG**CD**SNNAANA
(crosslinking C and D residues shown in **bold**)

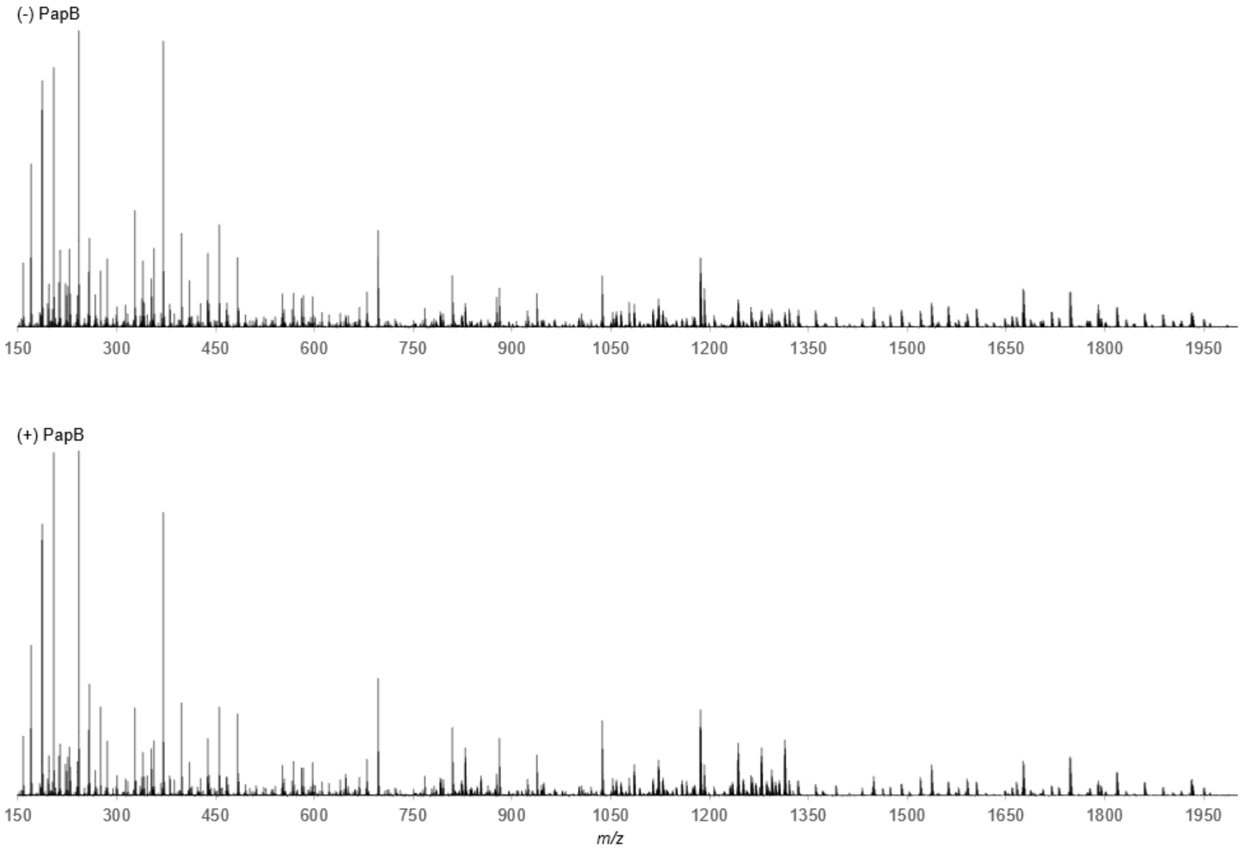

Unhighlighted indicated $z = 1$ charge state.
Gold highlight indicates $z = 2$ charge state.
Teal highlight indicates $z = 3$ charge state.
* Indicates the fragment originated from the modified peptide.

Ion	Sequence	Expected Monoisotopic Mass	Observed Monoisotopic Mass	Ppm Error
b2	LK	242.1863	242.1850	-5.36
*b2**	LK*	242.1863	242.1862	-0.43
b3	LKQ	370.2449	370.2432	-4.59
*b3**	LKQ*	370.2449	370.2450	0.27
b4	LKQI	483.3289	483.3269	-4.14
b4*	LKQI*	483.3289	483.3290	0.21
b5	LKQIN	597.3719	597.3692	-4.52
b5*	LKQIN*	597.3719	597.3708	-1.84
b6	LKQINV	696.4403	696.4371	-4.60
b6*	LKQINV*	696.4403	696.4408	0.72
b7	LKQINVI	809.5244	809.5222	-2.72
b7*	LKQINVI*	809.5244	809.5245	0.12

b8	LKQINVIA	880.5615	880.5570	-5.11
b8*	LKQINVIA*	880.5615	880.5612	-0.34
b9	LKQINVIAG	937.5829	937.5833	0.43
b9*	LKQINVIAG*	937.5829	937.5838	0.96
b10	LKQINVIAGV	1036.6513	1036.6462	-4.92
b10*	LKQINVIAGV*	1036.6513	1036.6520	0.67
b11	LKQINVIAGVK	1164.7463	1164.7429	-2.92
b11*	LKQINVIAGVK*	1164.7463	1164.7484	1.80
b12	LKQINVIAGVKE	1293.7889	1293.7865	-1.86
b12*	LKQINVIAGVKE*	1293.7889	1293.7904	1.16
b13	LKQINVIAGVKEP	1390.8417	Not Found	N/A
b13*	LKQINVIAGVKEP*	1390.8417	1390.8349	-4.89
b14	LKQINVIAGVKEPI	1503.9257	1503.9224	-2.19
b14*	LKQINVIAGVKEPI*	1503.9257	1503.9261	0.27
b15	LKQINVIAGVKEPIR	1660.0268	1660.0205	-3.79
b15*	LKQINVIAGVKEPIR*	1660.0268	1660.0196	-4.33
b16	LKQINVIAGVKEPIRA	1731.0640	1731.0599	-2.36
b16*	LKQINVIAGVKEPIRA*	1731.0640	1731.0645	0.29
b17	LKQINVIAGVKEPIRAY	1894.1273	Not Found	N/A
b17*	LKQINVIAGVKEPIRAY*	1894.1273	Not Found	N/A
b18	LKQINVIAGVKEPIRAYG	976.0780	976.0725	-5.63
b18*	LKQINVIAGVKEPIRAYG*	976.0780	976.0777	-0.31
b19	LKQINVIAGVKEPIRAYGC	1027.5826	1027.5796	-2.92
b19*	(-1 Da) LKQINVIAGVKEPIRAYG**C***	1027.0787	Not Found	N/A
b20	LKQINVIAGVKEPIRAYGCD	1085.0961	1085.0921	-3.69
b20*	(-2 Da) LKQINVIAGVKEPIRAYG**CD***	1084.0882	1084.0874	-0.74
b21	LKQINVIAGVKEPIRAYGCDS	1128.6121	1128.6078	-3.81
b21*	(-2 Da) LKQINVIAGVKEPIRAYG**CD**S*	1127.6043	1127.6063	1.77
b22	LKQINVIAGVKEPIRAYGCDSN	1185.6336	1185.6302	-2.87
b22*	(-2 Da) LKQINVIAGVKEPIRAYG**CD**SN*	1184.6257	1184.6283	2.19
b23	LKQINVIAGVKEPIRAYGCDSNN	828.7724	828.7695	-3.50
b23*	(-2 Da) LKQINVIAGVKEPIRAYG**CD**SNN*	828.1006	828.1005	-0.12
b24	LKQINVIAGVKEPIRAYGCDSNNA	852.4515	852.4478	-4.34
b24*	(-2 Da) LKQINVIAGVKEPIRAYG**CD**SNNA*	851.7796	851.7794	-0.23
b25	LKQINVIAGVKEPIRAYGCDSNNAA	876.1305	876.1286	-2.17
b25*	(-2 Da) LKQINVIAGVKEPIRAYG**CD**SNNAA*	875.4586	Not Found	N/A
b26	LKQINVIAGVKEPIRAYGCDSNNAAN	914.1448	914.1410	-4.16
b26*	(-2 Da) LKQINVIAGVKEPIRAYG**CD**SNNAAN*	913.4729	913.4714	-1.64
[M]	LKQINVIAGVKEPIRAYGCDSNNAANA	943.8274	943.8234	-4.24
[M]*	(-2Da)LKQINVIAGVKEPIRAYG**CD**SNNAANA*	943.1555	943.1543	-1.27
y26	KQINVIAGVKEPIRAYGCDSNNAANA	906.1327	906.1325	-0.22
y26*	(-2 Da) KQINVIAGVKEPIRAYG**CD**SNNAANA*	905.4608	905.4659	5.63
y25	QINVIAGVKEPIRAYGCDSNNAANA	863.4344	863.4344	0.00
y25*	(-2 Da) QINVIAGVKEPIRAYG**CD**SNNAANA*	862.7625	862.7652	3.13
y24	INVIAGVKEPIRAYGCDSNNAANA	820.7482	Not Found	N/A
y24*	(-2 Da) INVIAGVKEPIRAYG**CD**SNNAANA*	820.0763	Not Found	N/A
y23	NVIAGVKEPIRAYGCDSNNAANA	783.0535	783.0503	-4.08
y23*	(-2 Da) NVIAGVKEPIRAYG**CD**SNNAANA*	782.3816	782.3773	-5.50
y22	VIAGVKEPIRAYGCDSNNAANA	1117.0551	1117.0495	-5.01
y22*	(-2 Da) VIAGVKEPIRAYG**CD**SNNAANA*	1116.0473	1116.0499	2.33

y21<br>y21*	IAGVKEPIRAYGCDSNNAANA<br>(-2 Da) IAGVKEPIRAYG**CD**SNNAANA*	1067.5209<br>1066.5131	1067.5218<br>Not Found	0.84<br>N/A
y20<br>y20*	AGVKEPIRAYGCDSNNAANA<br>(-2 Da) AGVKEPIRAYG**CD**SNNAANA*	1010.9789<br>1009.9711	1010.9735<br>1009.9712	-5.34<br>0.10
y19<br>y19*	GVKEPIRAYGCDSNNAANA<br>(-2 Da) GVKEPIRAYG**CD**SNNAANA*	975.4603<br>974.4525	975.4565<br>974.4523	-3.90<br>-0.20
y18<br>y18*	VKEPIRAYGCDSNNAANA<br>(-2 Da) VKEPIRAYG**CD**SNNAANA*	946.9496<br>945.9418	Not Found<br>Not Found	N/A<br>N/A
y17<br>y17*	KEPIRAYGCDSNNAANA<br>(-2 Da) KEPIRAYG**CD**SNNAANA*	1793.8235<br>1791.8079	1793.8133<br>Not Found	-5.69<br>N/A
y16<br>y16*	EPIRAYGCDSNNAANA<br>(-2 Da) EPIRAYG**CD**SNNAANA*	1665.7286<br>1663.7129	1665.7236<br>1663.7163	-3.00<br>2.04
y15<br>y15*	PIRAYGCDSNNAANA<br>(-2 Da) PIRAYG**CD**SNNAANA*	1536.6860<br>1534.6703	1536.6802<br>1534.6654	-3.77<br>-3.19
y14<br>y14*	IRAYGCDSNNAANA<br>(-2 Da) IRAYG**CD**SNNAANA*	1439.6332<br>1437.6176	Not Found<br>Not Found	N/A<br>N/A
y13<br>y13*	RAYGCDSNNAANA<br>(-2 Da) RAYG**CD**SNNAANA*	1326.5491<br>1324.5335	1326.5424<br>Not Found	-5.05<br>N/A
y12<br>y12*	AYGCDSNNAANA<br>(-2 Da) AYG**CD**SNNAANA*	1170.4480<br>1168.4324	1170.4422<br>Not Found	-4.09<br>N/A
y11<br>y11*	YGCDSNNAANA<br>(-2 Da) YG**CD**SNNAANA*	1099.4109<br>1097.3953	1099.4099<br>1097.3949	-0.91<br>-0.36
y10<br>y10*	GCDSNNAANA<br>(-2 Da) G**CD**SNNAANA*	936.3476<br>934.3319	936.3507<br>934.3333	3.31<br>1.50
y9<br>y9*	CDSNNAANA<br>(-2 Da) **CD**SNNAANA*	879.3261<br>877.3105	Not Found<br>Not Found	N/A<br>N/A
y8<br>y8*	DSNNAANA<br>(-1 Da) **D**SNNAANA*	776.3169<br>775.3091	776.3141<br>Not Found	-3.61<br>N/A
y7<br>y7*	SNNAANA<br>SNNAANA*	661.2900<br>661.2900	661.2875<br>661.2896	-3.78<br>-0.60
y6<br>y6*	NNAANA<br>NNAANA*	574.2580<br>574.2580	574.2559<br>574.2578	-3.65<br>-0.35
y5<br>y5*	NAANA<br>NAANA*	460.2150<br>460.2150	460.2127<br>460.2151	-5.00<br>0.22
y4<br>y4*	AANA<br>AANA*	346.1721<br>346.1721	346.1704<br>346.1722	-4.91<br>0.29
y3<br>y3*	ANA<br>ANA*	275.1350<br>275.1350	275.1340<br>275.1349	-3.63<br>-0.36
y2<br>y2*	NA<br>NA*	204.0979<br>204.0979	204.0967<br>204.0980	-5.87<br>0.49

Figure S14. Tandem mass spectrometry of unmodified and modified Leader-CX$_1$D Peptides

Sequence: LKQINVIAGVKEPIRAYG**C**S**D**NNAAA
(crosslinking C and D residues shown in **bold**)

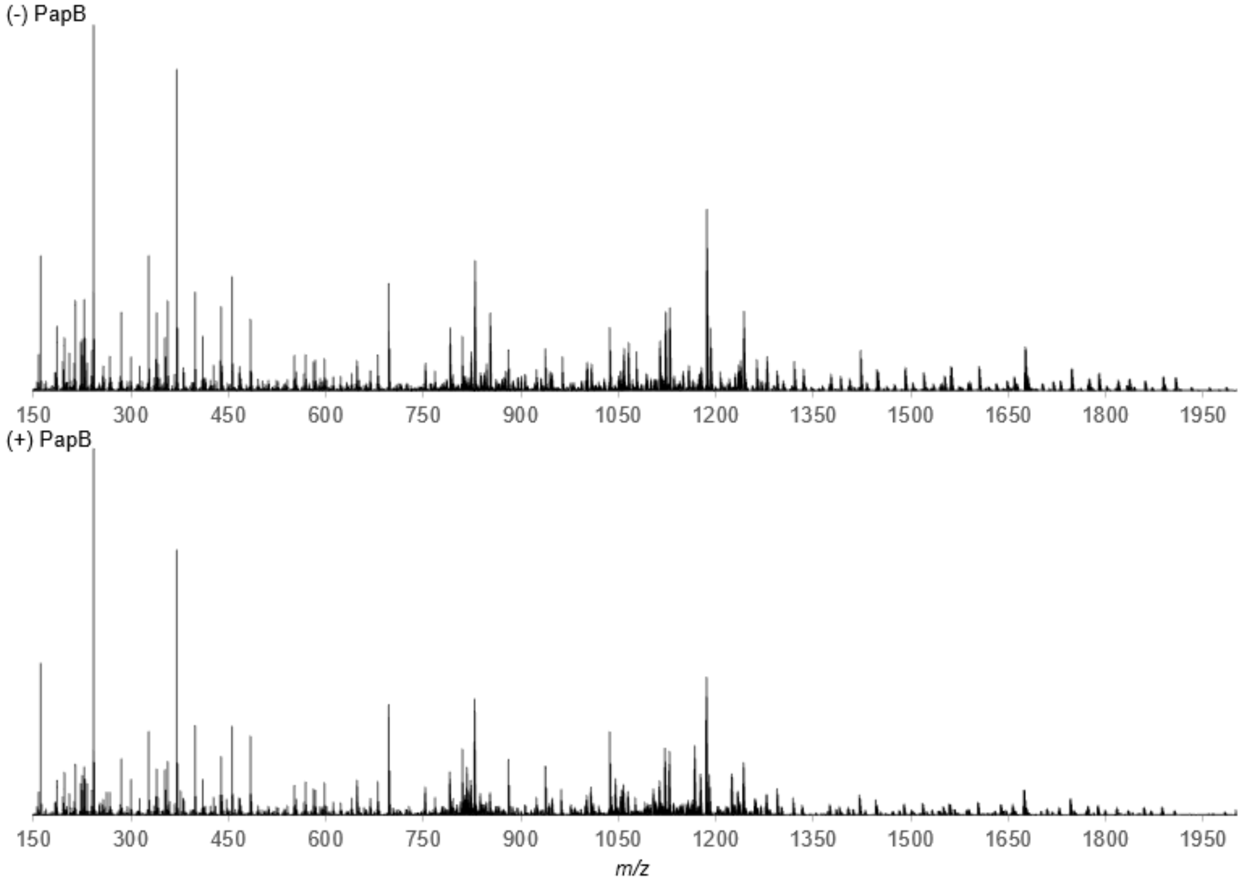

Unhighlighted indicates *z* = 1 charge state.
Gold highlight indicates *z* = 2 charge state.
Teal highlight indicates *z* = 3 charge state.
* Indicates the fragment originated from the modified peptide.

Ion	Sequence	Expected Monoisotopic Mass	Observed Monoisotopic Mass	Ppm Error
b2	LK	242.1863	242.1859	-1.65
*b2**	LK*	242.1863	242.1864	0.41
b3	LKQ	370.2449	370.2442	-1.89
*b3**	LKQ*	370.2449	370.2454	1.35
b4	LKQI	483.3289	483.3268	-4.34
b4*	LKQI*	483.3289	483.3294	1.03
b5	LKQIN	597.3719	597.3690	-4.85
b5*	LKQIN*	597.3719	597.3723	0.670
b6	LKQINV	696.4403	696.4369	-4.88
b6*	LKQINV*	696.4403	696.4411	1.15

b7<br>b7*	LKQINVI<br>LKQINVI*	809.5244<br>809.5244	809.5213<br>809.5250	-3.83<br>0.74
b8<br>b8*	LKQINVIA<br>LKQINVIA*	880.5615<br>880.5615	880.5576<br>880.5621	-4.43<br>0.68
b9<br>b9*	LKQINVIAG<br>LKQINVIAG*	937.5829<br>937.5829	937.5807<br>937.5834	-2.35<br>0.53
b10<br>b10*	LKQINVIAGV<br>LKQINVIAGV*	1036.6513<br>1036.6513	1036.6462<br>1036.6518	-4.92<br>0.48
b11<br>b11*	LKQINVIAGVK<br>LKQINVIAGVK*	1164.7463<br>1164.7463	1164.7416<br>1164.7469	-4.04<br>0.52
b12<br>b12*	LKQINVIAGVKE<br>LKQINVIAGVKE*	1293.7889<br>1293.7889	1293.7892<br>1293.7904	0.23<br>1.15
b13<br>b13*	LKQINVIAGVKEP<br>LKQINVIAGVKEP*	1390.8417<br>1390.8417	Not Found<br>1390.8405	N/A<br>-0.86
b14<br>b14*	LKQINVIAGVKEPI<br>LKQINVIAGVKEPI*	1503.9257<br>1503.9257	1503.9269<br>1503.9231	0.80<br>-1.72
b15<br>b15*	LKQINVIAGVKEPIR<br>LKQINVIAGVKEPIR*	1660.0268<br>1660.0268	1660.0259<br>1660.0241	-0.54<br>-1.63
b16<br>b16*	LKQINVIAGVKEPIRA<br>LKQINVIAGVKEPIRA*	1731.0640<br>1731.0640	1731.0672<br>1731.0588	1.85<br>-3.00
b17<br>b17*	LKQINVIAGVKEPIRAY<br>LKQINVIAGVKEPIRAY*	1894.1273<br>1894.1273	1894.1249<br>Not Found	-1.26<br>N/A
b18<br>b18*	LKQINVIAGVKEPIRAYG<br>LKQINVIAGVKEPIRAYG*	976.0780<br>976.0780	976.0740<br>976.0784	-4.09<br>0.41
b19<br>b19*	LKQINVIAGVKEPIRAYGC<br>(-1 Da) LKQINVIAGVKEPIRAYG**C***	1027.5826<br>1027.0787	1027.5809<br>Not Found	-1.65<br>N/A
b20<br>b20*	LKQINVIAGVKEPIRAYGCS<br>(-2 Da) LKQINVIAGVKEPIRAYG**C**S*	1071.0986<br>1070.5947	1071.0959<br>Not Found	-2.52<br>N/A
b21<br>b21*	LKQINVIAGVKEPIRAYGCSD<br>(-2 Da) LKQINVIAGVKEPIRAYG**C**S**D***	1128.6121<br>1127.6043	1128.6072<br>1127.6062	-4.34<br>1.68
b22<br>b22*	LKQINVIAGVKEPIRAYGCSDN<br>(-2 Da) LKQINVIAGVKEPIRAYG**C**S**D**N*	1185.6336<br>1184.6257	1185.6282<br>1184.6277	-4.55<br>1.69
b23<br>b23*	LKQINVIAGVKEPIRAYGCSDNN<br>(-2 Da) LKQINVIAGVKEPIRAYG**C**S**D**NN*	828.7724<br>828.1006	828.7675<br>828.1010	-5.91<br>0.48
b24<br>b24*	LKQINVIAGVKEPIRAYGCSDNNA<br>(-2 Da) LKQINVIAGVKEPIRAYG**C**S**D**NNA*	852.4515<br>851.7796	852.4475<br>851.7798	-4.69<br>0.23
b25<br>b25*	LKQINVIAGVKEPIRAYGCSDNNAA<br>(-2 Da) LKQINVIAGVKEPIRAYG**C**S**D**NNAA*	876.1305<br>875.4586	876.1295<br>875.4587	-1.14<br>0.11
[M]<br>[M]*	LKQINVIAGVKEPIRAYGCSDNNAAA<br>(-2 Da) LKQINVIAGVKEPIRAYG**C**S**D**NNAAA*	905.8131<br>905.1412	905.8138<br>905.1410	0.77<br>-0.22
y25<br>y25*	KQINVIAGVKEPIRAYGCSDNNAAA<br>(-2 Da) KQINVIAGVKEPIRAYG**C**S**D**NNAAA*	868.1184<br>867.4465	868.1194<br>Not Found	1.15<br>N/A
y24<br>y24*	QINVIAGVKEPIRAYGCSDNNAAA<br>(-2 Da) QINVIAGVKEPIRAYG**C**S**D**NNAAA*	825.4201<br>824.7482	Not Found<br>Not Found	N/A<br>N/A
y23<br>y23*	INVIAGVKEPIRAYGCSDNNAAA<br>(-2 Da) INVIAGVKEPIRAYG**C**S**D**NNAAA*	782.7339<br>782.0620	Not Found<br>Not Found	N/A<br>N/A
y22<br>y22*	NVIAGVKEPIRAYGCSDNNAAA<br>(-2 Da) NVIAGVKEPIRAYG**C**S**D**NNAAA*	745.0392<br>744.3673	745.0391<br>Not Found	-0.13<br>N/A
y21<br>y21*	VIAGVKEPIRAYGCSDNNAAA<br>(-2 Da) VIAGVKEPIRAYG**C**S**D**NNAAA*	1060.0337<br>1059.0258	1060.0328<br>1059.0323	-0.85<br>6.13

y20<br>y20*	IAGVKEPIRAYGCSDNNAAA<br>(-2 Da) IAGVKEPIRAYG**C**S**D**NNAAA*	1010.4995<br>1009.4916	1010.4992<br>1009.4903	-0.30<br>-1.28
y19<br>y19*	AGVKEPIRAYGCSDNNAAA<br>(-2 Da) AGVKEPIRAYG**C**S**D**NNAAA*	953.9574<br>952.9496	953.9570<br>952.9494	-0.42<br>-0.21
y18<br>y18*	GVKEPIRAYGCSDNNAAA<br>(-2 Da) GVKEPIRAYG**C**S**D**NNAAA*	918.4389<br>917.4310	918.4353<br>917.4311	-3.92<br>0.11
y17<br>y17*	VKEPIRAYGCSDNNAAA<br>(-2 Da) VKEPIRAYG**C**S**D**NNAAA*	889.9281<br>888.9203	889.9283<br>888.9226	0.22<br>2.59
y16<br>y16*	KEPIRAYGCSDNNAAA<br>(-2 Da) KEPIRAYG**C**S**D**NNAAA*	1679.7806<br>1677.7649	1679.7807<br>1677.7671	0.06<br>1.31
y15<br>y15*	EPIRAYGCSDNNAAA<br>(-2 Da) EPIRAYG**C**S**D**NNAAA*	1551.6856<br>1549.6700	1551.6816<br>1549.6706	-2.58<br>0.38
y14<br>y14*	PIRAYGCSDNNAAA<br>(-2 Da) PIRAYG**C**S**D**NNAAA*	1422.6430<br>1420.6274	1422.6373<br>1420.6277	-4.01<br>0.21
y13<br>y13*	IRAYGCSDNNAAA<br>(-2 Da) IRAYG**C**S**D**NNAAA*	1325.5903<br>1323.5746	1325.5886<br>1323.5736	-1.28<br>-0.75
y12<br>y12*	RAYGCSDNNAAA<br>(-2 Da) RAYG**C**S**D**NNAAA*	1212.5062<br>1210.4906	1212.5059<br>1210.4893	-0.25<br>-1.07
y11<br>y11*	AYGCSDNNAAA<br>(-2 Da) AYG**C**S**D**NNAAA*	1056.4051<br>1054.3894	Not Found<br>1054.3885	N/A<br>-0.85
y10<br>y10*	YGCSDNNAAA<br>(-2 Da) YG**C**S**D**NNAAA*	985.3680<br>983.3523	985.3618<br>983.3513	-6.29<br>-1.02
y9<br>y9*	GCSDNNAAA<br>(-2 Da) G**C**S**D**NNAAA*	822.3047<br>820.2890	822.3010<br>820.2887	-4.50<br>-0.37
y8<br>y8*	CSDNNAAA<br>(-2 Da) **C**S**D**NNAAA*	765.2832<br>763.2675	765.2798<br>763.2687	-4.44<br>1.57
y7<br>y7*	SDNNAAA<br>(-1 Da) S**D**NNAAA*	662.2740<br>661.2662	662.2699<br>Not Found	6.19<br>N/A
y6<br>y6*	DNNAAA<br>(-1 Da) **D**NNAAA*	575.2420<br>574.2342	575.2402<br>Not Found	-3.13<br>N/A
y5<br>y5*	NNAAA<br>NNAAA*	460.2150<br>460.2150	460.2139<br>460.2154	-2.39<br>0.87
y4<br>y4*	NAAA<br>NAAA*	346.1721<br>346.1721	346.1704<br>346.1724	-4.91<br>0.87
y3<br>y3*	AAA<br>AAA*	232.1292<br>232.1292	232.1289<br>232.1293	-1.29<br>0.43
y2<br>y2*	AA<br>AA*	161.0921<br>161.0921	161.0916<br>161.0921	-3.10<br>0

Figure S15. Tandem mass spectrometry of unmodified and modified Leader-CX$_2$D PeptidesSequence: LKQINVIAGVKEPIRAYG**C**SN**D**AAA
(crosslinking C and D residues shown in **bold**)

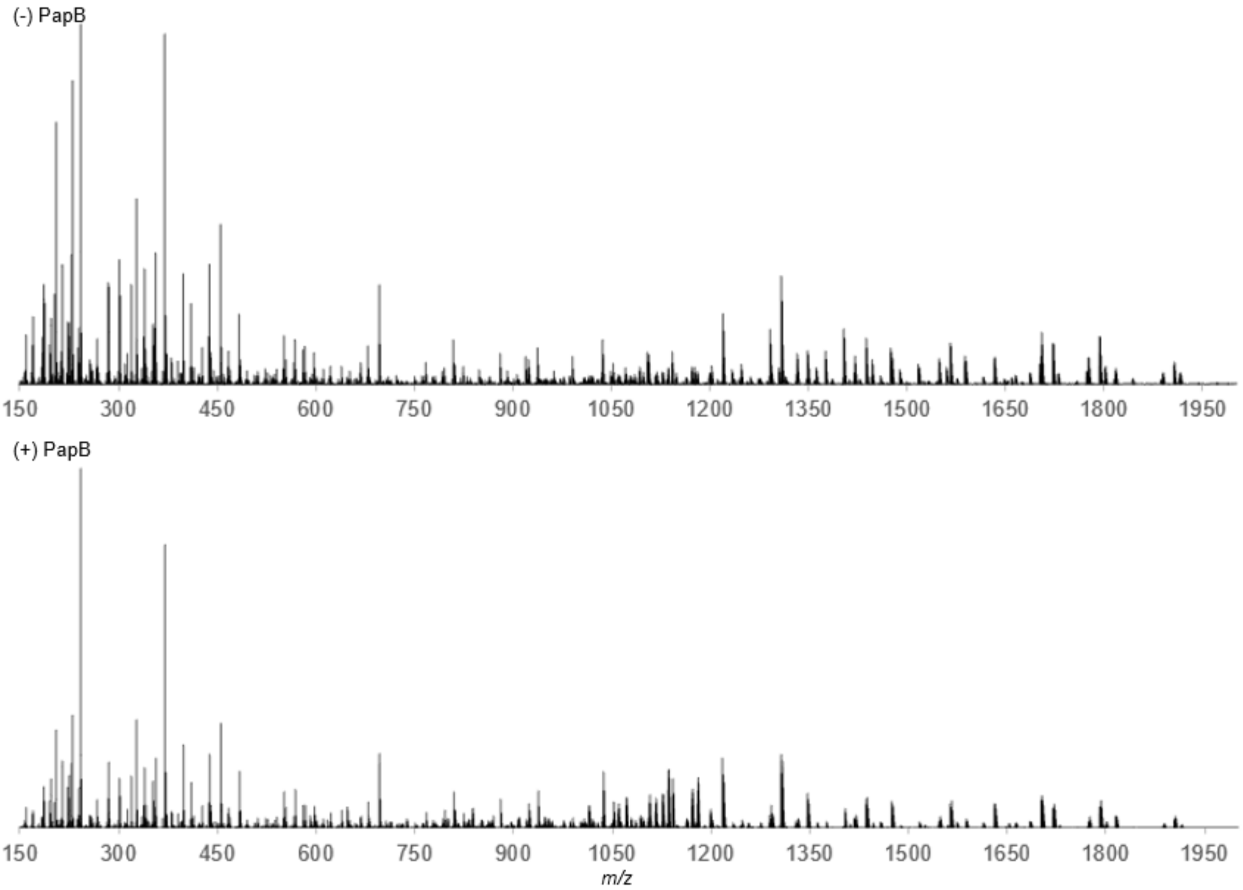

Gold highlight indicates *z* = 2 charge state.
Teal highlight indicates *z* = 3 charge state.
* Indicates the fragment originated from the modified peptide.

Ion	Sequence	Expected Monoisotopic Mass	Observed Monoisotopic Mass	Ppm Error
b2	LK	242.1863	242.1850	-5.36
*b2**	LK*	242.1863	242.1862	-0.41
b3	LKQ	370.2449	370.2432	-4.59
*b3**	LKQ*	370.2449	370.2450	0.27
b4	LKQI	483.3289	483.3269	-4.13
b4*	LKQI*	483.3289	483.3290	0.20
b5	LKQIN	597.3719	597.3692	-4.51
b5*	LKQIN*	597.3719	597.3708	-1.84
b6	LKQINV	696.4403	696.4371	-4.59
b6*	LKQINV*	696.4403	696.4408	0.71
b7	LKQINVI	809.5244	809.5222	-2.71
b7*	LKQINVI*	809.5244	809.5245	0.12
b8	LKQINVIA	880.5615	880.5580	-3.97

b8*	LKQINVIA*	880.5615	880.5612	-0.34
b9<br>b9*	LKQINVIAG<br>LKQINVIAG*	937.5829<br>937.5829	937.5833<br>937.5838	0.42<br>0.95
b10<br>b10*	LKQINVIAGV<br>LKQINVIAGV*	1036.6513<br>1036.6513	1036.6472<br>1036.6520	-3.95<br>0.67
b11<br>b11*	LKQINVIAGVK<br>LKQINVIAGVK*	1164.7463<br>1164.7463	1164.7429<br>1164.7484	-2.91<br>1.80
b12<br>b12*	LKQINVIAGVKE<br>LKQINVIAGVKE*	1293.7889<br>1293.7889	1293.7865<br>1293.7904	-1.85<br>1.16
b13<br>b13*	LKQINVIAGVKEP<br>LKQINVIAGVKEP*	1390.8417<br>1390.8417	Not Found<br>1390.8349	N/A<br>-4.88
b14<br>b14*	LKQINVIAGVKEPI<br>LKQINVIAGVKEPI*	1503.9257<br>1503.9257	1503.9224<br>1503.9261	-2.19<br>0.26
b15<br>b15*	LKQINVIAGVKEPIR<br>LKQINVIAGVKEPIR*	1660.0268<br>1660.0268	1660.0205<br>1660.0196	-3.79<br>-4.33
b16<br>b16*	LKQINVIAGVKEPIRA<br>LKQINVIAGVKEPIRA*	1731.0640<br>1731.0640	1731.0645<br>1731.0599	0.28<br>-2.36
b17<br>b17*	LKQINVIAGVKEPIRAY<br>LKQINVIAGVKEPIRAY*	1894.1273<br>1894.1273	Not Found<br>Not Found	N/A<br>N/A
b18<br>b18*	LKQINVIAGVKEPIRAYG<br>LKQINVIAGVKEPIRAYG*	976.0780<br>976.0780	976.0755<br>976.0777	-2.56<br>-0.30
b19<br>b19*	LKQINVIAGVKEPIRAYGC<br>(-1 Da) LKQINVIAGVKEPIRAYG**C***	1027.5826<br>1027.0787	1027.5796<br>Not Found	-2.91<br>N/A
b20<br>b20*	LKQINVIAGVKEPIRAYGCS<br>(-1 Da) LKQINVIAGVKEPIRAYG**C**S*	1071.5626<br>1070.5947	1071.5614<br>Not Found	-1.11<br>N/A
b21<br>b21*	LKQINVIAGVKEPIRAYGCSN<br>(-1 Da) LKQINVIAGVKEPIRAYG**C**SN*	1128.6121<br>1127.6043	1128.6162<br>Not Found	3.63<br>N/A
b22<br>b22*	LKQINVIAGVKEPIRAYGCSND<br>(-2 Da) LKQINVIAGVKEPIRAYG**C**SN**D***	1185.6336<br>1184.6257	1185.6344<br>1184.6242	0.67<br>-1.26
b23<br>b23*	LKQINVIAGVKEPIRAYGCSNDA<br>(-2 Da) LKQINVIAGVKEPIRAYG**C**SN**D**A*	814.4372<br>813.7653	814.4347<br>813.7661	-3.07<br>0.98
b24<br>b24*	LKQINVIAGVKEPIRAYGCSNDAA<br>(-2 Da) LKQINVIAGVKEPIRAYG**C**SN**D**AA*	838.1162<br>837.4443	838.1121<br>837.4445	-4.89<br>0.24
[M]<br>[M]*	LKQINVIAGVKEPIRAYGCSNDAAA<br>(-2 Da) LKQINVIAGVKEPIRAYG**C**SN**D**AAA*	867.7988<br>867.1269	867.7947<br>867.1268	-4.72<br>-0.11
y24<br>y24*	KQINVIAGVKEPIRAYGCSNDAAA<br>(-2 Da) KQINVIAGVKEPIRAYG**C**SN**D**AAA*	830.1041<br>829.4322	830.1020<br>829.4326	-2.53<br>0.48
y23<br>y23*	QINVIAGVKEPIRAYGCSNDAAA<br>(-2 Da) QINVIAGVKEPIRAYG**C**SN**D**AAA*	787.4058<br>786.7339	787.4063<br>786.7335	0.63<br>-0.51
y22<br>y22*	INVIAGVKEPIRAYGCSNDAAA<br>(-2 Da) INVIAGVKEPIRAYG**C**SN**D**AAA*	1116.5757<br>1115.5679	1116.5719<br>1115.5670	-3.40<br>-0.81
y21<br>y21*	NVIAGVKEPIRAYGCSNDAAA<br>(-2 Da) NVIAGVKEPIRAYG**C**SN**D**AAA*	1060.0377<br>1059.0258	1060.0333<br>1059.0260	-4.15<br>0.19
y20<br>y20*	VIAGVKEPIRAYGCSNDAAA<br>(-2 Da) VIAGVKEPIRAYG**C**SN**D**AAA*	1003.0122<br>1002.0044	1003.0079<br>1002.0047	-4.29<br>0.30
y19<br>y19*	IAGVKEPIRAYGCSNDAAA<br>(-2 Da) IAGVKEPIRAYG**C**SN**D**AAA*	953.4780<br>952.4702	953.4733<br>952.4701	-4.93<br>-0.10
y18<br>y18*	AGVKEPIRAYGCSNDAAA<br>(-2 Da) AGVKEPIRAYG**C**SN**D**AAA*	896.9360<br>895.9281	896.9361<br>895.9276	0.11<br>-0.56

y17<br>y17*	GVKEPIRAYGCSNDAAA<br>(-2 Da) GVKEPIRAYG**C**SN**D**AAA*	1721.8275<br>1719.8119	1721.8199<br>1719.8161	-4.41<br>2.44
y16<br>y16*	VKEPIRAYGCSNDAAA<br>(-2 Da) VKEPIRAYG**C**SN**D**AAA*	1664.8061<br>1662.7904	1664.8049<br>1662.7873	-0.72<br>-1.86
y15<br>y15*	KEPIRAYGCSNDAAA<br>(-2 Da) KEPIRAYG**C**SN**D**AAA*	1565.7377<br>1563.6271	1565.7379<br>1563.6308	0.13<br>2.37
y14<br>y14*	EPIRAYGCSNDAAA<br>(-2 Da) EPIRAYG**C**SN**D**AAA*	1437.6427<br>1435.6271	1437.6395<br>1435.6292	-2.22<br>1.47
y13<br>y13*	PIRAYGCSNDAAA<br>(-2 Da) PIRAYG**C**SN**D**AAA*	1308.6001<br>1306.5845	1308.5982<br>1306.5869	-1.45<br>1.84
y12<br>y12*	IRAYGCSNDAAA<br>(-2 Da) IRAYG**C**SN**D**AAA*	1211.5473<br>1209.5317	1211.5478<br>1209.5339	0.41<br>1.82
y11<br>y11*	RAYGCSNDAAA<br>(-2 Da) RAYG**C**SN**D**AAA*	1098.4633<br>1096.4476	1098.4599<br>1096.4490	-3.09<br>1.28
y10<br>y10*	AYGCSNDAAA<br>(-2 Da) AYG**C**SN**D**AAA*	942.3622<br>940.3465	942.3587<br>940.3490	-3.71<br>2.66
y9<br>y9*	YGCSNDAAA<br>(-2 Da) YG**C**SN**D**AAA*	871.3251<br>869.3094	871.3208<br>869.3094	-4.94<br>0
y8<br>y8*	GCSNDAAA<br>(-2 Da) G**C**SN**D**AAA*	708.2617<br>706.2461	708.2585<br>706.2460	-4.52<br>0.14
y7<br>y7*	CSNDAAA<br>(-2 Da)**C**SN**D**AAA*	651.2403<br>649.2246	651.2374<br>649.2247	-4.45<br>0.15
y6<br>y6*	SNDAAA<br>(-1 Da) SN**D**AAA*	548.2311<br>547.2233	548.2296<br>Not Found	-2.73<br>N/A
y5<br>y5*	NDAAA<br>(-1 Da) N**D**AAA*	461.1991<br>460.1921	461.1986<br>Not Found	-1.08<br>N/A
y4<br>y4*	DAAA<br>(-1 Da) **D**AAA*	347.1561<br>346.1483	347.1554<br>Not Found	-2.02<br>N/A
y3<br>y3*	AAA<br>AAA*	232.1292<br>232.1292	232.1281<br>232.1294	-4.73<br>0.86
y2<br>y2*	AA<br>AA*	161.0921<br>161.0921	161.0911<br>161.0919	-6.20<br>-1.24

Figure S16. Tandem mass spectrometry of unmodified and modified Leader-CX$_4$D Peptides
Sequence: LKQINVIAGVKEPIRAYG**C**SAAN**D**A
(crosslinking C and D residues shown in **bold**)

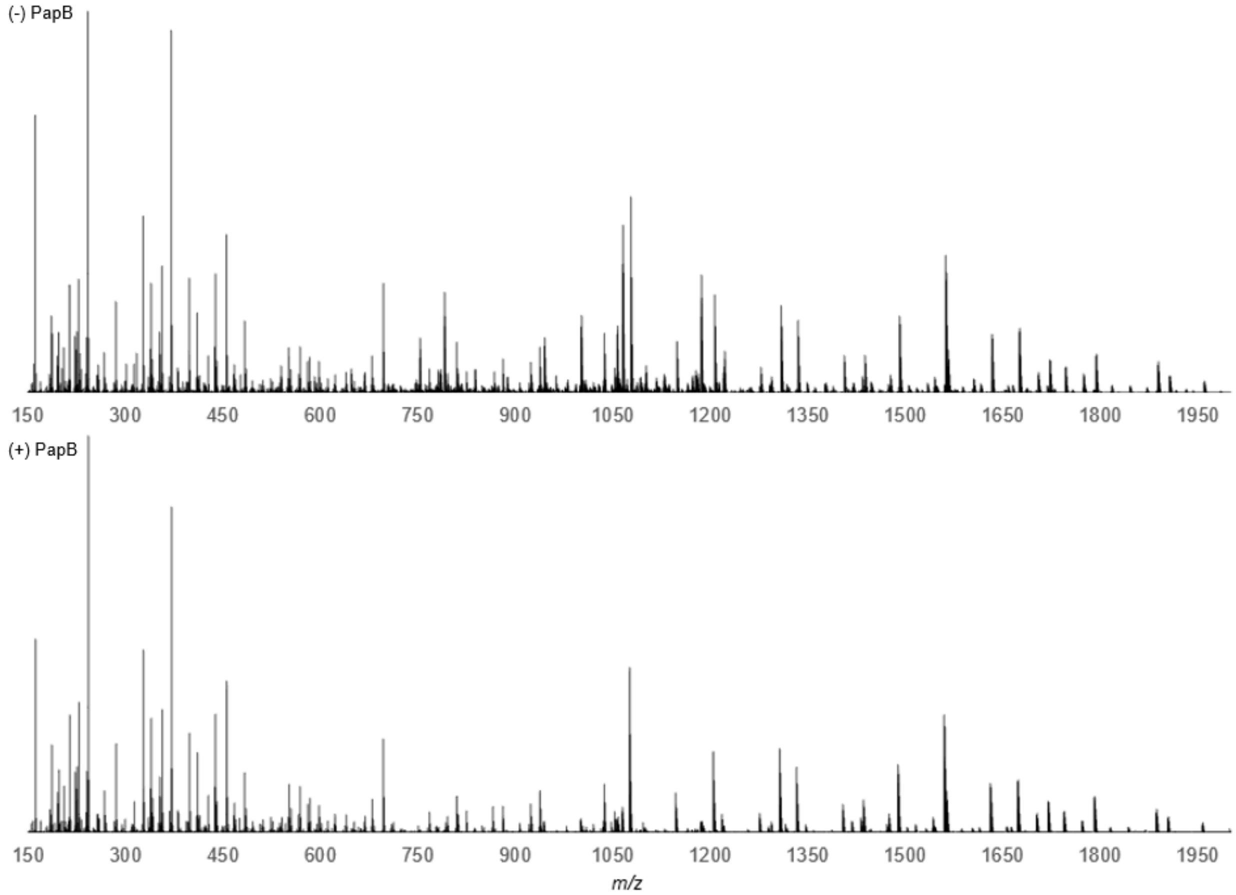

Gold highlight indicates *z* = 2 charge state.
Teal highlight indicates *z* = 3 charge state.
* Indicates the fragment originated from the modified peptide.

Ion	Sequence	Expected Monoisoptic Mass	Observed Monoisotopic Mass	Ppm Error
b2	LK	242.1863	242.1861	0.72
b2*	LK*	242.1863	242.1862	0.39
b3	LKQ	370.2449	370.2447	0.52
b3*	LKQ*	370.2449	370.2452	-0.72
b4	LKQI	483.3289	483.3296	-1.46
b4*	LKQI*	483.3289	483.3280	1.84
b5	LKQIN	597.3719	597.3721	-0.31
b5*	LKQIN*	597.3719	597.3736	-2.82
b6	LKQINV	696.4403	696.4417	-2.07
b6*	LKQINV*	696.4403	696.4395	1.21
b7	LKQINVI	809.5244	809.5247	-0.33

b7*	LKQINVI*	809.5244	809.5220	2.95
b8<br>b8*	LKQINVIA<br>LKQINVIA*	880.5615<br>880.5615	880.5601<br>880.5634	1.57<br>-2.18
b9<br>b9*	LKQINVIAG<br>LKQINVIAG*	937.5829<br>937.5829	937.5826<br>937.5795	0.32<br>3.60
b10<br>b10*	LKQINVIAGV<br>LKQINVIAGV*	1036.6513<br>1036.6513	1036.6500<br>1036.6545	1.23<br>-3.11
b11<br>b11*	LKQINVIAGVK<br>LKQINVIAGVK*	1164.7463<br>1164.7463	1164.7436<br>1164.7492	2.33<br>-2.47
b12<br>b12*	LKQINVIAGVKE<br>LKQINVIAGVKE*	1293.7889<br>1293.7889	1293.7860<br>1293.7928	2.23<br>-2.99
b13<br>b13*	LKQINVIAGVKEP<br>LKQINVIAGVKEP*	1390.8417<br>1390.8417	1390.8476<br>1390.8430	-4.27<br>-0.95
b14<br>b14*	LKQINVIAGVKEPI<br>LKQINVIAGVKEPI*	1503.9257<br>1503.9257	1503.9191<br>1503.9279	4.38<br>-1.48
b15<br>b15*	LKQINVIAGVKEPIR<br>LKQINVIAGVKEPIR*	1660.0268<br>1660.0268	1660.0259<br>1660.0203	0.57<br>3.93
b16<br>b16*	LKQINVIAGVKEPIRA<br>LKQINVIAGVKEPIRA*	1731.0640<br>1731.0640	1731.0704<br>Not Found	-3.69<br>N/A
b17<br>b17*	LKQINVIAGVKEPIRAY<br>LKQINVIAGVKEPIRAY*	947.5673<br>947.5673	947.5654<br>947.5692	2.05<br>-1.96
b18<br>b18*	LKQINVIAGVKEPIRAYG<br>LKQINVIAGVKEPIRAYG*	976.0780<br>976.0780	976.0771<br>976.0812	0.89<br>-3.24
b19<br>b19*	LKQINVIAGVKEPIRAYGC<br>(-1 Da) LKQINVIAGVKEPIRAYG**C***	1027.5826<br>1027.0787	1027.5856<br>Not Found	-2.92<br>N/A
b20<br>b20*	LKQINVIAGVKEPIRAYGCS<br>(-1 Da) LKQINVIAGVKEPIRAYG**C**S*	1071.0986<br>1070.5947	1071.1023<br>Not Found	-3.50<br>N/A
b21<br>b21*	LKQINVIAGVKEPIRAYGCSA<br>(-1 Da) LKQINVIAGVKEPIRAYG**C**SA*	1106.6172<br>1106.1133	1106.6200<br>Not Found	-2.57<br>N/A
b22<br>b22*	LKQINVIAGVKEPIRAYGCSAA<br>(-1 Da) LKQINVIAGVKEPIRAYG**C**SAA*	761.7596<br>761.4236	761.7587<br>Not Found	-3.50<br>N/A
b23<br>b23*	LKQINVIAGVKEPIRAYGCSAAN<br>(-1 Da) LKQINVIAGVKEPIRAYG**C**SAAN*	799.7739<br>799.4379	799.7731<br>Not Found	1.04<br>N/A
b24<br>b24*	LKQINVIAGVKEPIRAYGCSAAND<br>(-2 Da) LKQINVIAGVKEPIRAYG**C**SAAN**D***	838.1162<br>837.4443	838.1166<br>837.4470	-0.53<br>-3.19
[M]<br>[M]*	LKQINVIAGVKEPIRAYGCSAANDA<br>(-2 Da) LKQINVIAGVKEPIRAYG**C**SAAN**D**A*	867.7988<br>867.1269	867.7984<br>867.1261	0.46<br>0.92
y24<br>y24*	IKQINVIAGVKEPIRAYGCSAANDA<br>(-2 Da) IKQINVIAGVKEPIRAYG**C**SAAN**D**A*	830.1041<br>829.4322	Not Found<br>829.4320	N/A<br>0.27
y23<br>y23*	QINVIAGVKEPIRAYGCSAANDA<br>(-2 Da) QINVIAGVKEPIRAYG**C**SAAN**D**A*	787.4058<br>786.7339	787.4075<br>Not Found	-2.15<br>N/A
y22	INVIAGVKEPIRAYGCSAANDA	744.7196	744.7179	2.23

y22*	(-2 Da) INVIAGVKEPIRAYG**C**SAAN**D**A*	744.0477	744.0462	2.04
y21<br>y21*	NVIAGVKEPIRAYGCSAANDA<br>(-2 Da) NVIAGVKEPIRAYG**C**SAAN**D**A*	1060.0337<br>1059.0258	1060.0351<br>1059.0258	-1.32<br>0
y20<br>y20*	VIAGVKEPIRAYGCSAANDA<br>(-2 Da) VIAGVKEPIRAYG**C**SAAN**D**A*	1003.0122<br>1002.0044	1003.0122<br>1002.0079	0<br>-3.54
y19<br>y19*	IAGVKEPIRAYGCSAANDA<br>(-2 Da) IAGVKEPIRAYG**C**SAAN**D**A*	953.4780<br>952.4702	953.4781<br>952.4706	-0.14<br>-0.43
y18<br>y18*	AGVKEPIRAYGCSAANDA<br>(-2 Da) AGVKEPIRAYG**C**SAAN**D**A*	896.9360<br>895.9281	896.9361<br>895.9277	-0.12<br>0.41
y17<br>y17*	GVKEPIRAYGCSAANDA<br>(-2 Da) GVKEPIRAYG**C**SAAN**D**A*	861.4174<br>860.4096	861.4186<br>860.4096	-1.40<br>0
y16<br>y16*	VKEPIRAYGCSAANDA<br>(-2 Da) VKEPIRAYG**C**SAAN**D**A*	832.9067<br>831.8989	832.9060<br>Not Found	0.81<br>N/A
y15<br>y15*	KEPIRAYGCSAANDA<br>(-2 Da) KEPIRAYG**C**SAAN**D**A*	1565.7377<br>1563.7220	1565.7338<br>1563.7181	2.49<br>2.51
y14<br>y14*	EPIRAYGCSAANDA<br>(-2 Da) EPIRAYG**C**SAAN**D**A*	1437.6427<br>1435.6271	1437.6475<br>1435.6287	-3.36<br>-1.12
y13<br>y13*	PIRAYGCSAANDA<br>(-2 Da) PIRAYG**C**SAAN**D**A*	1308.6001<br>1306.5845	1308.5977<br>1306.5866	1.84<br>-1.64
y12<br>y12*	IRAYGCSAANDA<br>(-2 Da) IRAYG**C**SAAN**D**A*	1211.5473<br>1209.5317	1211.5449<br>1209.5337	1.98<br>-1.68
y11<br>y11*	RAYGCSAANDA<br>(-2 Da) RAYG**C**SAAN**D**A*	1098.4633<br>1096.4476	1098.4610<br>1096.4490	2.07<br>-1.25
y10<br>y10*	AYGCSAANDA<br>(-2 Da) AYG**C**SAAN**D**A*	942.3622<br>940.3465	942.3655<br>940.3492	-3.51<br>-2.82
y9<br>y9*	YGCSAANDA<br>(-2 Da) YG**C**SAAN**D**A*	871.3251<br>869.3094	871.3281<br>869.3102	-0.96<br>-0.96
y8<br>y8*	GCSAANDA<br>(-2 Da) G**C**SAAN**D**A*	708.2617<br>706.2461	708.2597<br>706.2462	2.77<br>-0.12
y7<br>y7*	CSAANDA<br>(-2 Da) **C**SAAN**D**A*	651.2403<br>649.2246	651.2398<br>649.2239	1.08<br>1.08
y6<br>y6*	SAANDA<br>(-1 Da) SAAN**D**A*	548.2311<br>547.2233	548.2317<br>Not Found	-1.04<br>N/A
y5<br>y5*	AANDA<br>(-1 Da) AAN**D**A*	461.1991<br>460.1912	461.1989<br>Not Found	0.42<br>N/A
y4<br>y4*	ANDA<br>(-1 Da) AN**D**A*	390.1619<br>389.1541	390.1614<br>Not Found	1.26<br>N/A
y3<br>y3*	NDA<br>(-1 Da) N**D**A*	319.1248<br>318.1170	319.1249<br>Not Found	-0.25<br>N/A
y2<br>y2*	DA<br>(-1 Da) **D**A*	205.0819<br>204.0741	205.0819<br>Not Found	0<br>N/A

Figure S17. Tandem mass spectrometry of unmodified and modified Leader-CX$_5$D Peptides
Sequence: LKQINVIAGVKEPIRAYG**C**SAAAN**D**A
(crosslinking C and D residues shown in **bold**)

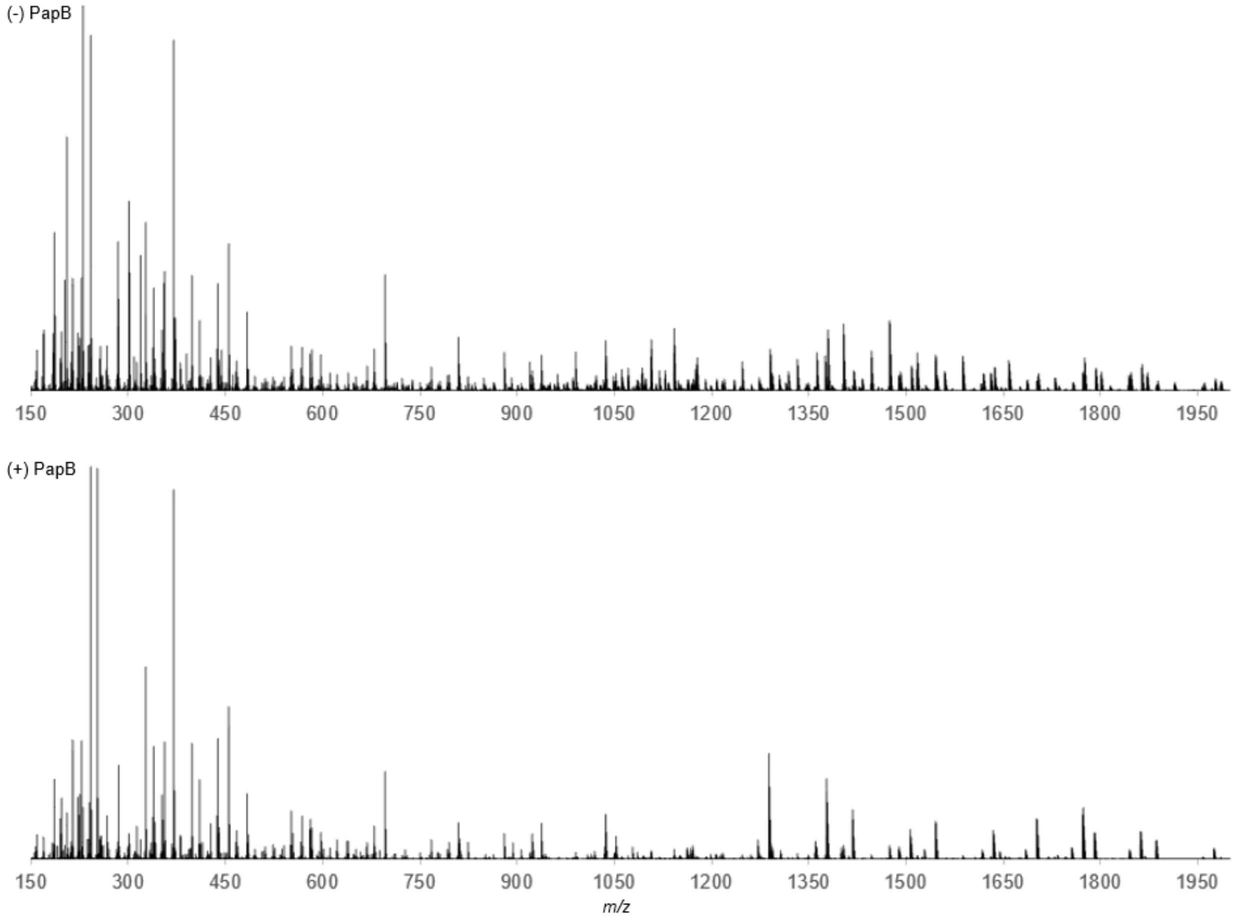

Gold highlight indicates z = 2 charge state.
Teal highlight indicates z = 3 charge state.
* Indicates the fragment originated from the modified peptide.

Ion	Sequence	Expected Monoisotopic Mass	Observed Monoisotopic Mass	Ppm error
b2	LK	242.1863	242.1861	0.67
b2*	LK*	242.1863	242.1860	1.09
b3	LKQ	370.2449	370.2448	0.33
b3*	LKQ*	370.2449	370.2446	0.71
b4	LKQI	483.3289	483.3297	-1.75
b4*	LKQI*	483.3289	483.3296	-1.39
b5	LKQIN	597.3719	597.3723	-0.70
b5*	LKQIN*	597.3719	597.3721	-0.35
b6	LKQINV	696.4403	696.4421	-2.54
b6*	LKQINV*	696.4403	696.4418	-2.20
b7	LKQINVI	809.5244	809.5251	-0.87

b7*	LKQINVI*	809.5244	809.5248	-0.53
b8	LKQINVIA	880.5615	880.5606	0.99
b8*	LKQINVIA*	880.5615	880.5603	1.33
b9	LKQINVIAG	937.5829	937.5832	-0.30
b9*	LKQINVIAG*	937.5829	937.5829	0.04
b10	LKQINVIAGV	1036.6513	1036.6507	0.55
b10*	LKQINVIAGV*	1036.6513	1036.6504	0.90
b11	LKQINVIAGVK	1164.7463	1164.7445	1.57
b11*	LKQINVIAGVK*	1164.7463	1164.7441	1.93
b12	LKQINVIAGVKE	1293.7889	1293.7871	1.41
b12*	LKQINVIAGVKE*	1293.7889	1293.7866	1.77
b13	LKQINVIAGVKEP	1390.8417	1390.8366	3.70
b13*	LKQINVIAGVKEP*	1390.8417	1390.8360	4.08
b14	LKQINVIAGVKEPI	1503.9257	1503.9205	3.45
b14*	LKQINVIAGVKEPI*	1503.9257	1503.9199	3.84
b15	LKQINVIAGVKEPIR	1660.0268	1660.0275	-0.43
b15*	LKQINVIAGVKEPIR*	1660.0268	1660.0268	-0.02
b16	LKQINVIAGVKEPIRA	1731.0640	1731.0722	-4.72
b16*	LKQINVIAGVKEPIRA*	1731.0640	1731.0714	-4.30
b17	LKQINVIAGVKEPIRAY	947.5673	947.5659	1.43
b17*	LKQINVIAGVKEPIRAY*	947.5673	947.5656	1.77
b18	LKQINVIAGVKEPIRAYG	976.0780	976.0778	0.24
b18*	LKQINVIAGVKEPIRAYG*	976.0780	976.0774	0.58
b19	LKQINVIAGVKEPIRAYGC	1027.5826	1027.5863	-3.59
b19*	(-1 Da) LKQINVIAGVKEPIRAYG**C***	1027.0787	Not Found	N/A
b20	LKQINVIAGVKEPIRAYGCS	1071.0986	1071.0948	3.56
b20*	(-1 Da) LKQINVIAGVKEPIRAYG**C**S*	1070.5947	Not Found	N/A
b21	LKQINVIAGVKEPIRAYGCSA	1106.6172	1106.6208	-3.29
b21*	(-1 Da) LKQINVIAGVKEPIRAYG**C**SA*	1106.1133	Not Found	N/A
b22	LKQINVIAGVKEPIRAYGCSAA	1142.1357	1142.1392	-3.02
b22*	(-1 Da) LKQINVIAGVKEPIRAYG**C**SAA*	1141.6318	Not Found	N/A
b23	LKQINVIAGVKEPIRAYGCSAAA	785.4386	785.4366	2.50
b23*	(-1 Da) LKQINVIAGVKEPIRAYG**C**SAAA*	785.1027	Not Found	N/A
b24	LKQINVIAGVKEPIRAYGCSAAAN	823.4529	823.4529	-0.03
b24*	(-1 Da) LKQINVIAGVKEPIRAYG**C**SAAAN*	823.1170	Not Found	N/A
b25	LKQINVIAGVKEPIRAYGCSAAAND	861.7952	861.7969	-1.94
b25*	(-2 Da) LKQINVIAGVKEPIRAYG**C**SAAAN**D***	861.1234	861.1252	-2.05
[M]	LKQINVIAGVKEPIRAYGCSAAANDA	891.4778	891.4781	-0.30
[M]*	(-2 Da) LKQINVIAGVKEPIRAYG**C**SAAAN**D**A*	890.8059	890.8029	3.38
y25	KQINVIAGVKEPIRAYGCSAAANDA	853.7831	Not Found	N/A
y25*	(-2 Da) KQINVIAGVKEPIRAYG**C**SAAAN**D**A*	853.1112	853.1112	-0.06
y24	QINVIAGVKEPIRAYGCSAAANDA	811.0848	Not Found	N/A
y24*	(-2 Da) QINVIAGVKEPIRAYG**C**SAAAN**D**A*	810.4129	810.4102	3.30
y23	INVIAGVKEPIRAYGCSAAANDA	768.3986	768.3971	1.97

y23*	(-2 Da) INVIAGVKEPIRAYG**C**SAAAN**D**A*	767.7267	767.7256	1.42
y22<br>y22*	NVIAGVKEPIRAYGCSAAANDA<br>(-2 Da) NVIAGVKEPIRAYG**C**SAAAN**D**A*	730.7039<br>730.0320	Not Found<br>730.0306	N/A<br>1.93
y21<br>y21*	VIAGVKEPIRAYGCSAAANDA<br>(-2 Da) VIAGVKEPIRAYG**C**SAAAN**D**A*	1038.5308<br>1037.5229	1038.5288<br>1037.5214	1.94<br>1.44
y20<br>y20*	IAGVKEPIRAYGCSAAANDA<br>(-2 Da) IAGVKEPIRAYG**C**SAAAN**D**A*	988.9966<br>987.9887	988.9947<br>987.9923	1.88<br>-3.63
y19<br>y19*	AGVKEPIRAYGCSAAANDA<br>(-2 Da) AGVKEPIRAYG**C**SAAAN**D**A*	932.4545<br>931.4467	932.4517<br>931.4463	3.04<br>0.45
y18<br>y18*	GVKEPIRAYGCSAAANDA<br>(-2 Da) GVKEPIRAYG**C**SAAAN**D**A*	896.9360<br>895.9281	896.9366<br>895.9309	-0.72<br>-3.13
y17<br>y17*	VKEPIRAYGCSAAANDA<br>(-2 Da) VKEPIRAYG**C**SAAAN**D**A*	868.4252<br>867.4174	868.4270<br>867.4204	-2.11<br>-3.43
y16<br>y16*	KEPIRAYGCSAAANDA<br>(-2 Da) KEPIRAYG**C**SAAAN**D**A*	818.8910<br>817.8832	818.8892<br>817.8842	2.25<br>-1.20
y15<br>y15*	EPIRAYGCSAAANDA<br>(-2 Da) EPIRAYG**C**SAAAN**D**A*	754.8435<br>753.8357	Not Found<br>753.8356	N/A<br>0.18
y14<br>y14*	PIRAYGCSAAANDA<br>(-2 Da) PIRAYG**C**SAAAN**D**A*	1379.6372<br>1377.6216	1379.6396<br>1377.6244	-1.72<br>-2.03
y13<br>y13*	IRAYGCSAAANDA<br>(-2 Da) IRAYG**C**SAAAN**D**A*	1282.5845<br>1280.5688	1282.5840<br>1280.5712	0.37<br>-1.86
y12<br>y12*	RAYGCSAAANDA<br>(-2 Da) RAYG**C**SAAAN**D**A*	1169.5004<br>1167.4847	1169.4996<br>1167.4820	0.67<br>2.28
y11<br>y11*	AYGCSAAANDA<br>(-2 Da) AYG**C**SAAAN**D**A*	1013.3993<br>1011.3836	1013.3959<br>1011.3869	3.39<br>-3.25
y10<br>y10*	YGCSAAANDA<br>(-2 Da) YG**C**SAAAN**D**A*	942.3622<br>940.3465	942.3592<br>940.3457	3.15<br>0.89
y9<br>y9*	GCSAAANDA<br>(-2 Da) G**C**SAAAN**D**A*	779.2988<br>777.2832	779.2988<br>777.2856	0.06<br>-3.14
y8<br>y8*	CSAAANDA<br>(-2 Da) **C**SAAAN**D**A*	722.2774<br>720.2617	Not Found<br>720.2620	N/A<br>-0.47
y7<br>y7*	SAAANDA<br>(-1 Da) SAAAN**D**A*	619.2682<br>618.2604	619.2665<br>Not Found	2.69<br>N/A
y6<br>y6*	AAANDA<br>(-1 Da) AAAN**D**A*	532.2362<br>531.2283	532.2351<br>Not Found	2.02<br>N/A
y5<br>y5*	AANDA<br>(-1 Da) AAN**D**A*	461.1991<br>460.1912	461.1990<br>Not Found	0.15<br>N/A
y4<br>y4*	ANDA<br>(-1 Da) AN**D**A*	390.1619<br>389.1541	390.1615<br>Not Found	1.05<br>N/A
y3<br>y3*	NDA<br>(-1 Da) N**D**A*	319.1248<br>318.1170	319.1249<br>Not Found	-0.39<br>N/A
y2<br>y2*	DA<br>(-1 Da) **D**A*	205.0819<br>204.0741	205.0819<br>Not Found	-0.20<br>N/A

Figure S18. Tandem mass spectrometry of unmodified and modified Leader-CX_6D Peptides
Sequence: LKQINVIAGVKEPIRAYG**C**SAAAAN**D**A
(crosslinking C and D residues shown in **bold**)

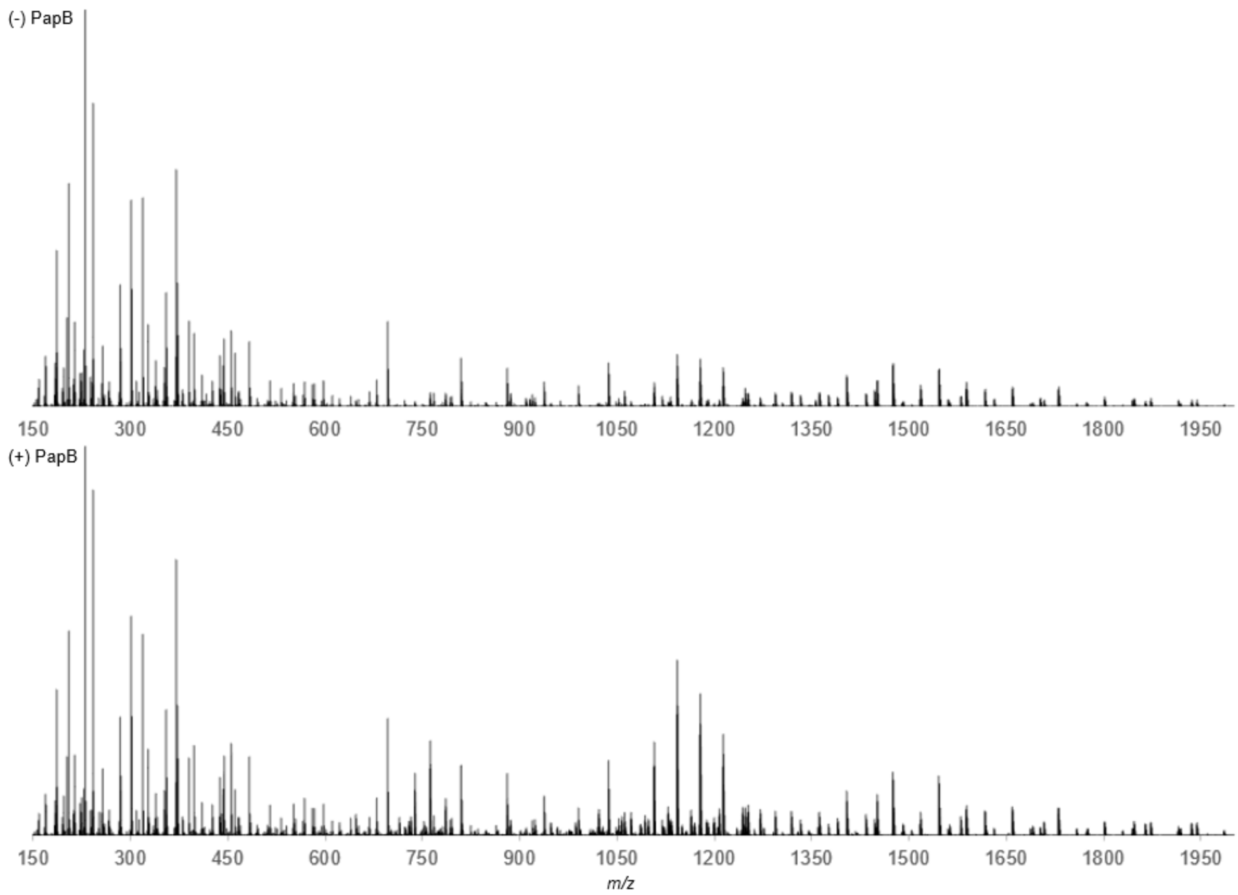

Gold highlight indicates *z* = 2 charge state.
Teal highlight indicates *z* = 3 charge state.
* Indicates the fragment originated from the modified peptide.

Ion	Sequence	Expected Monoisotopic Mass	Observed Monoisotopic Mass	Ppm Error
b2	LK	242.1863	242.1863	-0.12
b2*	LK*	242.1863	242.1861	0.64
b3	LKQ	370.2449	370.2451	-0.43
b3*	LKQ*	370.2449	370.2451	-0.48
b4	LKQI	483.3289	483.3301	-2.50
b4*	LKQI*	483.3289	483.3279	2.07
b5	LKQIN	597.3719	597.3728	-1.43
b5*	LKQIN*	597.3719	597.3735	-2.60
b6	LKQINV	696.4403	696.4382	3.00
b6*	LKQINV*	696.4403	696.4393	1.42
b7	LKQINVI	809.5244	809.5257	-1.57

b7*	LKQINVI*	809.5244	809.5218	3.15
b8<br>b8*	LKQINVIA<br>LKQINVIA*	880.5615<br>880.5615	880.5612<br>880.5632	0.29<br>-1.98
b9<br>b9*	LKQINVIAG<br>LKQINVIAG*	937.5829<br>937.5829	937.5838<br>937.5861	-0.99<br>-3.46
b10<br>b10*	LKQINVIAGV<br>LKQINVIAGV*	1036.6513<br>1036.6513	1036.6514<br>1036.6543	-0.13<br>-2.92
b11<br>b11*	LKQINVIAGVK<br>LKQINVIAGVK*	1164.7463<br>1164.7463	1164.7452<br>1164.7490	0.91<br>-2.28
b12<br>b12*	LKQINVIAGVKE<br>LKQINVIAGVKE*	1293.7889<br>1293.7889	1293.7879<br>1293.7925	0.76<br>-2.81
b13<br>b13*	LKQINVIAGVKEP<br>LKQINVIAGVKEP*	1390.8417<br>1390.8417	1390.8374<br>1390.8428	3.06<br>-0.78
b14<br>b14*	LKQINVIAGVKEPI<br>LKQINVIAGVKEPI*	1503.9257<br>1503.9257	1503.9215<br>1503.9277	2.81<br>-1.32
b15<br>b15*	LKQINVIAGVKEPIR<br>LKQINVIAGVKEPIR*	1660.0268<br>1660.0268	1660.0285<br>1660.0200	-1.05<br>4.09
b16<br>b16*	LKQINVIAGVKEPIRA<br>LKQINVIAGVKEPIRA*	1731.0640<br>1731.0640	1731.0562<br>1731.0643	4.53<br>-0.16
b17<br>b17*	LKQINVIAGVKEPIRAY<br>LKQINVIAGVKEPIRAY*	947.5673<br>947.5673	947.5666<br>947.5690	0.74<br>-1.77
b18<br>b18*	LKQINVIAGVKEPIRAYG<br>LKQINVIAGVKEPIRAYG*	976.0780<br>976.0780	976.0784<br>976.0810	-0.44<br>-3.04
b19<br>b19*	LKQINVIAGVKEPIRAYGC<br>(-1 Da) LKQINVIAGVKEPIRAYG**C***	1027.5826<br>1027.0787	1027.5792<br>Not Found	3.33<br>N/A
b20<br>b20*	LKQINVIAGVKEPIRAYGCS<br>(-1 Da) LKQINVIAGVKEPIRAYG**C**S*	1071.0986<br>1070.5947	1071.0955<br>Not Found	2.89<br>N/A
b21<br>b21*	LKQINVIAGVKEPIRAYGCSA<br>(-1 Da) LKQINVIAGVKEPIRAYG**C**SA*	1106.6172<br>1106.1133	1106.6129<br>Not Found	3.92<br>N/A
b22<br>b22*	LKQINVIAGVKEPIRAYGCSAA<br>(-1 Da) LKQINVIAGVKEPIRAYG**C**SAA*	1142.1357<br>1141.6318	1142.1399<br>Not Found	-3.69<br>N/A
b23<br>b23*	LKQINVIAGVKEPIRAYGCSAAA<br>(-1 Da) LKQINVIAGVKEPIRAYG**C**SAAA*	785.4386<br>785.1027	785.4372<br>Not Found	1.80<br>N/A
b24<br>b24*	LKQINVIAGVKEPIRAYGCSAAAA<br>(-1 Da) LKQINVIAGVKEPIRAYG**C**SAAAA*	809.1177<br>808.7817	809.1163<br>Not Found	1.78<br>N/A
b25<br>b25*	LKQINVIAGVKEPIRAYGCSAAAAN<br>(-1 Da) LKQINVIAGVKEPIRAYG**C**SAAAAN*	847.1320<br>846.7960	847.1296<br>Not Found	2.86<br>N/A
b26<br>b26*	LKQINVIAGVKEPIRAYGCSAAAAND<br>LKQINVIAGVKEPIRAYG**C**SAAAAN**D*** (-2 Da)	885.4743<br>884.8024	885.4760<br>884.8037	-1.91<br>-1.47
[M]<br>[M]*	LKQINVIAGVKEPIRAYGCSAAAANDA<br>LKQINVIAGVKEPIRAYG**C**SAAAAN**D**A* (-2 Da)	915.1568<br>914.4850	915.1578<br>914.4843	-1.12<br>0.76
y26<br>y26*	KQINVIAGVKEPIRAYGCSAAAANDA<br>KQINVIAGVKEPIRAYG**C**SAAAAN**D**A*	877.4621<br>876.7903	877.4594<br>876.7901	3.04<br>0.25

	(-2 Da)			
y25<br>y25*	QINVIAGVKEPIRAYGCSAAAANDA<br>QINVIAGVKEPIRAYG**C**SAAAAN**D**A*<br>(-2 Da)	834.7638<br>834.0919	Not Found<br>834.0898	N/A<br>2.48
y24<br>y24*	INVIAGVKEPIRAYGCSAAAANDA<br>(-2 Da) INVIAGVKEPIRAYG**C**SAAAAN**D**A*	792.0776<br>791.4058	Not Found<br>791.4065	N/A<br>-0.95
y23<br>y23*	NVIAGVKEPIRAYGCSAAAANDA<br>(-2 Da) NVIAGVKEPIRAYG**C**SAAAAN**D**A*	754.3829<br>753.7111	Not Found<br>753.7101	N/A<br>1.26
y22<br>y22*	VIAGVKEPIRAYGCSAAAANDA<br>(-2 Da) VIAGVKEPIRAYG**C**SAAAAN**D**A*	1074.0493<br>1073.0415	1074.0521<br>1073.0378	-2.60<br>3.47
y21<br>y21*	IAGVKEPIRAYGCSAAAANDA<br>(-2 Da) IAGVKEPIRAYG**C**SAAAAN**D**A*	1024.5151<br>1023.5073	1024.5168<br>1023.5096	-1.62<br>-2.22
y20<br>y20*	AGVKEPIRAYGCSAAAANDA<br>(-2 Da) AGVKEPIRAYG**C**SAAAAN**D**A*	967.9731<br>966.9653	967.9736<br>966.9630	-0.53<br>2.41
y19<br>y19*	GVKEPIRAYGCSAAAANDA<br>(-2 Da) GVKEPIRAYG**C**SAAAAN**D**A*	932.4545<br>931.4467	932.4523<br>931.4495	2.35<br>-3.03
y18<br>y18*	VKEPIRAYGCSAAAANDA<br>(-2 Da) VKEPIRAYG**C**SAAAAN**D**A*	903.9438<br>902.9360	903.9457<br>902.9370	-2.05<br>-1.07
y17<br>y17*	KEPIRAYGCSAAAANDA<br>(-2 Da) KEPIRAYG**C**SAAAAN**D**A*	854.4096<br>853.4018	854.4074<br>853.4036	2.54<br>-2.05
y16<br>y16*	EPIRAYGCSAAAANDA<br>(-2 Da) EPIRAYG**C**SAAAAN**D**A*	1579.7169<br>1577.7013	1579.7105<br>1577.6947	4.03<br>4.16
y15<br>y15*	PIRAYGCSAAAANDA<br>(-2 Da) PIRAYG**C**SAAAAN**D**A*	1450.6743<br>1448.6587	1450.6747<br>1448.6651	-0.26<br>-4.43
y14<br>y14*	IRAYGCSAAAANDA<br>(-2 Da) IRAYG**C**SAAAAN**D**A*	1353.6216<br>1351.6059	1353.6247<br>Not Found	-2.33<br>N/A
y13<br>y13*	RAYGCSAAAANDA<br>(-2 Da) RAYG**C**SAAAAN**D**A*	1240.5375<br>1238.5219	1240.5425<br>1238.5188	-4.06<br>2.52
y12<br>y12*	AYGCSAAAANDA<br>(-2 Da) AYG**C**SAAAAN**D**A*	1084.4364<br>1082.4207	1084.4336<br>Not Found	2.56<br>N/A
y11<br>y11*	YGCSAAAANDA<br>(-2 Da) YG**C**SAAAAN**D**A*	1013.3993<br>1011.3836	1013.3966<br>1011.3830	2.71<br>0.55
y10<br>y10*	GCSAAAANDA<br>(-2 Da) G**C**SAAAAN**D**A*	850.3360<br>848.3203	850.3363<br>848.3194	-0.38<br>1.11
y9<br>y9*	CSAAAANDA<br>(-2 Da) **C**SAAAAN**D**A*	793.3145<br>791.2988	793.3140<br>791.3010	0.59<br>-2.76
y8<br>y8*	SAAAANDA<br>(-1 Da) SAAAAN**D**A*	690.3053<br>689.2975	690.3043<br>Not Found	1.39<br>N/A
y7<br>y7*	AAAANDA<br>(-1 Da) AAAAN**D**A*	603.2733<br>602.2655	603.2733<br>Not Found	0.00<br>N/A
y6<br>y6*	AAANDA<br>(-1 Da) AAAN**D**A*	532.2362<br>531.2283	532.2355<br>Not Found	1.28<br>N/A
y5<br>y5*	AANDA<br>(-1 Da) AAN**D**A*	461.1991<br>460.1912	461.1994<br>Not Found	-0.60<br>N/A

y4<br>y4*	ANDA<br>(-1 Da) AN**D**A*	390.1619<br>389.1541	390.1618<br>Not Found	0.29<br>N/A
y3<br>y3*	NDA<br>(-1 Da) N**D**A*	319.1248<br>318.1170	319.1252<br>Not Found	-1.17<br>N/A
y2<br>y2*	DA<br>(-1 Da) **D**A*	205.0819<br>204.0741	205.0821<br>Not Found	-1.00<br>N/A

Table S19. Expected and Observed Monoisotopic Masses for Leader Extension Peptides
Leader = LKQINVIAGVKEPIRAYG

Sequence	Expected Monoisotopic Mass	Observed Monoisotopic Mass	Ppm Error
Leader-<br>AAA**C**SAN**D**A (z = 3)	(-) PapB: 915.1568<br>(+) PapB (-2Da): 914.4850	915.1562<br>914.4824	-0.656<br>-2.843
Leader-<br>AAA**C**SAN**D**A**C**SAN**D**A (z = 3)	(-) PapB: 1102.2186<br>(+) PapB (-4Da): 1100.8748	1102.2156<br>1100.8722	-2.722<br>-2.362
Leader-<br>AAA**C**SA**CD**AA**D**A (z = 3)	(-) PapB: 997.1793<br>(+) PapB (-4Da): 995.8355	997.1774<br>995.8310	-1.905<br>-4.519
Leader-<br>AAAASA**C**DAA**D**A (z = 3)	(-) PapB: 986.5220<br>(+) PapB (-2Da): 985.8501	986.5232<br>985.8479	1.216<br>-2.232
Leader-<br>AAA**C**SAA**D**AADA (z = 3)	(-) PapB: 986.5220<br>(+) PapB (-2Da): 985.8501	986.5261<br>985.8527	4.156<br>2.637

Figure S20. Tandem mass spectrometry analysis of unmodified and modified Leader-AAACSANDA peptides

Sequence: LKQINVIAGVKEPIRAYGAAA**C**SAN**D**A

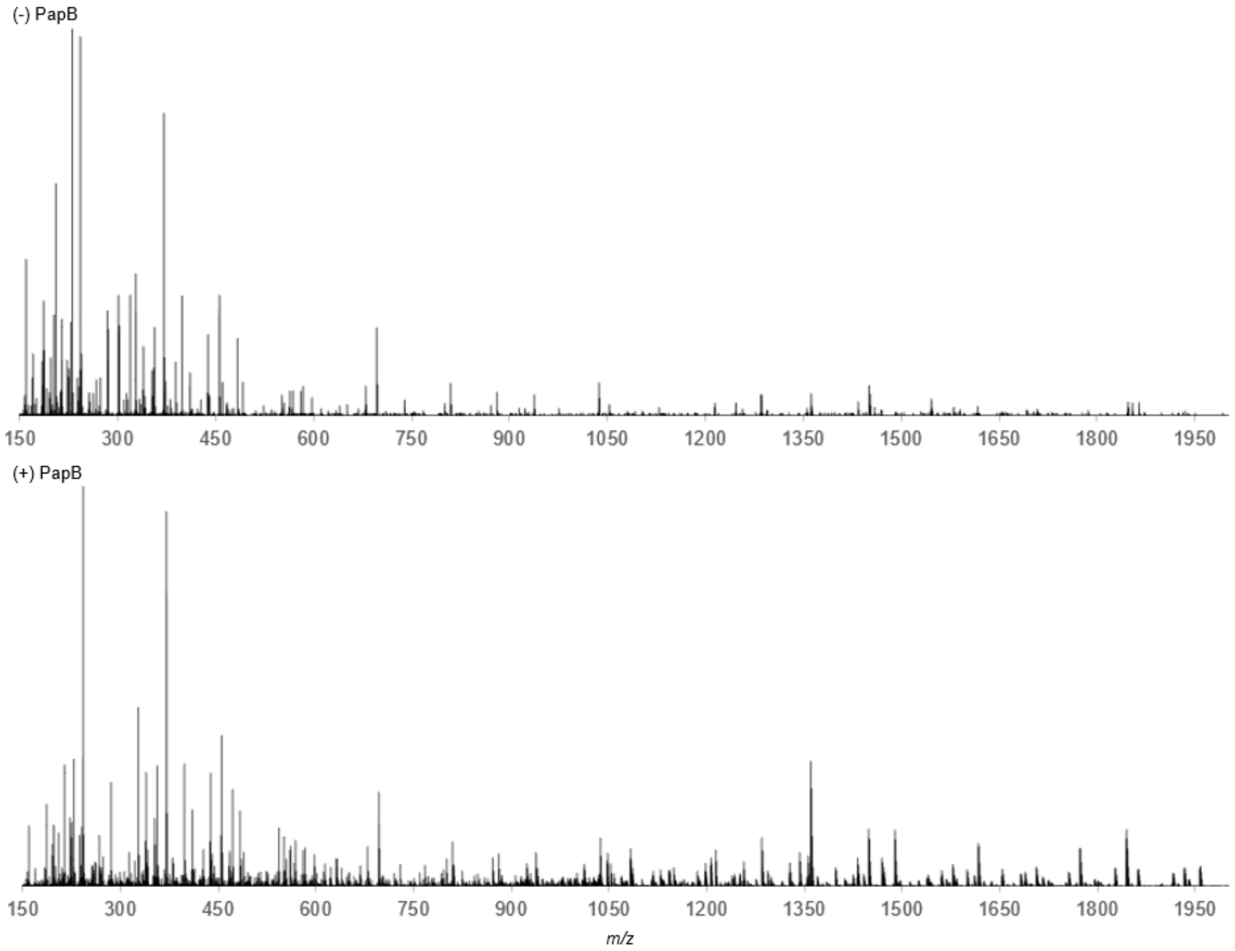

Unhighlighted indicates *z* = 1 charge state.
Gold highlight indicates *z* = 2 charge state.
Teal highlight indicates *z* = 3 charge state.
* Indicates the fragment originated from the modified peptide.

Ion	Sequence	Expected Monoisotopic Mass	Observed Monoisotopic Mass	Ppm Error
b2	LK	242.1863	242.1863	-0.01
b2*	LK*	242.1863	242.1859	1.64
b3	LKQ	370.2449	370.2448	0.38
b3*	LKQ*	370.2449	370.2441	2.20
b4	LKQI	483.3289	483.3295	-1.16
b4*	LKQI*	483.3289	483.3285	0.79
b5	LKQIN	597.3719	597.3717	0.38
b5*	LKQIN*	597.3719	597.3704	2.45

b6	LKQINV	696.4403	696.4410	-1.06
b6*	LKQINV*	696.4403	696.4395	1.09
b7	LKQINVI	809.5244	809.5236	1.02
b7*	LKQINVI*	809.5244	809.5218	3.27
b8	LKQINVIA	880.5615	880.5588	3.12
b8*	LKQINVIA*	880.5615	880.5629	-1.60
b9	LKQINVIAG	937.5829	937.5810	2.02
b9*	LKQINVIAG*	937.5829	937.5856	-2.88
b10	LKQINVIAGV	1036.6513	1036.6480	3.19
b10*	LKQINVIAGV*	1036.6513	1036.6534	-2.00
b11	LKQINVIAGVK	1164.7463	Not Found	N/A
b11*	LKQINVIAGVK*	1164.7463	1164.7474	-0.95
b12	LKQINVIAGVKE	1293.7889	1293.7937	-3.73
b12*	LKQINVIAGVKE*	1293.7889	1293.7903	-1.09
b13	LKQINVIAGVKEP	1390.8417	Not Found	N/A
b13*	LKQINVIAGVKEP*	1390.8417	1390.8400	1.22
b14	LKQINVIAGVKEPI	1503.9257	1503.9298	-2.73
b14*	LKQINVIAGVKEPI*	1503.9257	1503.9242	0.99
b15	LKQINVIAGVKEPIR	1660.0268	Not Found	N/A
b15*	LKQINVIAGVKEPIR*	1660.0268	1659.9674	35.79
b16	LKQINVIAGVKEPIRA	1731.0640	1731.0659	-1.10
b16*	LKQINVIAGVKEPIRA*	1731.0640	1731.0593	2.74
b17	LKQINVIAGVKEPIRAY	947.5673	Not Found	N/A
b17*	LKQINVIAGVKEPIRAY*	947.5673	947.5684	-1.15
b18	LKQINVIAGVKEPIRAYG	976.0780	976.0792	-1.23
b18*	LKQINVIAGVKEPIRAYG*	976.0780	976.0803	-2.33
b19	LKQINVIAGVKEPIRAYGA	1011.5966	Not Found	N/A
b19*	LKQINVIAGVKEPIRAYGA*	1011.5966	1011.5958	0.83
b20	LKQINVIAGVKEPIRAYGAA	1047.1151	1047.1161	-0.96
b20*	LKQINVIAGVKEPIRAYGAA*	1047.1151	1047.1145	0.59
b21	LKQINVIAGVKEPIRAYGAAA	1082.6337	Not Found	N/A
b21*	LKQINVIAGVKEPIRAYGAAA*	1082.6337	1082.6344	-0.69
b22	LKQINVIAGVKEPIRAYGAAAC	1134.1383	1134.1402	-1.68
b22*	(-1 Da) LKQINVIAGVKEPIRAYGAAA**C***	1133.6344	Not Found	N/A
b23	LKQINVIAGVKEPIRAYGAAACS	1177.6543	1177.6577	-2.89
b23*	(-1 Da) LKQINVIAGVKEPIRAYGAAA**C**S*	1177.1504	Not Found	N/A
b24	LKQINVIAGVKEPIRAYGAAACSA	809.1177	809.1198	-2.60
b24*	(-1 Da) LKQINVIAGVKEPIRAYGAAA**C**SA*	808.7817	Not Found	N/A
b25	LKQINVIAGVKEPIRAYGAAACSAN	847.1320	847.1344	-2.83
b25*	(-1 Da) LKQINVIAGVKEPIRAYGAAA**C**SAN*	846.7960	Not Found	N/A
b26	LKQINVIAGVKEPIRAYGAAACSAND	885.4743	Not Found	N/A
b26*	(-2 Da) LKQINVIAGVKEPIRAYGAAA**C**SAN**D***	884.8024	884.8020	-0.45
[M]	LKQINVIAGVKEPIRAYGAAACSANDA	915.1568	915.1577	-0.98
[M] *	(-2 Da) LKQINVIAGVKEPIRAYGAAA**C**SAN**D**A*	914.4850	914.4838	1.27

y26	KQINVIAGVKEPIRAYGAAACSANDA	877.4621	Not Found	N/A
y26*	(-2 Da) KQINVIAGVKEPIRAYGAAA**C**SAN**D**A*	876.7903	876.7898	0.62
y25	QINVIAGVKEPIRAYGAAACSANDA	834.7638	834.7680	-5.03
y25*	(-2 Da) QINVIAGVKEPIRAYGAAA**C**SAN**D**A*	834.0919	834.0897	2.69
y24	INVIAGVKEPIRAYGAAACSANDA	792.0776	Not Found	N/A
y24*	(-2 Da) INVIAGVKEPIRAYGAAA**C**SAN**D**A*	791.4058	Not Found	N/A
y23	NVIAGVKEPIRAYGAAACSANDA	1131.0708	1131.0697	0.97
y23*	(-2 Da) NVIAGVKEPIRAYGAAA**C**SAN**D**A*	1130.0630	1130.0669	-3.43
y22	VIAGVKEPIRAYGAAACSANDA	1074.0493	1074.0493	0.00
y22*	(-2 Da) VIAGVKEPIRAYGAAA**C**SAN**D**A*	1073.0415	1073.0450	-3.25
y21	IAGVKEPIRAYGAAACSANDA	1024.5151	Not Found	N/A
y21*	(-2 Da) IAGVKEPIRAYGAAA**C**SAN**D**A*	1023.5073	1023.5087	-1.35
y20	AGVKEPIRAYGAAACSANDA	967.9731	967.9767	-3.72
y20*	(-2 Da) AGVKEPIRAYGAAA**C**SAN**D**A*	966.9653	966.9623	3.09
y19	GVKEPIRAYGAAACSANDA	932.4545	932.4587	-4.50
y19*	(-2 Da) GVKEPIRAYGAAA**C**SAN**D**A*	931.4467	931.4490	-2.47
y18	VKEPIRAYGAAACSANDA	903.9438	Not Found	N/A
y18*	(-2 Da) VKEPIRAYGAAA**C**SAN**D**A*	902.9360	902.9366	-0.61
y17	KEPIRAYGAAACSANDA	854.4096	Not Found	N/A
y17*	(-2 Da) KEPIRAYGAAA**C**SAN**D**A*	853.4018	853.4033	-1.77
y16	EPIRAYGAAACSANDA	1579.7169	1579.7179	-0.60
y16*	(-2 Da) EPIRAYGAAA**C**SAN**D**A*	1577.7013	1577.7056	-2.75
y15	PIRAYGAAACSANDA	1450.6743	1450.6682	4.20
y15*	(-2 Da) PIRAYGAAA**C**SAN**D**A*	1448.6587	1448.6620	-2.27
y14	IRAYGAAACSANDA	1353.6216	1353.6254	-2.81
y14*	(-2 Da) IRAYGAAA**C**SAN**D**A*	1351.6059	1351.6104	-3.30
y13	RAYGAAACSANDA	1240.5375	Not Found	N/A
y13*	(-2 Da) RAYGAAA**C**SAN**D**A*	1238.5219	1238.5169	4.07
y12	AYGAAACSANDA	1084.4364	1084.4388	-2.21
y12*	(-2 Da) AYGAAA**C**SAN**D**A*	1082.4207	1082.4233	-2.43
y11	YGAAACSANDA	1013.3993	1013.4001	-0.79
y11*	(-2 Da) YGAAA**C**SAN**D**A*	1011.3836	1011.3822	1.39
y10	GAAACSANDA	850.3360	850.3392	-3.76
y10*	(-2 Da) GAAA**C**SAN**D**A*	848.3203	848.3191	1.37
y9	AAACSANDA	793.3145	Not Found	N/A
y9*	(-2 Da) AAA**C**SAN**D**A*	791.2988	791.3009	-2.71
y8	AACSANDA	722.2774	722.2773	0.08
y8*	(-2 Da) AA**C**SAN**D**A*	720.2617	720.2596	2.93
y7	ACSANDA	651.2403	651.2393	1.60
y7*	(-2 Da) A**C**SAN**D**A*	649.2246	649.2251	-0.80
y6	CSANDA	580.2032	580.2036	-0.69
y6*	(-2 Da) **C**SAN**D**A*	578.1875	578.1864	1.94
y5	SANDA	477.1940	477.1935	1.09
y5*	(-1 Da) SAN**D**A*	476.1861	Not Found	N/A

y4<br>y4*	ANDA<br>(-1 Da) AN**D**A*	390.1619<br>389.1541	390.1614<br>Not Found	1.20<br>N/A
y3<br>y3*	NDA<br>(-1 Da) N**D**A*	319.1248<br>318.1170	319.1250<br>Not Found	-0.62<br>N/A
y2<br>y2*	DA<br>(-1 Da) **D**A*	205.0819<br>204.0741	205.0821<br>Not Found	-1.13<br>N/A

Figure S21. Tandem mass spectrometry analysis of unmodified and modified Leader-AAACSANDACSANDA peptides
Sequence: LKQINVIAGVKEPIRAYGAAA**C**SAN**D**A**C**SAN**D**A

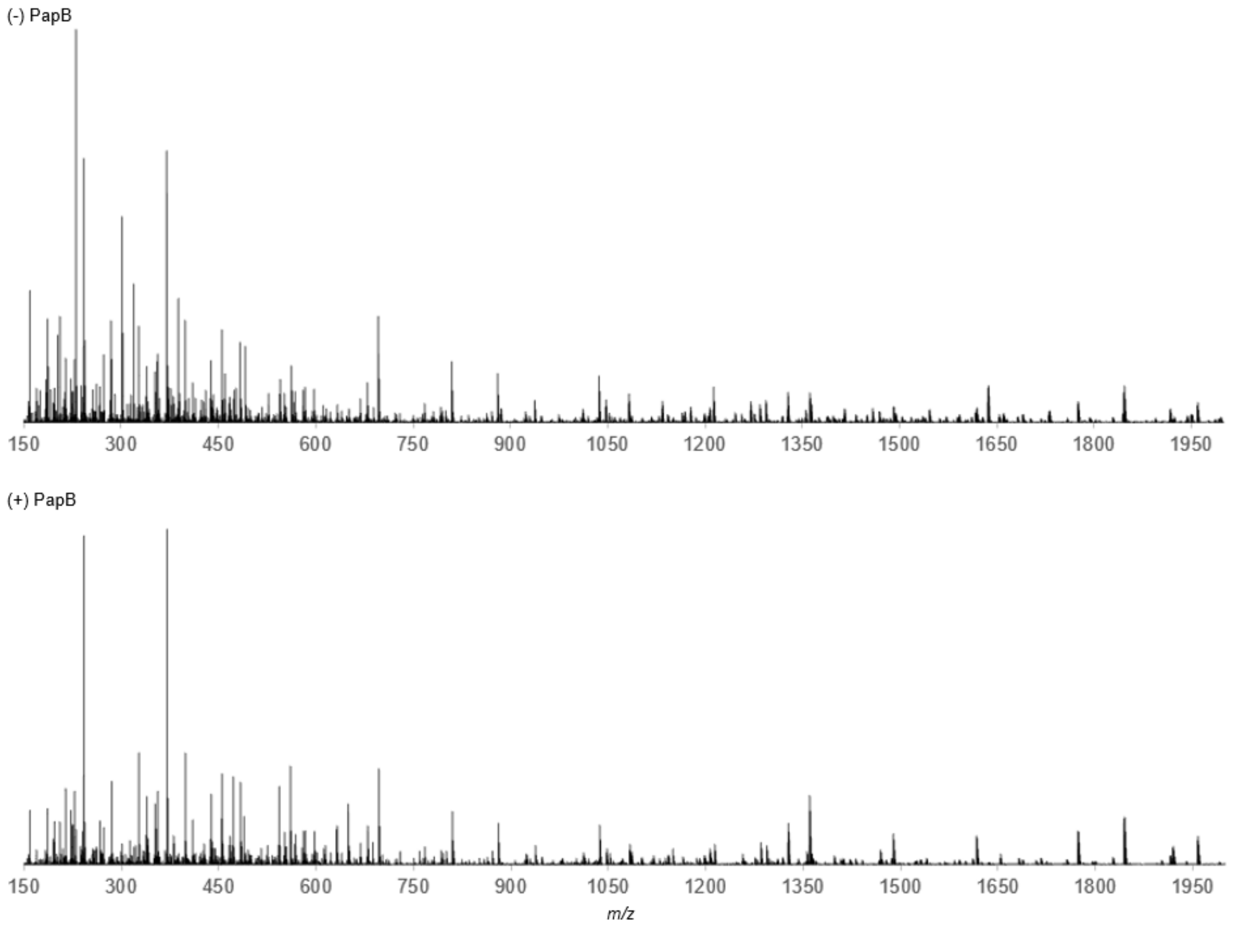

Unhighlighted indicates *z* = 1 charge state.
Gold highlight indicates *z* = 2 charge state.
Teal highlight indicates *z* = 3 charge state.
* Indicates the fragment originated from the modified peptide.

Ion	Sequence	Expected Monoisotopic Mass	Observed Monoisotopic Mass	Ppm Error
b2	LK	242.1863	242.1860	1.42
b2*	LK*	242.1863	242.1860	1.45
b3	LKQ	370.2449	370.2443	1.62
b3*	LKQ*	370.2449	370.2441	2.14
b4	LKQI	483.3289	483.3289	-0.06
b4*	LKQI*	483.3289	483.3285	0.82
b5	LKQIN	597.3719	597.3711	1.35
b5*	LKQIN*	597.3719	597.3704	2.57
b6	LKQINV	696.4403	696.4404	-0.20

b6*	LKQINV*	696.4403	696.4394	1.29
b7	LKQINVI	809.5244	809.5230	1.77
b7*	LKQINVI*	809.5244	809.5270	-3.21
b8	LKQINVIA	880.5615	880.5643	-3.22
b8*	LKQINVIA*	880.5615	880.5626	-1.29
b9	LKQINVIAG	937.5829	937.5804	2.66
b9*	LKQINVIAG*	937.5829	937.5853	-2.53
b10	LKQINVIAGV	1036.6513	1036.6474	3.75
b10*	LKQINVIAGV*	1036.6513	1036.6530	-1.61
b11	LKQINVIAGVK	1164.7463	1164.7498	-3.03
b11*	LKQINVIAGVK*	1164.7463	1164.7469	-0.49
b12	LKQINVIAGVKE	1293.7889	1293.7933	-3.36
b12*	LKQINVIAGVKE*	1293.7889	1293.7896	-0.56
b13	LKQINVIAGVKEP	1390.8417	1390.8433	-1.19
b13*	LKQINVIAGVKEP*	1390.8417	1390.8146	19.48
b14	LKQINVIAGVKEPI	1503.9257	1503.9281	-1.57
b14*	LKQINVIAGVKEPI*	1503.9257	1503.9233	1.62
b15	LKQINVIAGVKEPIR	1660.0268	1660.0201	4.04
b15*	LKQINVIAGVKEPIR*	1660.0268	1660.0304	-2.15
b16	LKQINVIAGVKEPIRA	1731.0640	1731.0642	-0.12
b16*	LKQINVIAGVKEPIRA*	1731.0640	1731.0580	3.46
b17	LKQINVIAGVKEPIRAY	947.5673	947.5700	-2.88
b17*	LKQINVIAGVKEPIRAY*	947.5673	947.5681	-0.80
b18	LKQINVIAGVKEPIRAYG	976.0780	976.0748	3.30
b18*	LKQINVIAGVKEPIRAYG*	976.0780	976.0799	-1.96
b19	LKQINVIAGVKEPIRAYGA	1011.5966	1011.5976	-1.01
b19*	LKQINVIAGVKEPIRAYGA*	1011.5966	1011.5954	1.22
b20	LKQINVIAGVKEPIRAYGAA	1047.1151	1047.1165	-1.31
b20*	LKQINVIAGVKEPIRAYGAA*	1047.1151	1047.1141	0.99
b21	LKQINVIAGVKEPIRAYGAAA	1082.6337	1082.6366	-2.64
b21*	LKQINVIAGVKEPIRAYGAAA*	1082.6337	1082.6340	-0.27
b22	LKQINVIAGVKEPIRAYGAAAC	1134.1383	1134.1423	-3.53
b22*	(-1 Da) LKQINVIAGVKEPIRAYGAAA**C***	1133.6344	Not Found	N/A
b23	LKQINVIAGVKEPIRAYGAAACS	1177.6543	1177.6549	-0.55
b23*	(-1 Da) LKQINVIAGVKEPIRAYGAAA**C**S*	1177.1504	Not Found	N/A
b24	LKQINVIAGVKEPIRAYGAAACSA	1213.1728	1213.1729	-0.07
b24*	(-1 Da) LKQINVIAGVKEPIRAYGAAA**C**SA*	1212.6689	Not Found	N/A
b25	LKQINVIAGVKEPIRAYGAAACSAN	847.1320	847.1325	-0.60
b25*	(-1 Da) LKQINVIAGVKEPIRAYGAAA**C**SAN*	846.7960	Not Found	N/A
b26	LKQINVIAGVKEPIRAYGAAACSAND	885.4743	885.4729	1.62
b26*	(-2 Da) LKQINVIAGVKEPIRAYGAAA**C**SAN**D***	884.8024	884.8031	-0.77
b27	LKQINVIAGVKEPIRAYGAAACSANDA	909.1533	909.1520	1.45
b27*	(-2 Da) LKQINVIAGVKEPIRAYGAAA**C**SAN**D**A*	908.4814	908.4811	0.34
b28	LKQINVIAGVKEPIRAYGAAACSANDAC	943.4897	943.4891	0.59

b28*	(-3 Da) LKQINVIAGVKEPIRAYGAAA**C**SAN**D**A**C***	942.4819	Not Found	N/A
b29<br>b29*	LKQINVIAGVKEPIRAYGAAACSANDACS<br>LKQINVIAGVKEPIRAYGAAA**C**SAN**D**A**C**S*<br>(-3 Da)	972.5004<br>971.4926	972.4985<br>Not Found	1.96<br>N/A
b30<br>b30*	LKQINVIAGVKEPIRAYGAAACSANDACSA<br>LKQINVIAGVKEPIRAYGAAA**C**SAN**D**A**C**SA*<br>(-3 Da)	996.1794<br>995.1716	Not Found<br>Not Found	N/A<br>N/A
b31<br>b31*	LKQINVIAGVKEPIRAYGAAACSANDACSAN<br>LKQINVIAGVKEPIRAYGAAA**C**SAN**D**A**C**SAN*<br>(-3 Da)	1034.1937<br>1033.1859	Not Found<br>Not Found	N/A<br>N/A
b32<br>b32*	LKQINVIAGVKEPIRAYGAAACSANDACSAND<br>LKQINVIAGVKEPIRAYGAAA**C**SAN**D**A**C**SAN**D***<br>(-4 Da)	1072.5360<br>1071.1923	1072.5389<br>1071.1885	-2.69<br>3.55
[M]<br>[M]*	LKQINVIAGVKEPIRAYGAAACSANDACSANDA<br>LKQINVIAGVKEPIRAYGAAA**C**SAN**D**A**C**SAN**D**A*<br>(-4 Da)	1102.2186<br>1100.8748	1102.2185<br>1100.8764	-0.09<br>-1.42
y32<br>y32*	KQINVIAGVKEPIRAYGAAACSANDACSANDA<br>KQINVIAGVKEPIRAYGAAA**C**SAN**D**A**C**SAN**D**A*<br>(-4 Da)	1064.5239<br>1063.1802	1064.5226<br>1063.1791	1.24<br>1.04
y31<br>y31*	QINVIAGVKEPIRAYGAAACSANDACSANDA<br>QINVIAGVKEPIRAYGAAA**C**SAN**D**A**C**SAN**D**A*<br>(-4 Da)	1021.8256<br>1020.4818	Not Found<br>1020.4795	N/A<br>2.27
y30<br>y30*	INVIAGVKEPIRAYGAAACSANDACSANDA<br>INVIAGVKEPIRAYGAAA**C**SAN**D**A**C**SAN**D**A*<br>(-4 Da)	979.1394<br>977.7956	Not Found<br>Not Found	N/A<br>N/A
y29<br>y29*	NVIAGVKEPIRAYGAAACSANDACSANDA<br>NVIAGVKEPIRAYGAAA**C**SAN**D**A**C**SAN**D**A*<br>(-4 Da)	941.4447<br>940.1010	941.4449<br>940.1020	-0.19<br>-1.07
y28<br>y28*	VIAGVKEPIRAYGAAACSANDACSANDA<br>VIAGVKEPIRAYGAAA**C**SAN**D**A**C**SAN**D**A*<br>(-4 Da)	903.4304<br>902.0866	903.4336<br>902.0877	-3.53<br>-1.24
y27<br>y27*	IAGVKEPIRAYGAAACSANDACSANDA<br>(-4 Da) IAGVKEPIRAYGAAA**C**SAN**D**A**C**SAN**D**A*	870.4076<br>869.0638	870.4060<br>Not Found	1.79<br>N/A
y26<br>y26*	AGVKEPIRAYGAAACSANDACSANDA<br>(-4 Da) AGVKEPIRAYGAAA**C**SAN**D**A**C**SAN**D**A*	832.7129<br>831.3691	Not Found<br>Not Found	N/A<br>N/A
y25<br>y25*	GVKEPIRAYGAAACSANDACSANDA<br>(-4 Da) GVKEPIRAYGAAA**C**SAN**D**A**C**SAN**D**A*	809.0339<br>807.6901	809.0317<br>Not Found	2.71<br>N/A
y24<br>y24*	VKEPIRAYGAAACSANDACSANDA<br>(-4 Da) VKEPIRAYGAAA**C**SAN**D**A**C**SAN**D**A*	1184.5364<br>1182.5208	Not Found<br>Not Found	N/A<br>N/A
y23<br>y23*	KEPIRAYGAAACSANDACSANDA<br>(-4 Da) KEPIRAYGAAA**C**SAN**D**A**C**SAN**D**A*	1135.0022<br>1132.9866	1135.0031<br>1132.9903	-0.78<br>-3.25
y22<br>y22*	EPIRAYGAAACSANDACSANDA<br>(-4 Da) EPIRAYGAAA**C**SAN**D**A**C**SAN**D**A*	1070.9548<br>1068.9391	Not Found<br>1068.9394	N/A<br>-0.30
y21<br>y21*	PIRAYGAAACSANDACSANDA<br>(-4 Da) PIRAYGAAA**C**SAN**D**A**C**SAN**D**A*	1006.4335<br>1004.4178	1006.4302<br>1004.4174	3.29<br>0.44
y20<br>y20*	IRAYGAAACSANDACSANDA<br>(-4 Da) IRAYGAAA**C**SAN**D**A**C**SAN**D**A*	957.9071<br>955.8914	Not Found<br>Not Found	N/A<br>N/A

y19<br>y19*	RAYGAAACSANDACSANDA<br>(-4 Da) RAYGAAA**C**SAN**D**A**C**SAN**D**A*	901.3650<br>899.3494	Not Found<br>899.3510	N/A<br>-1.76
y18<br>y18*	AYGAAACSANDACSANDA<br>(-4 Da) AYGAAA**C**SAN**D**A**C**SAN**D**A*	823.3145<br>821.2988	823.3163<br>821.2910	-2.16<br>9.55
y17<br>y17*	YGAAACSANDACSANDA<br>(-4 Da) YGAAA**C**SAN**D**A**C**SAN**D**A*	787.7959<br>785.7803	Not Found<br>785.7778	N/A<br>3.14
y16<br>y16*	GAAACSANDACSANDA<br>(-4 Da) GAAA**C**SAN**D**A**C**SAN**D**A*	1411.5213<br>1407.4900	1411.5230<br>1407.4913	-1.18<br>-0.91
y15<br>y15*	AAACSANDACSANDA<br>(-4 Da) AAA**C**SAN**D**A**C**SAN**D**A*	1354.4998<br>1350.4685	Not Found<br>1350.4675	N/A<br>0.72
y14<br>y14*	AACSANDACSANDA<br>(-4 Da) AA**C**SAN**D**A**C**SAN**D**A*	1283.4627<br>1279.4314	1283.4618<br>1279.4341	0.68<br>-2.12
y13<br>y13*	ACSANDACSANDA<br>(-4 Da) A**C**SAN**D**A**C**SAN**D**A*	1212.4256<br>1208.3943	1212.4218<br>1208.3950	3.10<br>-0.55
y12<br>y12*	CSANDACSANDA<br>(-4 Da) **C**SAN**D**A**C**SAN**D**A*	1141.3885<br>1137.3572	1141.3852<br>1137.3611	2.92<br>-3.43
y11<br>y11*	SANDACSANDA<br>(-3 Da) SAN**D**A**C**SAN**D**A*	1038.3793<br>1035.3558	1038.3826<br>Not Found	-3.22<br>N/A
y10<br>y10*	ANDACSANDA<br>(-3 Da) AN**D**A**C**SAN**D**A*	951.3473<br>948.3238	951.3504<br>Not Found	-3.24<br>N/A
y9<br>y9*	NDACSANDA<br>(-3 Da) N**D**A**C**SAN**D**A*	880.3101<br>877.2867	880.3104<br>Not Found	-0.32<br>N/A
y8<br>y8*	DACSANDA<br>(-3 Da) **D**A**C**SAN**D**A*	766.2672<br>763.2437	766.2684<br>Not Found	-1.61<br>N/A
y7<br>y7*	ACSANDA<br>(-2 Da) A**C**SAN**D**A*	651.2403<br>649.2246	651.2387<br>649.2250	2.52<br>-0.64
y6<br>y6*	CSANDA<br>(-2 Da) **C**SAN**D**A*	580.2032<br>578.1875	580.2030<br>Not Found	0.39<br>N/A
y5<br>y5*	SANDA<br>(-1 Da) SAN**D**A*	477.1940<br>476.1861	477.1930<br>Not Found	2.20<br>N/A
y4<br>y4*	ANDA<br>(-1 Da) AN**D**A*	390.1619<br>389.1541	390.1628<br>Not Found	-2.27<br>N/A
y3<br>y3*	NDA<br>(-1 Da) N**D**A*	319.1248<br>318.1170	319.1246<br>Not Found	0.70<br>N/A
y2<br>y2*	DA<br>(-1 Da) **D**A*	205.0819<br>204.0741	205.0818<br>Not Found	0.37<br>N/A

Figure S22. Tandem mass spectrometry analysis of unmodified and modified Leader-AAACSACDAADApeptides
Sequence: LKQINVIAGVKEPIRAYGAAA**C**SA**CD**AA**D**A

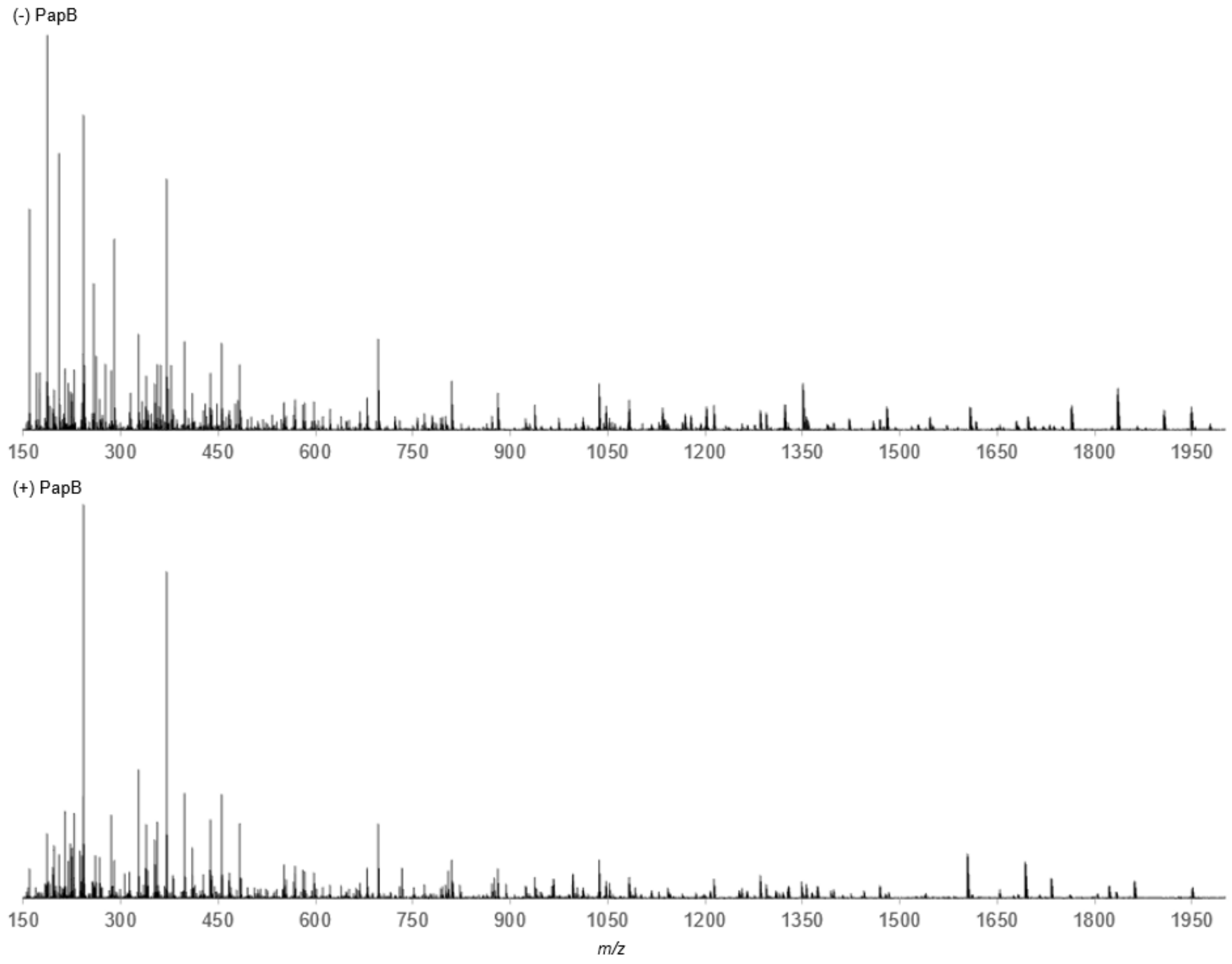

Unhighlighted indicates $z = 1$ charge state.
Gold highlight indicates $z = 2$ charge state.
Teal highlight indicates $z = 3$ charge state.
* Indicates the fragment originated from the modified peptide.

Ion	Sequence	Expected Monoisotopic Mass	Observed Monoisotopic Mass	Ppm Error
b2	LK	242.1863	242.1859	1.58
b2*	LK*	242.1863	242.1860	1.19
b3	LKQ	370.2449	370.2452	-0.82
b3*	LKQ*	370.2449	370.2455	-1.52
b4	LKQI	483.3289	483.3297	-1.69
b4*	LKQI*	483.3289	483.3277	2.59
b5	LKQIN	597.3719	597.3716	0.44
b5*	LKQIN*	597.3719	597.3723	-0.70

b6<br>b6*	LKQINV<br>LKQINV*	696.4403<br>696.4403	696.4407<br>696.4416	-0.54<br>-1.85
b7<br>b7*	LKQINVI<br>LKQINVI*	809.5244<br>809.5244	809.5228<br>809.5240	2.03<br>0.55
b8<br>b8*	LKQINVIA<br>LKQINVIA*	880.5615<br>880.5615	880.5638<br>880.5590	-2.61<br>2.84
b9<br>b9*	LKQINVIAG<br>LKQINVIAG*	937.5829<br>937.5829	937.5796<br>937.5811	3.55<br>1.89
b10<br>b10*	LKQINVIAGV<br>LKQINVIAGV*	1036.6513<br>1036.6513	1036.6539<br>1036.6479	-2.54<br>3.29
b11<br>b11*	LKQINVIAGVK<br>LKQINVIAGVK*	1164.7463<br>1164.7463	1164.7476<br>1164.7499	-1.14<br>-3.10
b12<br>b12*	LKQINVIAGVKE<br>LKQINVIAGVKE*	1293.7889<br>1293.7889	1293.7901<br>1293.7929	-0.94<br>-3.06
b13<br>b13*	LKQINVIAGVKEP<br>LKQINVIAGVKEP*	1390.8417<br>1390.8417	1390.8395<br>Not Found	1.62<br>N/A
b14<br>b14*	LKQINVIAGVKEPI<br>LKQINVIAGVKEPI*	1503.9257<br>1503.9257	1503.9232<br>1503.9268	1.66<br>-0.70
b15<br>b15*	LKQINVIAGVKEPIR<br>LKQINVIAGVKEPIR*	1660.0268<br>1660.0268	1660.0298<br>Not Found	-1.84<br>N/A
b16<br>b16*	LKQINVIAGVKEPIRA<br>LKQINVIAGVKEPIRA*	1731.0640<br>1731.0640	1731.0572<br>Not Found	3.90<br>N/A
b17<br>b17*	LKQINVIAGVKEPIRAY<br>LKQINVIAGVKEPIRAY*	947.5673<br>947.5673	947.5691<br>947.5707	-1.95<br>-3.63
b18<br>b18*	LKQINVIAGVKEPIRAYG<br>LKQINVIAGVKEPIRAYG*	976.0780<br>976.0780	976.0810<br>976.0754	-3.04<br>2.65
b19<br>b19*	LKQINVIAGVKEPIRAYGA<br>LKQINVIAGVKEPIRAYGA*	1011.5966<br>1011.5966	1011.5964<br>1011.5982	0.22<br>-1.55
b20<br>b20*	LKQINVIAGVKEPIRAYGAA<br>LKQINVIAGVKEPIRAYGAA*	1047.1151<br>1047.1151	1047.1150<br>1047.1169	0.08<br>-1.73
b21<br>b21*	LKQINVIAGVKEPIRAYGAAA<br>LKQINVIAGVKEPIRAYGAAA*	1082.6337<br>1082.6337	1082.6349<br>1082.6369	-1.10<br>-2.96
b22<br>b22*	LKQINVIAGVKEPIRAYGAAAC<br>(-1 Da) LKQINVIAGVKEPIRAYGAAA**C***	1134.1383<br>1133.6344	1134.1403<br>Not Found	-1.76<br>N/A
b23<br>b23*	LKQINVIAGVKEPIRAYGAAACS<br>(-1 Da) LKQINVIAGVKEPIRAYGAAA**C**S*	1177.6543<br>1177.1504	1177.6526<br>Not Found	1.41<br>N/A
b24<br>b24*	LKQINVIAGVKEPIRAYGAAACSA<br>(-1 Da) LKQINVIAGVKEPIRAYGAAA**C**SA*	1213.1728<br>1212.6689	1213.1703<br>Not Found	2.03<br>N/A
b25<br>b25*	LKQINVIAGVKEPIRAYGAAACSAC<br>(-2 Da) LKQINVIAGVKEPIRAYGAAA**C**SA**C***	843.4540<br>842.7822	843.4554<br>Not Found	-1.68<br>N/A
b26<br>b26*	LKQINVIAGVKEPIRAYGAAACSACD<br>LKQINVIAGVKEPIRAYGAAA**C**SA**C**D* (-3 Da)	881.7964<br>880.7885	881.7980<br>Not Found	-1.76<br>N/A
b27	LKQINVIAGVKEPIRAYGAAACSACDA	905.4754	905.4773	-2.10

b27*	LKQINVIAGVKEPIRAYGAAA**C**SA**CD**A* (-3 Da)	904.4676	Not Found	N/A
b28<br>b28*	LKQINVIAGVKEPIRAYGAAACSACDAA<br>LKQINVIAGVKEPIRAYGAAA**C**SA**CD**AA* (-3 Da)	929.1544<br>928.1466	929.1557<br>Not Found	-1.40<br>N/A
b29<br>b29*	LKQINVIAGVKEPIRAYGAAACSACDAAD<br>LKQINVIAGVKEPIRAYGAAA**C**SA**CD**AA**D*** (-4 Da)	967.4968<br>966.1530	967.4979<br>966.1525	-1.17<br>0.55
[M]<br>[M]*	LKQINVIAGVKEPIRAYGAAACSACDAADA<br>LKQINVIAGVKEPIRAYGAAA**C**SA**CD**AA**D**A* (-4 Da)	997.1793<br>995.8355	997.1821<br>995.8339	-2.82<br>1.61
y29<br>y29*	KQINVIAGVKEPIRAYGAAACSACDAADA<br>KQINVIAGVKEPIRAYGAAA**C**SA**CD**AA**D**A* (-4 Da)	959.4846<br>958.1409	959.4855<br>958.1426	-0.92<br>-1.80
y28<br>y28*	QINVIAGVKEPIRAYGAAACSACDAADA<br>QINVIAGVKEPIRAYGAAA**C**SA**CD**AA**D**A* (-4 Da)	916.7863<br>915.4425	916.7838<br>915.4442	2.69<br>-1.85
y27<br>y27*	INVIAGVKEPIRAYGAAACSACDAADA<br>INVIAGVKEPIRAYGAAA**C**SA**CD**AA**D**A* (-4 Da)	874.1001<br>872.7563	Not Found<br>872.7557	N/A<br>0.70
y26<br>y26*	NVIAGVKEPIRAYGAAACSACDAADA<br>NVIAGVKEPIRAYGAAA**C**SA**CD**AA**D**A* (-4 Da)	836.4054<br>835.0617	836.4028<br>835.0637	3.10<br>-2.36
y25<br>y25*	VIAGVKEPIRAYGAAACSACDAADA<br>VIAGVKEPIRAYGAAA**C**SA**CD**AA**D**A* (-4 Da)	798.3911<br>797.0473	798.3895<br>797.0499	2.01<br>-3.20
y24<br>y24*	IAGVKEPIRAYGAAACSACDAADA<br>(-4 Da) IAGVKEPIRAYGAAA**C**SA**CD**AA**D**A*	765.3683<br>764.0245	765.3690<br>Not Found	-0.90<br>N/A
y23<br>y23*	AGVKEPIRAYGAAACSACDAADA<br>(-4 Da) AGVKEPIRAYGAAA**C**SA**CD**AA**D**A*	1091.0068<br>1088.9911	1091.0108<br>1088.9907	-3.65<br>0.37
y22<br>y22*	GVKEPIRAYGAAACSACDAADA<br>(-4 Da) GVKEPIRAYGAAA**C**SA**CD**AA**D**A*	1055.4882<br>1053.4726	Not Found<br>1053.4763	N/A<br>-3.47
y21<br>y21*	VKEPIRAYGAAACSACDAADA<br>(-4 Da) VKEPIRAYGAAA**C**SA**CD**AA**D**A*	1026.9775<br>1024.9619	Not Found<br>1024.9644	N/A<br>-2.46
y20<br>y20*	KEPIRAYGAAACSACDAADA<br>(-4 Da) KEPIRAYGAAA**C**SA**CD**AA**D**A*	977.4433<br>975.4276	977.4417<br>975.4250	1.67<br>2.63
y19<br>y19*	EPIRAYGAAACSACDAADA<br>(-4 Da) EPIRAYGAAA**C**SA**CD**AA**D**A*	913.3958<br>911.3802	Not Found<br>911.3797	N/A<br>0.59
y18<br>y18*	PIRAYGAAACSACDAADA<br>(-4 Da) PIRAYGAAA**C**SA**CD**AA**D**A*	848.8745<br>846.8589	848.8769<br>846.8587	-2.80<br>0.19
y17<br>y17*	IRAYGAAACSACDAADA<br>(-4 Da) IRAYGAAA**C**SA**CD**AA**D**A*	800.3481<br>798.3325	Not Found<br>Not Found	N/A<br>N/A
y16<br>y16*	RAYGAAACSACDAADA<br>(-4 Da) RAYGAAA**C**SA**CD**AA**D**A*	1486.6049<br>1482.5736	1486.6094<br>1482.5713	-3.01<br>1.55
y15<br>y15*	AYGAAACSACDAADA<br>(-4 Da) AYGAAA**C**SA**CD**AA**D**A*	1330.5038<br>1326.4725	Not Found<br>1326.4740	N/A<br>-1.13

y14<br>y14*	YGAAACSACDAADA<br>(-4 Da) YGAAA**C**SA**CD**AA**D**A*	1259.4667<br>1255.4354	Not Found<br>1255.4320	N/A<br>2.72
y13<br>y13*	GAAACSACDAADA<br>(-4 Da) GAAA**C**SA**CD**AA**D**A*	1096.4034<br>1092.3721	1096.4049<br>1092.3726	-1.38<br>-0.46
y12<br>y12*	AAACSACDAADA<br>(-4 Da) AAA**C**SA**CD**AA**D**A*	1039.3819<br>1035.3506	Not Found<br>1035.3513	N/A<br>-0.67
y11<br>y11*	AACSACDAADA<br>(-4 Da) AA**C**SA**CD**AA**D**A*	968.3448<br>964.3135	Not Found<br>964.3110	N/A<br>2.60
y10<br>y10*	ACSACDAADA<br>(-4 Da) A**C**SA**CD**AA**D**A*	897.3077<br>893.2764	897.3093<br>893.2777	-1.82<br>-1.41
y9<br>y9*	CSACDAADA<br>(-4 Da) **C**SA**CD**AA**D**A*	826.2706<br>822.2393	826.2701<br>822.2373	0.63<br>2.43
y8<br>y8*	SACDAADA<br>(-3 Da) SA**CD**AA**D**A*	723.2614<br>720.2379	723.2628<br>Not Found	-1.91<br>N/A
y7<br>y7*	ACDAADA<br>(-3 Da) A**CD**AA**D**A*	636.2294<br>633.2059	636.2309<br>Not Found	-2.38<br>N/A
y6<br>y6*	CDAADA<br>(-3 Da) **CD**AA**D**A*	565.1923<br>562.1688	565.1928<br>Not Found	-0.87<br>N/A
y5<br>y5*	DAADA<br>(-2 Da) **D**AA**D**A*	462.1831<br>460.1674	462.1823<br>Not Found	1.82<br>N/A
y4<br>y4*	AADA<br>(-1 Da) AA**D**A*	347.1561<br>346.1483	347.1562<br>Not Found	-0.21<br>N/A
y3<br>y3*	ADA<br>(-1 Da) A**D**A*	276.1190<br>275.1112	276.1186<br>Not Found	1.50<br>N/A
y2<br>y2*	DA<br>(-1 Da) **D**A*	205.0819<br>204.0741	205.0819<br>Not Found	-0.14<br>N/A

Figure S23. Tandem mass spectrometry analysis of unmodified and modified Leader-AAAASACDAADA
Sequence: LKQINVIAGVKEPIRAYGAAAASA**C**DAA**D**A

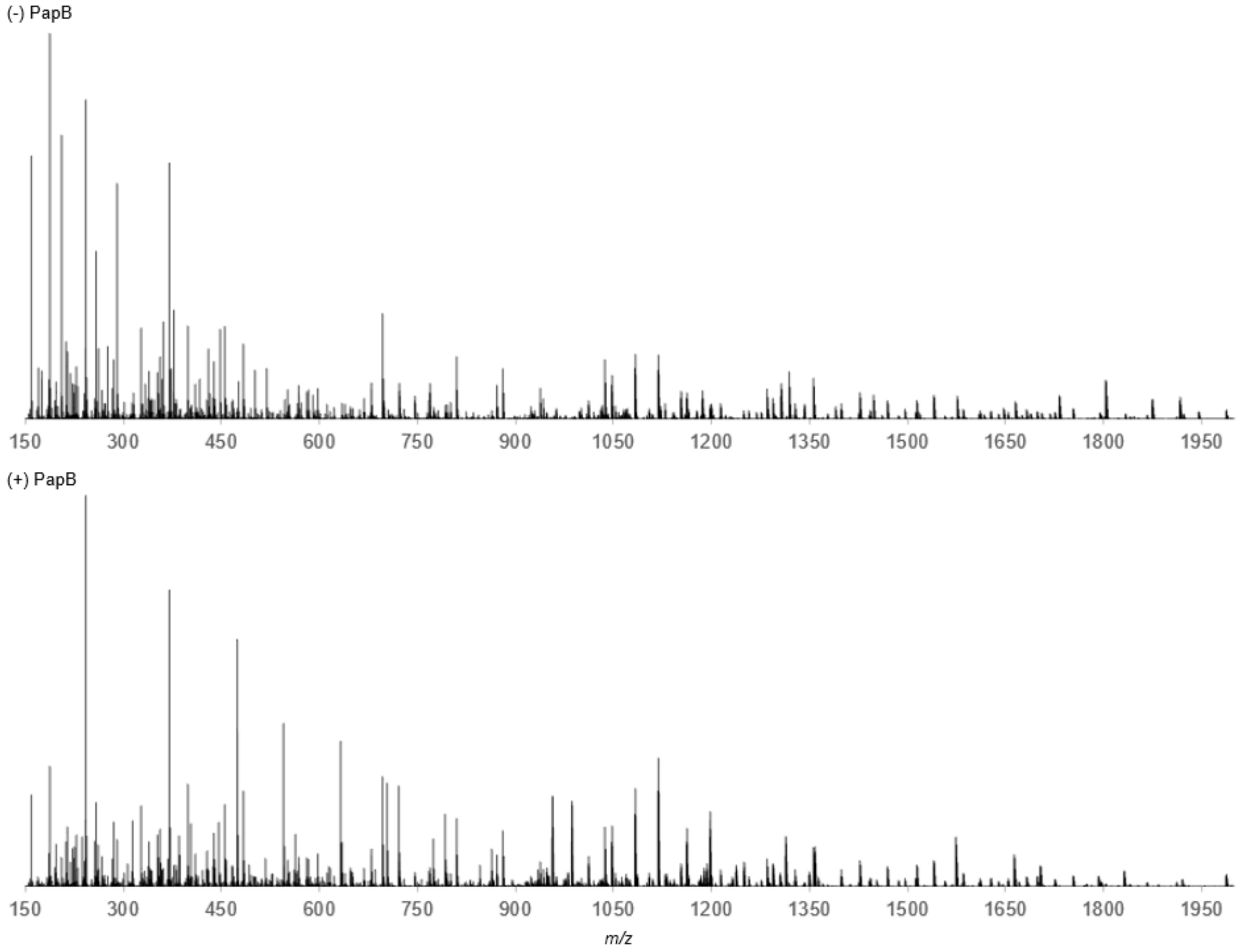

Unhighlighted indicates z = 1 charge state.
Gold highlight indicates z = 2 charge state.
Teal highlight indicates z = 3 charge state.
* Indicates the fragment originated from the modified peptide.

Ion	Sequence	Expected Monoisotopic Mass	Observed Monoisotopic Mass	Ppm Error
b2	LK	242.1863	242.1861	0.69
b2*	LK*	242.1863	242.1862	0.54
b3	LKQ	370.2449	370.2444	1.30
b3*	LKQ*	370.2449	370.2446	0.91
b4	LKQI	483.3289	483.3289	-0.09
b4*	LKQI*	483.3289	483.3292	-0.65
b5	LKQIN	597.3719	597.3709	1.59
b5*	LKQIN*	597.3719	597.3714	0.88

b6	LKQINV	696.4403	696.4401	0.26
b6*	LKQINV*	696.4403	696.4407	-0.57
b7	LKQINVI	809.5244	809.5224	2.45
b7*	LKQINVI*	809.5244	809.5232	1.50
b8	LKQINVIA	880.5615	880.5636	-2.41
b8*	LKQINVIA*	880.5615	880.5645	-3.44
b9	LKQINVIAG	937.5829	937.5795	3.58
b9*	LKQINVIAG*	937.5829	937.5806	2.50
b10	LKQINVIAGV	1036.6513	1036.6542	-2.79
b10*	LKQINVIAGV*	1036.6513	1036.6475	3.66
b11	LKQINVIAGVK	1164.7463	1164.7483	-1.73
b11*	LKQINVIAGVK*	1164.7463	1164.7498	-3.03
b12	LKQINVIAGVKE	1293.7889	1293.7913	-1.86
b12*	LKQINVIAGVKE*	1293.7889	1293.7931	-3.26
b13	LKQINVIAGVKEP	1390.8417	Not Found	N/A
b13*	LKQINVIAGVKEP*	1390.8417	Not Found	N/A
b14	LKQINVIAGVKEPI	1503.9257	1503.9253	0.24
b14*	LKQINVIAGVKEPI*	1503.9257	1503.9277	-1.33
b15	LKQINVIAGVKEPIR	1660.0268	1660.0328	-3.59
b15*	LKQINVIAGVKEPIR*	1660.0268	1660.0195	4.39
b16	LKQINVIAGVKEPIRA	866.0356	866.0382	-3.00
b16*	LKQINVIAGVKEPIRA*	866.0356	866.0330	2.96
b17	LKQINVIAGVKEPIRAY	947.5673	947.5691	-1.95
b17*	LKQINVIAGVKEPIRAY*	947.5673	947.5702	-3.04
b18	LKQINVIAGVKEPIRAYG	976.0780	976.0810	-3.12
b18*	LKQINVIAGVKEPIRAYG*	976.0780	976.0749	3.16
b19	LKQINVIAGVKEPIRAYGA	1011.5966	1011.5966	0.04
b19*	LKQINVIAGVKEPIRAYGA*	1011.5966	1011.5977	-1.12
b20	LKQINVIAGVKEPIRAYGAA	1047.1151	1047.1153	-0.20
b20*	LKQINVIAGVKEPIRAYGAA*	1047.1151	1047.1166	-1.39
b21	LKQINVIAGVKEPIRAYGAAA	1082.6337	1082.6353	-1.47
b21*	LKQINVIAGVKEPIRAYGAAA*	1082.6337	1082.6366	-2.70
b22	LKQINVIAGVKEPIRAYGAAAA	1118.1522	1118.1565	-3.82
b22*	LKQINVIAGVKEPIRAYGAAAA*	1118.1522	1118.1490	2.85
b23	LKQINVIAGVKEPIRAYGAAAAS	774.7813	774.7822	-1.12
b23*	LKQINVIAGVKEPIRAYGAAAAS*	774.7813	774.7829	-2.04
b24	LKQINVIAGVKEPIRAYGAAAASA	798.4603	798.4587	2.04
b24*	LKQINVIAGVKEPIRAYGAAAASA*	798.4603	798.4594	1.10
b25	LKQINVIAGVKEPIRAYGAAAASAC	832.7967	832.7954	1.61
b25*	(-1 Da) LKQINVIAGVKEPIRAYGAAAASA**C***	832.4608	Not Found	N/A
b26	LKQINVIAGVKEPIRAYGAAAASACD	871.1390	871.1364	2.97
b26*	LKQINVIAGVKEPIRAYGAAAASA**C**D* (-1 Da)	870.8031	Not Found	N/A
b27	LKQINVIAGVKEPIRAYGAAAASACDA	894.8180	894.8162	1.96

b27*	LKQINVIAGVKEPIRAYGAAAASA**C**DA* (-1 Da)	894.4821	Not Found	N/A
b28<br>b28*	LKQINVIAGVKEPIRAYGAAAASACDAA<br>LKQINVIAGVKEPIRAYGAAAASA**C**DAA* (-1 Da)	918.4971<br>918.1611	918.4973<br>Not Found	-0.24<br>N/A
b29<br>b29*	LKQINVIAGVKEPIRAYGAAAASACDAAD<br>LKQINVIAGVKEPIRAYGAAAASA**C**DAA**D*** (-2 Da)	956.8394<br>956.1675	Not Found<br>956.1692	N/A<br>-1.78
[M]<br>[M]*	LKQINVIAGVKEPIRAYGAAAASACDAADA<br>LKQINVIAGVKEPIRAYGAAAASA**C**DAA**D**A* (-2 Da)	986.5220<br>985.8501	986.5250<br>985.8506	-3.03<br>-0.48
y29<br>y29*	KQINVIAGVKEPIRAYGAAAASACDAADA<br>KQINVIAGVKEPIRAYGAAAASA**C**DAA**D**A* (-2 Da)	948.8273<br>948.1554	Not Found<br>948.1583	N/A<br>-3.05
y28<br>y28*	QINVIAGVKEPIRAYGAAAASACDAADA<br>QINVIAGVKEPIRAYGAAAASA**C**DAA**D**A* (-2 Da)	906.1289<br>905.4571	Not Found<br>Not Found	N/A<br>N/A
y27<br>y27*	INVIAGVKEPIRAYGAAAASACDAADA<br>INVIAGVKEPIRAYGAAAASA**C**DAA**D**A* (-2 Da)	863.4428<br>862.7709	863.4457<br>862.7732	-3.36<br>-2.70
y26<br>y26*	NVIAGVKEPIRAYGAAAASACDAADA<br>NVIAGVKEPIRAYGAAAASA**C**DAA**D**A* (-2 Da)	825.7481<br>825.0762	Not Found<br>Not Found	N/A<br>N/A
y25<br>y25*	VIAGVKEPIRAYGAAAASACDAADA<br>VIAGVKEPIRAYGAAAASA**C**DAA**D**A* (-2 Da)	787.7338<br>787.0619	Not Found<br>Not Found	N/A<br>N/A
y24<br>y24*	IAGVKEPIRAYGAAAASACDAADA<br>(-2 Da) IAGVKEPIRAYGAAAASA**C**DAA**D**A*	754.7110<br>754.0391	754.7127<br>Not Found	-2.21<br>N/A
y23<br>y23*	AGVKEPIRAYGAAAASACDAADA<br>(-2 Da) AGVKEPIRAYGAAAASA**C**DAA**D**A*	1075.0208<br>1074.0129	1075.0237<br>1074.0145	-2.70<br>-1.52
y22<br>y22*	GVKEPIRAYGAAAASACDAADA<br>(-2 Da) GVKEPIRAYGAAAASA**C**DAA**D**A*	1039.5022<br>1038.4944	Not Found<br>1038.4938	N/A<br>0.57
y21<br>y21*	VKEPIRAYGAAAASACDAADA<br>(-2 Da) VKEPIRAYGAAAASA**C**DAA**D**A*	1010.9915<br>1009.9836	1010.9942<br>1009.9824	-2.66<br>1.14
y20<br>y20*	KEPIRAYGAAAASACDAADA<br>(-2 Da) KEPIRAYGAAAASA**C**DAA**D**A*	961.4573<br>960.4494	961.4545<br>960.4527	2.88<br>-3.42
y19<br>y19*	EPIRAYGAAAASACDAADA<br>(-2 Da) EPIRAYGAAAASA**C**DAA**D**A*	897.4098<br>896.4020	Not Found<br>896.3995	N/A<br>2.78
y18<br>y18*	PIRAYGAAAASACDAADA<br>(-2 Da) PIRAYGAAAASA**C**DAA**D**A*	832.8885<br>831.8807	832.8865<br>831.8795	2.36<br>1.39
y17<br>y17*	IRAYGAAAASACDAADA<br>(-2 Da) IRAYGAAAASA**C**DAA**D**A*	784.3621<br>783.3543	Not Found<br>Not Found	N/A<br>N/A
y16<br>y16*	RAYGAAAASACDAADA<br>(-2 Da) RAYGAAAASA**C**DAA**D**A*	727.8201<br>726.8122	Not Found<br>726.8131	N/A<br>-1.26
y15<br>y15*	AYGAAAASACDAADA<br>(-2 Da) AYGAAAASA**C**DAA**D**A*	1298.5318<br>1296.5161	Not Found<br>1296.5117	N/A<br>3.41

y14<br>y14*	YGAAAASACDAADA<br>(-2 Da) YGAAAASA**C**DAA**D**A*	1227.4946<br>1225.4790	Not Found<br>1225.4783	N/A<br>0.56
y13<br>y13*	GAAAASACDAADA<br>(-2 Da) GAAAASA**C**DAA**D**A*	1064.4313<br>1062.4157	1064.4308<br>1062.4177	0.48<br>-1.85
y12<br>y12*	AAAASACDAADA<br>(-2 Da) AAAASA**C**DAA**D**A*	1007.4099<br>1005.3942	Not Found<br>1005.3940	N/A<br>0.19
y11<br>y11*	AAASACDAADA<br>(-2 Da) AAASA**C**DAA**D**A*	936.3727<br>934.3571	936.3759<br>934.3557	-3.44<br>1.46
y10<br>y10*	AASACDAADA<br>(-2 Da) AASA**C**DAA**D**A*	865.3356<br>863.3200	865.3377<br>863.3203	-2.40<br>-0.30
y9<br>y9*	ASACDAADA<br>(-2 Da) ASA**C**DAA**D**A*	794.2985<br>792.2829	794.2972<br>792.2849	1.66<br>-2.50
y8<br>y8*	SACDAADA<br>(-2 Da) SA**C**DAA**D**A*	723.2614<br>721.2457	723.2623<br>721.2472	-1.20<br>-2.04
y7<br>y7*	ACDAADA<br>(-2 Da) A**C**DAA**D**A*	636.2294<br>634.2137	636.2303<br>634.2152	-1.37<br>-2.30
y6<br>y6*	CDAADA<br>(-2 Da) **C**DAA**D**A*	565.1923<br>563.1766	565.1921<br>563.1781	0.41<br>-2.61
y5<br>y5*	DAADA<br>(-1 Da) DAA**D**A*	462.1831<br>461.1752	462.1838<br>Not Found	-1.59<br>N/A
y4<br>y4*	AADA<br>(-1 Da) AA**D**A*	347.1561<br>346.1483	347.1554<br>Not Found	2.02<br>N/A
y3<br>y3*	ADA<br>(-1 Da) A**D**A*	276.1190<br>275.1112	276.1190<br>Not Found	0.16<br>N/A
y2<br>y2*	DA<br>(-1 Da) **D**A*	205.0819<br>204.0741	205.0820<br>Not Found	-0.49<br>N/A

Figure S24. Tandem mass spectrometry analysis of unmodified and modified Leader-AAACSAADAAADA
Sequence: LKQINVIAGVKEPIRAYGAAA**C**SAA**D**AAADA

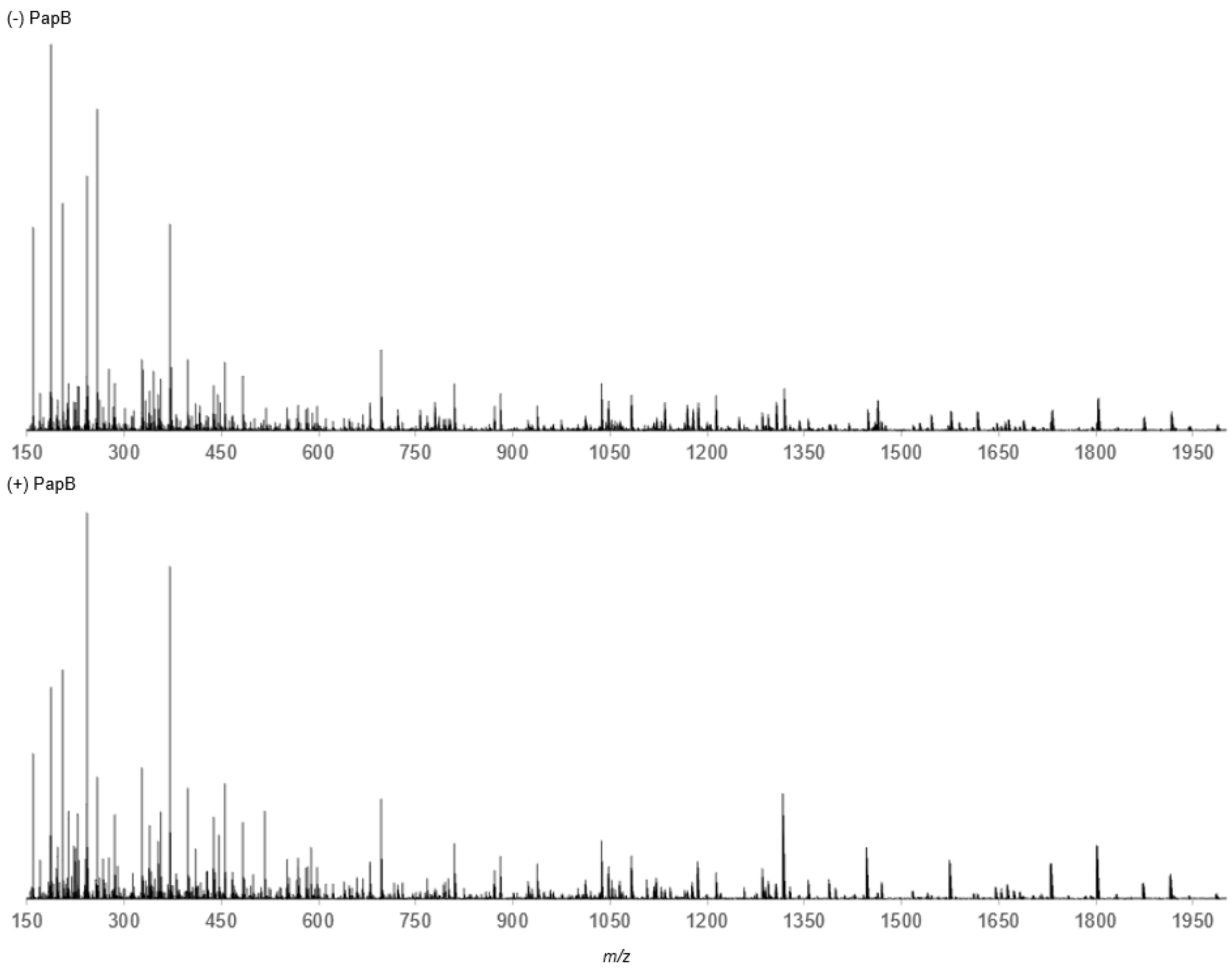

Unhighlighted indicates *z* = 1 charge state.
Gold highlight indicates *z* = 2 charge state.
Teal highlight indicates *z* = 3 charge state.
* Indicates the fragment originated from the modified peptide.

Ion	Sequence	Expected Monoisotopic Mass	Observed Monoisotopic Mass	Ppm Error
b2	LK	242.1863	242.1860	1.18
b2*	LK*	242.1863	242.1861	0.71
b3	LKQ	370.2449	370.2442	1.89
b3*	LKQ*	370.2449	370.2444	1.35
b4	LKQI	483.3289	483.3286	0.59
b4*	LKQI*	483.3289	483.3289	0.00
b5	LKQIN	597.3719	597.3705	2.35
b5*	LKQIN*	597.3719	597.3709	1.71

b6	LKQINV	696.4403	696.4396	1.07
b6*	LKQINV*	696.4403	696.4400	0.40
b7	LKQINVI	809.5244	809.5217	3.33
b7*	LKQINVI*	809.5244	809.5223	2.63
b8	LKQINVIA	880.5615	880.5628	-1.49
b8*	LKQINVIA*	880.5615	880.5635	-2.21
b9	LKQINVIAG	937.5829	937.5855	-2.73
b9*	LKQINVIAG*	937.5829	937.5862	-3.47
b10	LKQINVIAGV	1036.6513	1036.6532	-1.80
b10*	LKQINVIAGV*	1036.6513	1036.6540	-2.56
b11	LKQINVIAGVK	1164.7463	1164.7471	-0.68
b11*	LKQINVIAGVK*	1164.7463	1164.7480	-1.47
b12	LKQINVIAGVKE	1293.7889	1293.7899	-0.75
b12*	LKQINVIAGVKE*	1293.7889	1293.7909	-1.57
b13	LKQINVIAGVKEP	1390.8417	1390.8395	1.61
b13*	LKQINVIAGVKEP*	1390.8417	1390.8406	0.78
b14	LKQINVIAGVKEPI	1503.9257	1503.9235	1.44
b14*	LKQINVIAGVKEPI*	1503.9257	1503.9248	0.58
b15	LKQINVIAGVKEPIR	1660.0268	1660.0307	-2.33
b15*	LKQINVIAGVKEPIR*	1660.0268	1660.0321	-3.22
b16	LKQINVIAGVKEPIRA	866.0356	866.0374	-2.09
b16*	LKQINVIAGVKEPIRA*	866.0356	866.0380	-2.81
b17	LKQINVIAGVKEPIRAY	947.5673	947.5682	-1.00
b17*	LKQINVIAGVKEPIRAY*	947.5673	947.5689	-1.74
b18	LKQINVIAGVKEPIRAYG	976.0780	976.0801	-2.16
b18*	LKQINVIAGVKEPIRAYG*	976.0780	976.0808	-2.91
b19	LKQINVIAGVKEPIRAYGA	1011.5966	1011.5956	1.02
b19*	LKQINVIAGVKEPIRAYGA*	1011.5966	1011.5963	0.26
b20	LKQINVIAGVKEPIRAYGAA	1047.1151	1047.1143	0.80
b20*	LKQINVIAGVKEPIRAYGAA*	1047.1151	1047.1151	0.03
b21	LKQINVIAGVKEPIRAYGAAA	1082.6337	1082.6342	-0.46
b21*	LKQINVIAGVKEPIRAYGAAA*	1082.6337	1082.6350	-1.23
b22	LKQINVIAGVKEPIRAYGAAAC	1134.1383	1134.1397	-1.23
b22*	(-1 Da) LKQINVIAGVKEPIRAYGAAA**C***	1133.6344	Not Found	N/A
b23	LKQINVIAGVKEPIRAYGAAACS	1177.6543	1177.6521	1.84
b23*	(-1 Da) LKQINVIAGVKEPIRAYGAAA**C**S*	1177.1504	Not Found	N/A
b24	LKQINVIAGVKEPIRAYGAAACSA	809.1177	809.1177	-0.06
b24*	(-1 Da) LKQINVIAGVKEPIRAYGAAA**C**SA*	808.7817	Not Found	N/A
b25	LKQINVIAGVKEPIRAYGAAACSAA	832.7967	832.7946	2.50
b25*	(-1 Da) LKQINVIAGVKEPIRAYGAAA**C**SAA*	832.4608	Not Found	N/A
b26	LKQINVIAGVKEPIRAYGAAACSAAD	871.1390	871.1417	-3.12
b26*	(-2 Da) LKQINVIAGVKEPIRAYGAAA**C**SAA**D***	870.4671	870.4661	1.19
b27	LKQINVIAGVKEPIRAYGAAACSAADA	894.8180	894.8154	2.88
b27*	(-2 Da) LKQINVIAGVKEPIRAYGAAA**C**SAA**D**A*	894.1462	894.1437	2.75

b28<br>b28*	LKQINVIAGVKEPIRAYGAAACSAADAA<br>LKQINVIAGVKEPIRAYGAAA**C**SAA**D**AA* (-2 Da)	918.4971<br>917.8252	918.4965<br>Not Found	0.69<br>N/A
b29<br>b29*	LKQINVIAGVKEPIRAYGAAACSAADAAD<br>LKQINVIAGVKEPIRAYGAAA**C**SAA**D**AAD* (-2 Da)	956.8394<br>956.1675	Not Found<br>956.1679	N/A<br>-0.47
[M]<br>[M]*	LKQINVIAGVKEPIRAYGAAACSAADAADA<br>LKQINVIAGVKEPIRAYGAAA**C**SAA**D**AADA* (-2 Da)	986.5220<br>985.8501	986.5240<br>Not Found	-2.06<br>N/A
y29<br>y29*	KQINVIAGVKEPIRAYGAAACSAADAADA<br>KQINVIAGVKEPIRAYGAAA**C**SAA**D**AADA* (-2 Da)	948.8273<br>948.1554	Not Found<br>Not Found	N/A<br>N/A
y28<br>y28*	QINVIAGVKEPIRAYGAAACSAADAADA<br>QINVIAGVKEPIRAYGAAA**C**SAA**D**AADA* (-2 Da)	906.1289<br>905.4571	Not Found<br>905.4576	N/A<br>-0.58
y27<br>y27*	INVIAGVKEPIRAYGAAACSAADAADA<br>(-2 Da) INVIAGVKEPIRAYGAAA**C**SAA**D**AADA*	863.4428<br>862.7709	Not Found<br>862.7722	N/A<br>-1.50
y26<br>y26*	NVIAGVKEPIRAYGAAACSAADAADA<br>(-2 Da) NVIAGVKEPIRAYGAAA**C**SAA**D**AADA*	825.7481<br>825.0762	Not Found<br>825.0771	N/A<br>-1.09
y25<br>y25*	VIAGVKEPIRAYGAAACSAADAADA<br>(-2 Da) VIAGVKEPIRAYGAAA**C**SAA**D**AADA*	787.7338<br>787.0619	787.7348<br>787.0596	-1.30<br>2.93
y24<br>y24*	IAGVKEPIRAYGAAACSAADAADA<br>(-2 Da) IAGVKEPIRAYGAAA**C**SAA**D**AADA*	754.7110<br>754.0391	Not Found<br>754.0395	N/A<br>-0.57
y23<br>y23*	AGVKEPIRAYGAAACSAADAADA<br>(-2 Da) AGVKEPIRAYGAAA**C**SAA**D**AADA*	1075.0208<br>1074.0129	1075.0226<br>1074.0130	-1.69<br>-0.06
y22<br>y22*	GVKEPIRAYGAAACSAADAADA<br>(-2 Da) GVKEPIRAYGAAA**C**SAA**D**AADA*	1039.5022<br>1038.4944	1039.5000<br>Not Found	2.13<br>N/A
y21<br>y21*	VKEPIRAYGAAACSAADAADA<br>(-2 Da) VKEPIRAYGAAA**C**SAA**D**AADA*	1010.9915<br>1009.9836	Not Found<br>Not Found	N/A<br>N/A
y20<br>y20*	KEPIRAYGAAACSAADAADA<br>(-2 Da) KEPIRAYGAAA**C**SAA**D**AADA*	961.4573<br>960.4494	961.4607<br>960.4514	-3.52<br>-2.10
y19<br>y19*	EPIRAYGAAACSAADAADA<br>(-2 Da) EPIRAYGAAA**C**SAA**D**AADA*	897.4098<br>896.4020	Not Found<br>Not Found	N/A<br>N/A
y18<br>y18*	PIRAYGAAACSAADAADA<br>(-2 Da) PIRAYGAAA**C**SAA**D**AADA*	832.8885<br>831.8807	Not Found<br>831.8786	N/A<br>2.55
y17<br>y17*	IRAYGAAACSAADAADA<br>(-2 Da) IRAYGAAA**C**SAA**D**AADA*	1567.7169<br>1565.7013	Not Found<br>1565.6957	N/A<br>3.59
y16<br>y16*	RAYGAAACSAADAADA<br>(-2 Da) RAYGAAA**C**SAA**D**AADA*	1454.6329<br>1452.6172	1454.6279<br>1452.6187	3.41<br>-1.05
y15<br>y15*	AYGAAACSAADAADA<br>(-2 Da) AYGAAA**C**SAA**D**AADA*	1298.5318<br>1296.5161	1298.5363<br>Not Found	-3.47<br>N/A
y14<br>y14*	YGAAACSAADAADA<br>(-2 Da) YGAAA**C**SAA**D**AADA*	1227.4946<br>1225.4790	Not Found<br>1225.4763	N/A<br>2.19
y13<br>y13*	GAAACSAADAADA<br>(-2 Da) GAAA**C**SAA**D**AADA*	1064.4313<br>1062.4157	Not Found<br>1062.4161	N/A<br>-0.41

y12<br>y12*	AAACSAADAADA<br>(-2 Da) AAA**C**SAA**D**AADA*	1007.4099<br>1005.3942	Not Found<br>Not Found	N/A<br>N/A
y11<br>y11*	AACSAADAADA<br>(-2 Da) AA**C**SAA**D**AADA*	936.3727<br>934.3571	936.3750<br>934.3545	-2.50<br>2.75
y10<br>y10*	ACSAADAADA<br>(-2 Da) A**C**SAA**D**AADA*	865.3356<br>863.3200	865.3369<br>863.3192	-1.49<br>0.90
y9<br>y9*	CSAADAADA<br>(-2 Da) **C**SAA**D**AADA*	794.2985<br>792.2829	794.2965<br>792.2817	2.53<br>-1.51
y8<br>y8*	SAADAADA<br>(-1 Da) SAA**D**AADA*	691.2893<br>690.2815	691.2872<br>Not Found	3.10<br>N/A
y7<br>y7*	AADAADA<br>(-1 Da) AA**D**AADA*	604.2573<br>603.2495	604.2559<br>Not Found	2.37<br>N/A
y6<br>y6*	ADAADA<br>(-1 Da) A**D**AADA*	533.2202<br>532.2124	533.2191<br>Not Found	2.12<br>N/A
y5<br>y5*	DAADA<br>**D**AADA*	462.1831<br>461.1752	462.1835<br>Not Found	-0.92<br>N/A
y4<br>y4*	AADA<br>AADA*	347.1561<br>347.1561	347.1567<br>347.1554	-1.82<br>2.07
y3<br>y3*	ADA<br>ADA*	276.1190<br>276.1190	276.1188<br>276.1189	0.67<br>0.18
y2<br>y2*	DA<br>DA*	205.0819<br>205.0819	205.0819<br>205.0820	-0.05<br>-0.49

Figure S25. Iodoacetic acid treatment of unmodified and modified Leader-AAACSANDA peptides
Leader = LKQINVIAGVKEPIRAYG

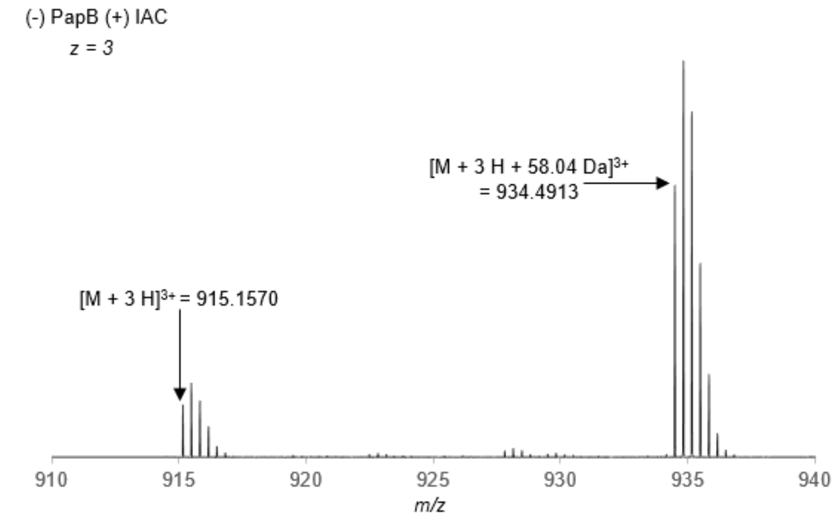

Sequence	Expected Monoisotopic Mass	Observed Monoisotopic Mass	Ppm Error
Leader-AAACSANDA	(-) PapB: 915.1568	915.1570	0.22
Leader-AAACSANDA + S-carboxymethyl	(-) PapB + (58.04 Da): 934.4920	934.4913	-0.75

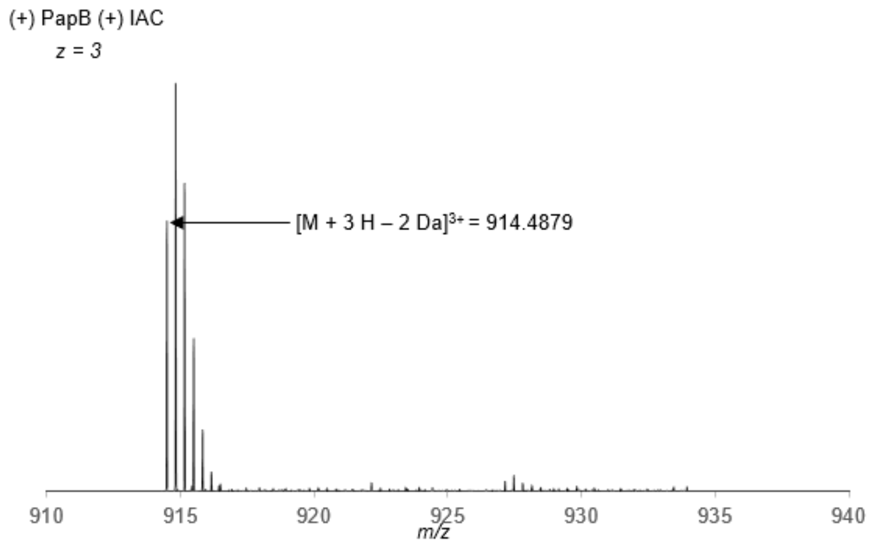

Sequence	Expected Monoisotopic Mass	Observed Monoisotopic Mass	Ppm Error
Leader-AAACSANDA	(+) PapB - (2 Da): 914.4850	914.4854	0.43
Leader-AAACSANDA + S-carboxymethyl	(-) PapB + (58.04 Da): 934.4920	Not Found	N/A

Figure S26. Iodoacetic acid treatment of unmodified and modified Leader-AAACSANDACSANDA peptides
Leader = LKQINVIAGVKEPIRAYG

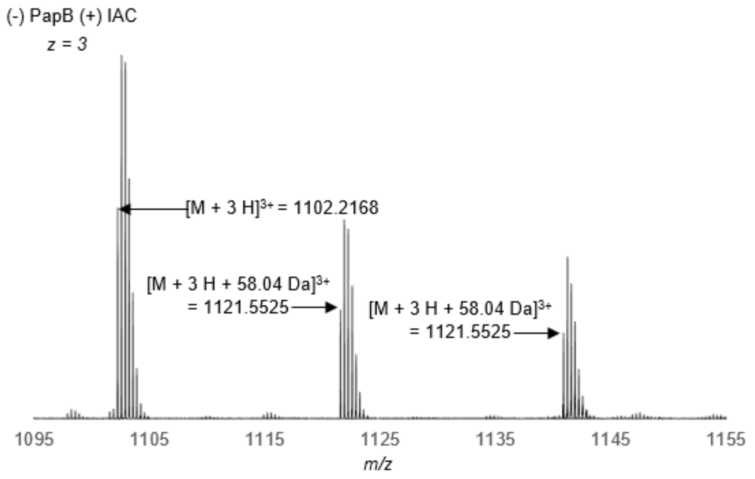

Sequence	Expected Monoisotopic Mass	Observed Monoisotopic Mass	Ppm Error
Leader-AAACSANDACSANDA	(-) PapB: 1102.2186	1102.2168	-1.63
Leader-AAACSANDACSANDA + S-carboxymethyl	(-) PapB + (58.04 Da): 1121.5538	1121.5525	-1.16
Leader-AAACSANDACSANDA + (2x) S-carboxymethyl	(-) PapB + (58.04 Da) + (58.04 Da): 1140.8889	1140.8914	2.19

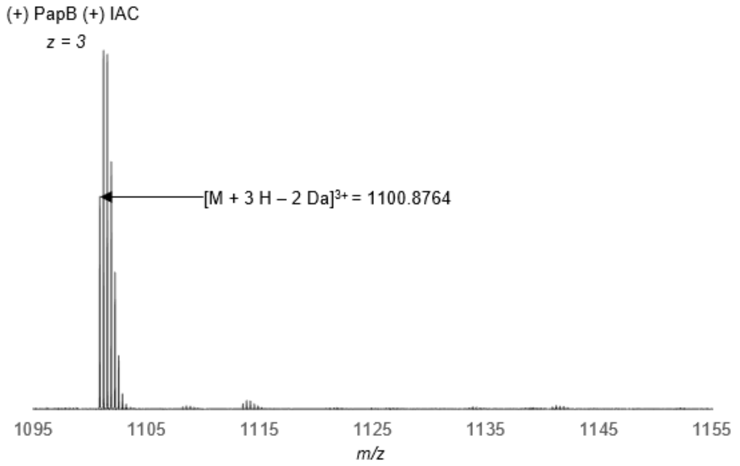

Sequence	Expected Monoisotopic Mass	Observed Monoisotopic Mass	Ppm Error
Leader-AAACSANDACSANDA	(+) PapB - (4 Da): 1100.8748	1100.8764	1.53
Leader-AAACSANDACSANDA + S-carboxymethyl	(-) PapB + (58.04 Da): 1121.5538	Not Found	N/A
Leader-AAACSANDACSANDA + (2x) S-carboxymethyl	(-) PapB + (58.04 Da) + (58.04 Da): 1140.8889	Not Found	N/A

Figure S27. Iodoacetic acid treatment of unmodified and modified Leader-AAACSACDAADA peptides
Leader = LKQINVIAGVKEPIRAYG

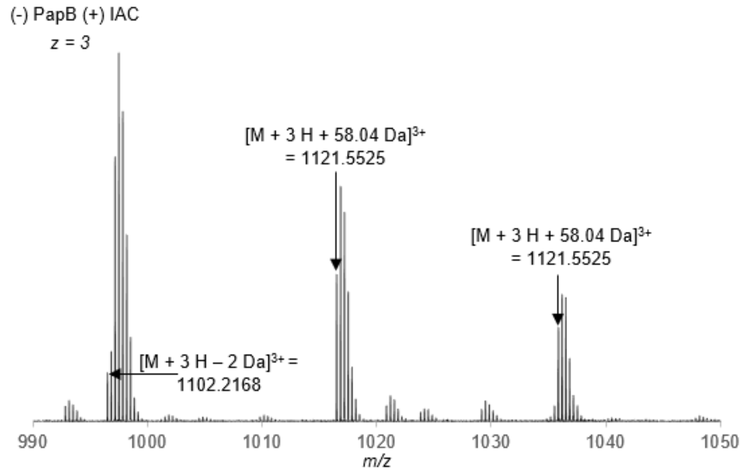

Sequence	Expected Monoisotopic Mass	Observed Monoisotopic Mass	Ppm Error
Leader-AAACSACDAADA	(-) PapB – (2 Da; 1xDisulfide): 996.5074	996.5104	3.01
Leader-AAACSACDAADA + S-carboxymethyl	(-) PapB + (58.04 Da): 1016.5145	1016.5126	-1.87
Leader-AAACSACDAADA + (2x) S-carboxymethyl	(-) PapB + (58.04 Da) + (58.04 Da): 1035.8496	1035.8492	-0.39

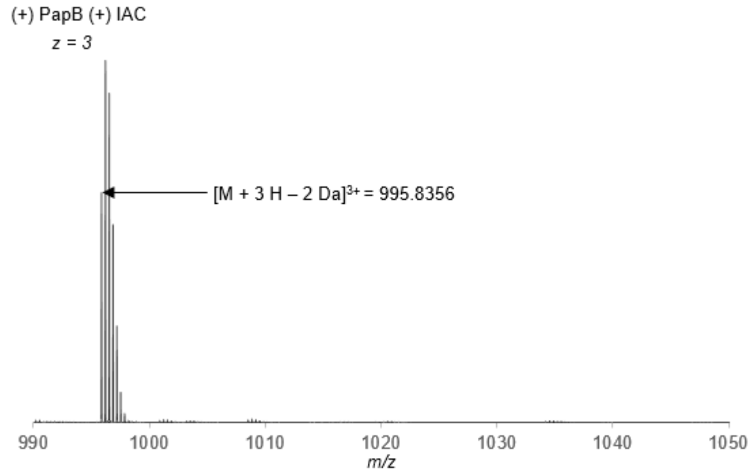

Sequence	Expected Monoisotopic Mass	Observed Monoisotopic Mass	Ppm Error
Leader-AAACSACDAADA	(+) PapB - (4 Da): 995.8355	995.8356	0.10
Leader-AAACSACDAADA + S-carboxymethyl	(-) PapB + (58.04 Da): 1016.5145	Not Found	N/A
Leader-AAACSACDAADA + (2x) S-carboxymethyl	(-) PapB + (58.04 Da) + (58.04 Da): 1035.8496	Not Found	N/A

Figure S28. Iodoacetic acid treatment of unmodified and modified Leader-AAAASACDAADA peptides
Leader = LKQINVIAGVKEPIRAYG

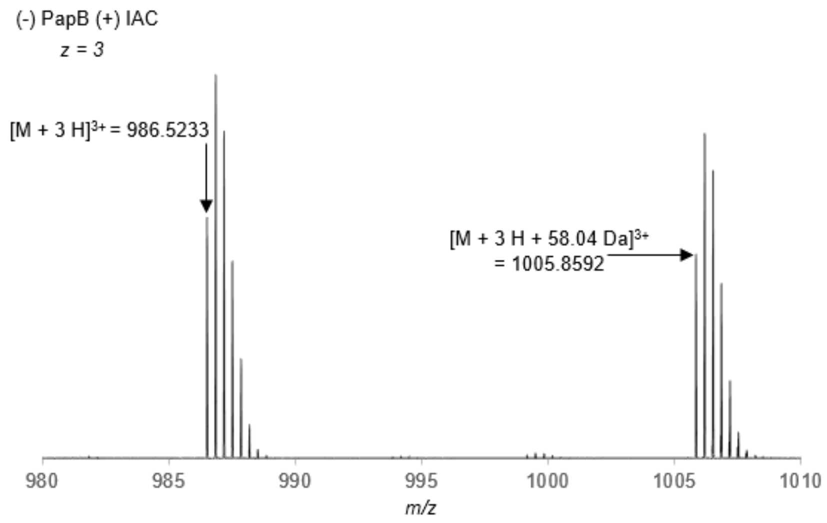

Sequence	Expected Monoisotopic Mass	Observed Monoisotopic Mass	Ppm Error
Leader-AAAASACDAADA	(-) PapB: 986.5220	986.5233	1.32
Leader-AAAASACDAADA + S-carboxymethyl	(-) PapB + (58.04 Da): 1005.8571	1005.8592	2.08

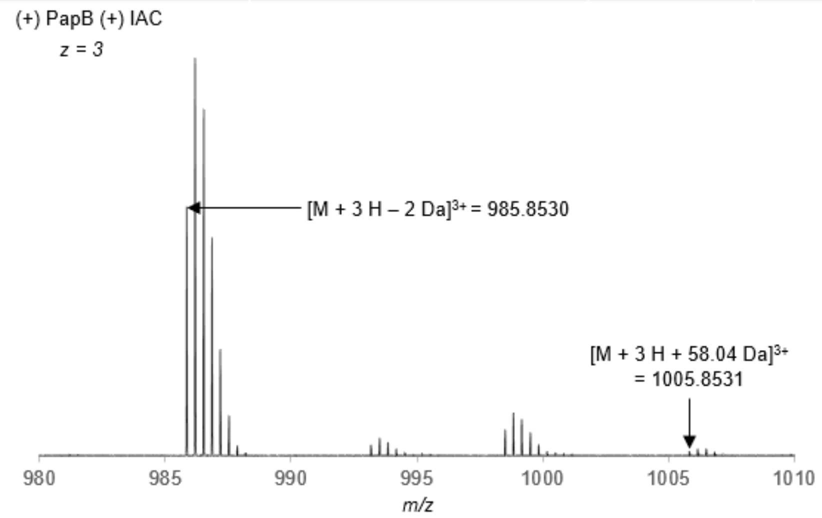

Sequence	Expected Monoisotopic Mass	Observed Monoisotopic Mass	Ppm Error
Leader-AAAASACDAADA	(+) PapB - (2 Da): 985.8501	985.8530	2.94
Leader-AAAASACDAADA + S-carboxymethyl	(-) PapB + (58.04 Da): 1005.8571	1005.8531	-3.98

Figure S29. Iodoacetic acid treatment of unmodified and modified Leader-AAACSAADAADA peptides
Leader = LKQINVIAGVKEPIRAYG

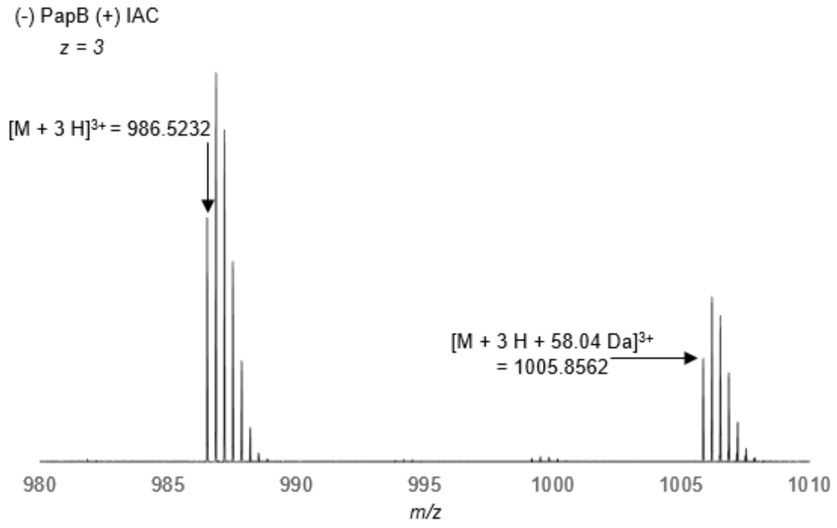

Sequence	Expected Monoisotopic Mass	Observed Monoisotopic Mass	Ppm Error
Leader-AAACSAA**D**AADA	(-) PapB: 986.5220	986.5232	1.22
Leader-AAACSAA**D**AADA + S-carboxymethyl	(-) PapB + (58.04 Da): 1005.8571	1005.8562	-0.89

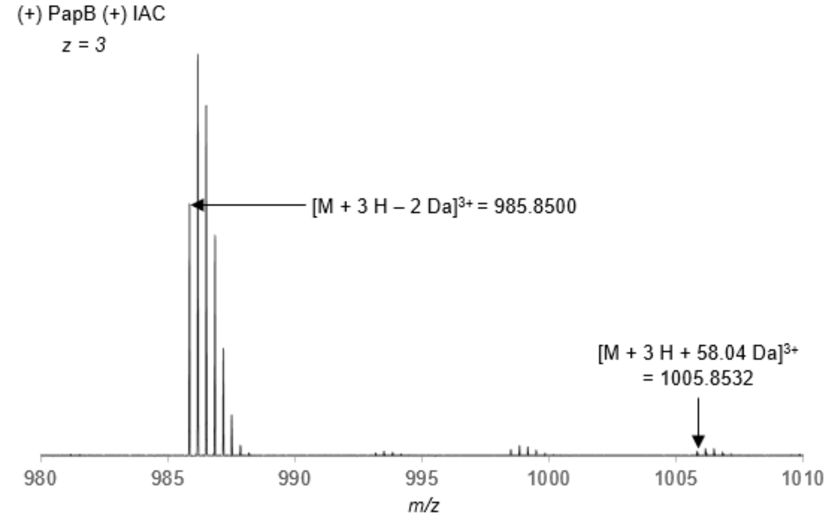

Sequence	Expected Monoisotopic Mass	Observed Monoisotopic Mass	Ppm Error
Leader-AAACSAADAADA	(+) PapB - (2 Da): 985.8501	985.8500	-0.10
Leader-AAACSAADAADA + S-carboxymethyl	(-) PapB + (58.04 Da): 1005.8571	1005.8532	-3.87

Table S30: Expected and observed monoisotopic masses of AMK-1057 and related peptides

Sequence	Expected Monoisotopic Mass	Observed Monoisotopic Mass	Ppm Error
Leader-ENLYFQGV**C**Y KG**E**W**C**EIV**E**I (z = 3)	(-) PapB: 1439.0813<br>(+) PapB (-4Da): 1437.7375	1439.0844<br>1437.7386	2.154<br>0.765
GV**C**YKG**E**W**C**EIV**E**I (z = 2)	(-) PapB: 814.3774<br>(+) PapB (-4Da): 812.3627	814.3784<br>812.3649	1.228<br>2.708

Figure S31. AMK-1057 *in vitro* modification Tandem Mass Spectrometry
Sequence: GV**C**YKG**E**W**C**EIV**E**I

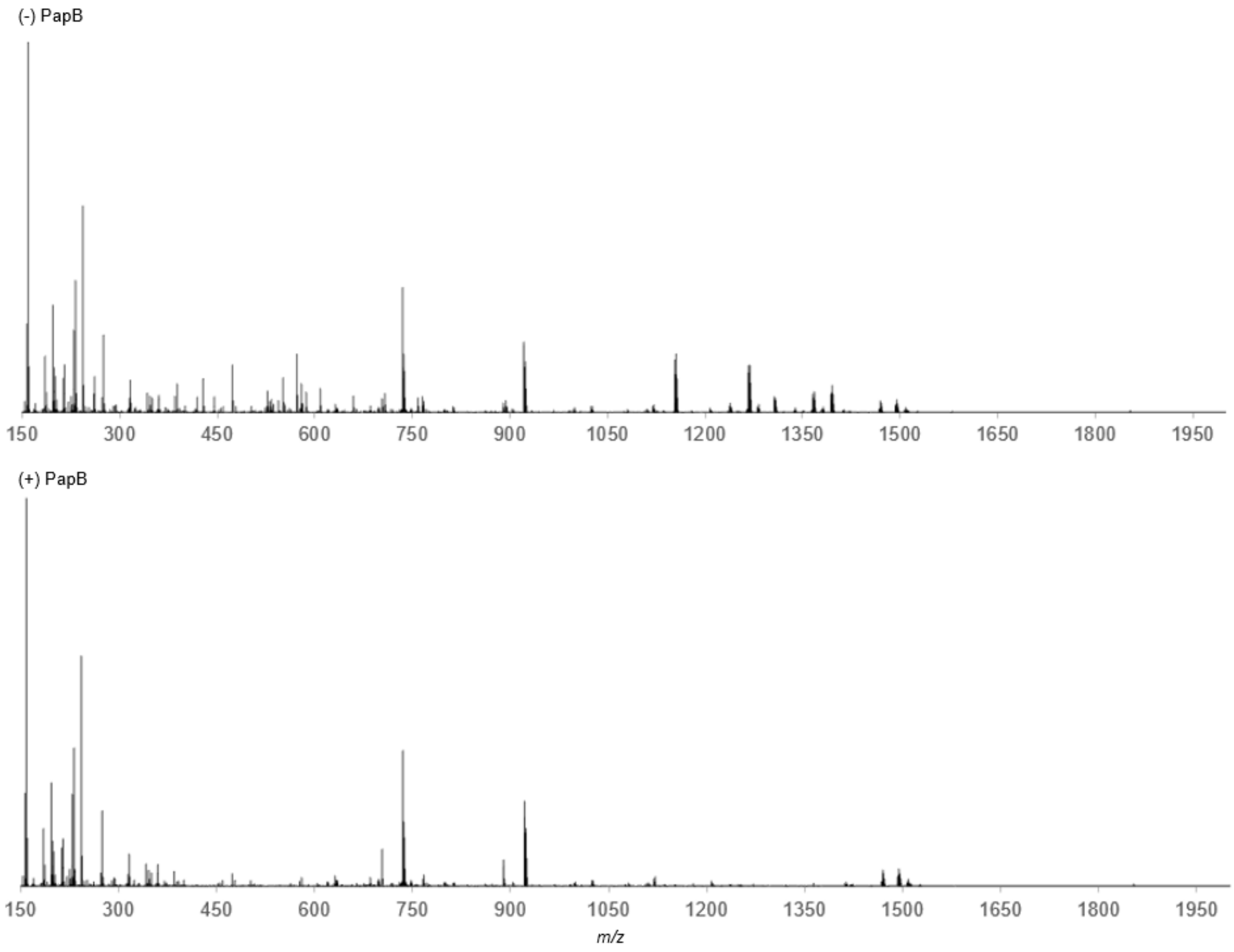

z = 1 charge state in all cases
* Indicates the fragment originated from the modified peptide.

Ion	Sequence	Expected Monoisotopic Mass	Observed Monoisotopic Mass	Ppm Error
b2<br>b2*	GV<br>GV*	157.0972<br>157.0972	157.0972<br>157.0972	-0.01<br>-0.03
b3<br>b3*	GVC<br>GV**C***	260.1063<br>259.0985	260.1058<br>Not Found	1.75<br>N/A
b4<br>b4*	GVCK<br>GV**C**K*	388.2013<br>387.1935	388.2022<br>Not Found	-2.29<br>N/A
b5<br>b5*	GVCKY<br>GV**C**KY*	551.2646<br>550.2568	551.2648<br>Not Found	-0.35<br>N/A
b6<br>b6*	GVCKYG<br>GV**C**KYG*	608.2861<br>607.2783	608.2868<br>Not Found	-1.12<br>N/A
b7<br>b7*	GVCKYGE<br>GV**C**KYG**E***	737.3287<br>735.3130	737.3305<br>735.3131	-2.40<br>-0.07
b8<br>b8*	GVCKYGEW<br>GV**C**KYG**E**W*	923.4080<br>921.3923	923.4054<br>921.3938	2.84<br>-1.63
b9<br>b9*	GVCKYGEWC<br>GV**C**KYG**E**W**C***	1026.4172<br>1023.3937	1026.4167<br>Not Found	0.53<br>N/A
b10<br>b10*	GVCKYGEWCE<br>GV**C**KYG**E**W**C**E*	1155.4598<br>1152.4363	1155.4610<br>Not Found	-1.03<br>N/A
b11<br>b11*	GVCKYGEWCEI<br>GV**C**KYG**E**W**C**EI*	1268.5438<br>1265.5204	1268.5428<br>Not Found	0.75<br>N/A
b12<br>b12*	GVCKYGEWCEIV<br>GV**C**KYG**E**W**C**EIV*	1367.6123<br>1364.5888	1367.6174<br>Not Found	-3.71<br>N/A
b13<br>b13*	GVCKYGEWCEIVE<br>GV**C**KYG**E**W**C**EIV**E***	1496.6548<br>1492.6235	1496.6590<br>1492.6197	-2.79<br>2.57
[M]<br>[M]*	GVCKYGEWCEIVEI<br>GV**C**KYG**E**W**C**EIV**E**I*	1627.7495<br>1623.7182	1627.7434<br>1623.7208	3.72<br>-1.58
y13<br>y13*	VCKYGEWCEIVEI<br>V**C**KYG**E**W**C**EIV**E**I*	1570.7280<br>1566.6967	1570.7260<br>Not Found	1.26<br>N/A
y12<br>y12*	CKYGEWCEIVEI<br>**C**KYG**E**W**C**EIV**E**I*	1471.6596<br>1467.6283	1471.6622<br>1467.6302	-1.73<br>-1.31
y11<br>y11*	KYGEWCEIVEI<br>KYG**E**W**C**EIV**E**I*	1368.6504<br>1365.6269	1368.6492<br>Not Found	0.87<br>N/A
y10<br>y10*	YGEWCEIVEI<br>YG**E**W**C**EIV**E**I*	1240.5555<br>1237.5320	1240.5524<br>Not Found	2.50<br>N/A
y9<br>y9*	GEWCEIVEI<br>G**E**W**C**EIV**E**I*	1077.4921<br>1074.4686	1077.4904<br>Not Found	1.57<br>N/A
y8<br>y8*	EWCEIVEI<br>**E**W**C**EIV**E**I*	1020.4707<br>1017.4472	1020.4704<br>Not Found	0.29<br>N/A
y7<br>y7*	WCEIVEI<br>W**C**EIV**E**I*	891.4281<br>889.4124	891.4287<br>889.4137	-0.64<br>-1.44
y6<br>y6*	CEIVEI<br>**C**EIV**E**I*	705.3488<br>703.3331	705.3476<br>703.3317	1.64<br>2.01

y5	EIVEI	602.3396	602.3408	-1.94
y5*	EIV**E**I*	601.3317	Not Found	N/A
y4	IVEI	473.2970	473.2968	0.49
y4*	IV**E**I*	472.2892	Not Found	N/A
y3	VEI	360.2129	360.2135	-1.77
y3*	V**E**I*	359.2051	Not Found	N/A
y2	EI	261.1445	261.1443	0.72
y2*	**E**I*	260.1367	Not Found	N/A

Figure S32: PapB Crosslinks ^{D}C and ^{D}D msPapA Peptides.

The data for Leader-^{L}CSAN^{L}DA is shown in **Fig. S1**.

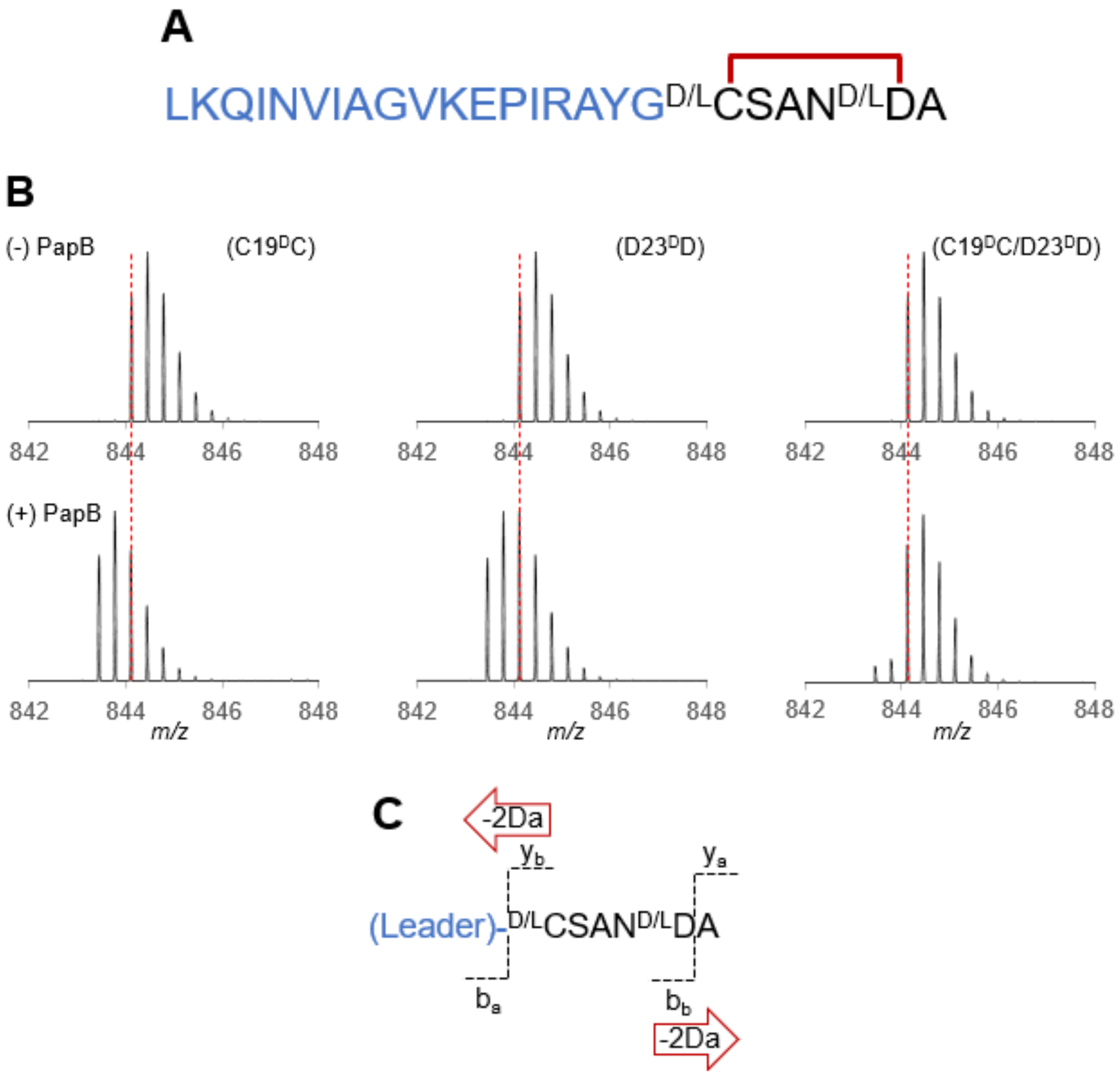

(A) Sequence and topology of D-variants, (B) C19^{D}C appears to go to completion whereas D23D^{D} and the C19^{D}C/D23^{D}D variants are not processed as efficiently. (C) The tandem mass spectrometry results (see **Fig. S33-S35**) are consistent with those of the L-amino acid containing peptide, with each *y*-fragment after C containing a 2 Da loss and each *b*-fragment after the D containing a 2 Da loss.

Figure S33. Tandem Mass Spectrometry of msPapA C19^{D}C variant

Sequence: LKQINVIAGVKEPIRAYG^{D}**C**SAN**D**A

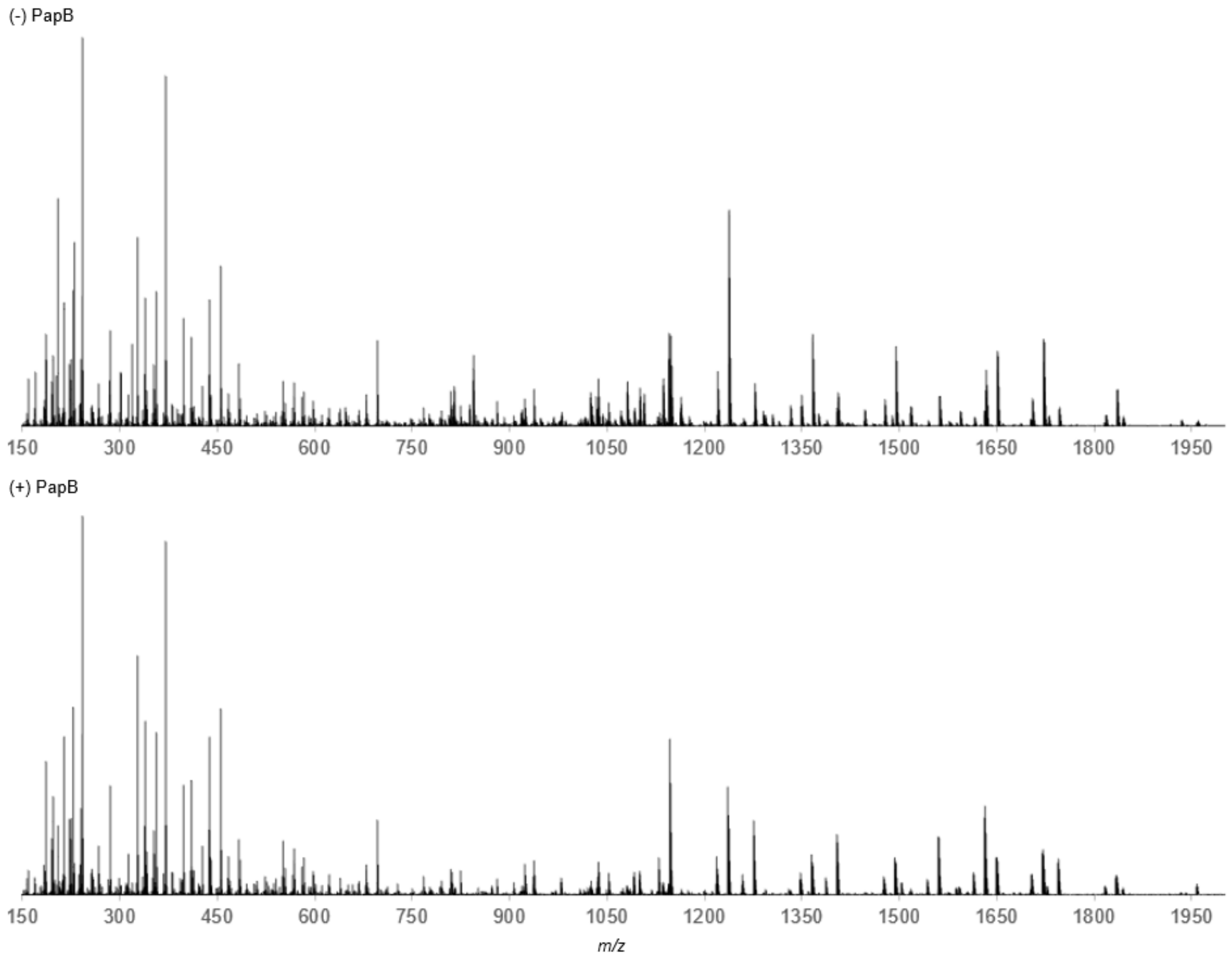

Unhighlighted indicates *z* = 1 charge state.
Gold highlight indicates *z* = 2 charge state.
Teal highlight indicates *z* = 3 charge state.
* Indicates the fragment originated from the modified peptide.

Ion	Sequence	Expected Monoisotopic Mass	Observed Monoisotopic Mass	Ppm Error
b2	LK	242.1863	242.1864	-0.27
b2*	LK*	242.1863	242.1866	-1.33
b3	LKQ	370.2449	370.2447	0.52
b3*	LKQ*	370.2449	370.2450	-0.27
b4	LKQI	483.3289	483.3292	-0.72
b4*	LKQI*	483.3289	483.3295	-1.32
b5	LKQIN	597.3719	597.3712	1.09

b5*	LKQIN*	597.3719	597.3715	0.67
b6	LKQINV	696.4403	696.4404	-0.14
b6*	LKQINV*	696.4403	696.4406	-0.43
b7	LKQINVI	809.5244	809.5226	2.16
b7*	LKQINVI*	809.5244	809.5228	2.03
b8	LKQINVIA	880.5615	880.5638	-2.63
b8*	LKQINVIA*	880.5615	880.5639	-2.68
b9	LKQINVIAG	937.5829	937.5797	3.41
b9*	LKQINVIAG*	937.5829	937.5797	3.43
b10	LKQINVIAGV	1036.6513	1036.6543	-2.89
b10*	LKQINVIAGV*	1036.6513	1036.6542	-2.75
b11	LKQINVIAGVK	1164.7463	1164.7483	-1.72
b11*	LKQINVIAGVK*	1164.7463	1164.7480	-1.44
b12	LKQINVIAGVKE	1293.7889	1293.7912	-1.75
b12*	LKQINVIAGVKE*	1293.7889	1293.7906	-1.34
b13	LKQINVIAGVKEP	1390.8417	1390.8408	0.64
b13*	LKQINVIAGVKEP*	1390.8417	1390.8401	1.15
b14	LKQINVIAGVKEPI	1503.9257	1503.9250	0.50
b14*	LKQINVIAGVKEPI*	1503.9257	1503.9240	1.12
b15	LKQINVIAGVKEPIR	1660.0268	1660.0322	-3.23
b15*	LKQINVIAGVKEPIR*	1660.0268	Not Found	N/A
b16	LKQINVIAGVKEPIRA	866.0356	866.0384	-3.24
b16*	LKQINVIAGVKEPIRA*	866.0356	866.0385	-3.31
b17	LKQINVIAGVKEPIRAY	947.5673	947.5693	-2.11
b17*	LKQINVIAGVKEPIRAY*	947.5673	947.5693	-2.08
b18	LKQINVIAGVKEPIRAYG	976.0780	976.0812	-3.27
b18*	LKQINVIAGVKEPIRAYG*	976.0780	976.0811	-3.20
b19	LKQINVIAGVKEPIRAYG^{D}C	1027.5826	1027.5820	0.56
b19*	(-1 Da) LKQINVIAGVKEPIRAYG^{D}**C***	1027.0787	Not Found	N/A
b20	LKQINVIAGVKEPIRAYG^{D}CS	1071.0986	1071.0984	0.16
b20*	(-1 Da) LKQINVIAGVKEPIRAYG^{D}**C**S*	1070.5947	Not Found	N/A
b21	LKQINVIAGVKEPIRAYG^{D}CSA	1106.6172	1106.6158	1.23
b21*	(-1 Da) LKQINVIAGVKEPIRAYG^{D}**C**SA*	1106.1133	Not Found	N/A
b22	LKQINVIAGVKEPIRAYG^{D}CSAN	776.0948	776.0930	2.26
b22*	(-1 Da) LKQINVIAGVKEPIRAYG^{D}**C**SAN*	775.7589	Not Found	N/A
b23	LKQINVIAGVKEPIRAYG^{D}CSAND	814.4372	814.4379	-0.84
b23*	(-2 Da) LKQINVIAGVKEPIRAYG^{D}**C**SAN**D***	813.7653	813.7661	-0.98
[M]	LKQINVIAGVKEPIRAYG^{D}CSANDA	844.1197	844.1179	2.19
[M]*	(-2 Da) LKQINVIAGVKEPIRAYG^{D}**C**SAN**D**A*	843.4478	843.4496	-2.18
y23	KQINVIAGVKEPIRAYG^{D}CSANDA	806.4250	806.4243	0.84
y23*	(-2 Da) KQINVIAGVKEPIRAYG^{D}**C**SAN**D**A*	805.7532	805.7516	2.00
y22	QINVIAGVKEPIRAYG^{D}CSANDA	763.7267	763.7248	2.46
y22*	(-2 Da) QINVIAGVKEPIRAYG^{D}**C**SAN**D**A*	763.0548	763.0549	-0.08
y21	INVIAGVKEPIRAYG^{D}CSANDA	721.0405	721.0402	0.40

y21*	(-2 Da) INVIAGVKEPIRAYG^{D}**C**SAN**D**A*	720.3686	720.3706	-2.84
y20<br>y20*	NVIAGVKEPIRAYG^{D}CSANDA<br>(-2 Da) NVIAGVKEPIRAYG^{D}**C**SAN**D**A*	1024.5151<br>1023.5073	1024.5118<br>1023.5095	3.20<br>-2.12
y19<br>y19*	VIAGVKEPIRAYG^{D}CSANDA<br>(-2 Da) VIAGVKEPIRAYG^{D}**C**SAN**D**A*	967.4936<br>966.4858	967.4910<br>966.4857	2.69<br>0.10
y18<br>y18*	IAGVKEPIRAYG^{D}CSANDA<br>(-2 Da) IAGVKEPIRAYG^{D}**C**SAN**D**A*	917.9594<br>916.9516	917.9565<br>916.9485	3.11<br>3.39
y17<br>y17*	AGVKEPIRAYG^{D}CSANDA<br>(-2 Da) AGVKEPIRAYG^{D}**C**SAN**D**A*	861.4174<br>860.4096	861.4162<br>860.4101	1.34<br>-0.64
y16<br>y16*	GVKEPIRAYG^{D}CSANDA<br>(-2 Da) GVKEPIRAYG^{D}**C**SAN**D**A*	1650.7904<br>1648.7748	1650.7861<br>1648.7671	2.61<br>4.66
y15<br>y15*	VKEPIRAYG^{D}CSANDA<br>(-2 Da) VKEPIRAYG^{D}**C**SAN**D**A*	1593.7690<br>1591.7533	1593.7707<br>1591.7500	-1.04<br>2.08
y14<br>y14*	KEPIRAYG^{D}CSANDA<br>(-2 Da) KEPIRAYG^{D}**C**SAN**D**A*	1494.7006<br>1492.6849	1494.7028<br>1492.6898	-1.46<br>-3.31
y13<br>y13*	EPIRAYG^{D}CSANDA<br>(-2 Da) EPIRAYG^{D}**C**SAN**D**A*	1366.6056<br>1364.5899	1366.6030<br>1364.5922	1.93<br>-1.70
y12<br>y12*	PIRAYG^{D}CSANDA<br>(-2 Da) PIRAYG^{D}**C**SAN**D**A*	1237.5630<br>1235.5473	1237.5675<br>1235.5464	-3.66<br>0.72
y11<br>y11*	IRAYG^{D}CSANDA<br>(-2 Da) IRAYG^{D}**C**SAN**D**A*	1140.5102<br>1138.4946	1140.5066<br>1138.4908	3.20<br>3.37
y10<br>y10*	RAYG^{D}CSANDA<br>(-2 Da) RAYG^{D}**C**SAN**D**A*	1027.4262<br>1025.4105	1027.4258<br>1025.4142	0.34<br>-3.59
y9<br>y9*	AYG^{D}CSANDA<br>(-2 Da) AYG^{D}**C**SAN**D**A*	871.3251<br>869.3094	871.3256<br>869.3107	-0.60<br>-1.53
y8<br>y8*	YG^{D}CSANDA<br>(-2 Da) YG^{D}**C**SAN**D**A*	800.2879<br>798.2723	800.2862<br>798.2718	2.18<br>0.58
y7<br>y7*	G^{D}CSANDA<br>(-2 Da) G^{D}**C**SAN**D**A*	637.2246<br>635.2090	637.2248<br>635.2085	-0.32<br>0.75
y6<br>y6*	^{D}CSANDA<br>(-2 Da) ^{D}**C**SAN**D**A*	580.2032<br>578.1875	580.2032<br>578.1874	0.07<br>0.10
y5<br>y5*	SANDA<br>(-1 Da) SAN**D**A*	477.1940<br>476.1861	477.1933<br>Not Found	1.52<br>N/A
y4<br>y4*	ANDA<br>(-1 Da) AN**D**A*	390.1619<br>389.1541	390.1614<br>Not Found	1.40<br>N/A
y3<br>y3*	NDA<br>(-1 Da) N**D**A*	319.1248<br>318.1170	319.1250<br>Not Found	-0.63<br>N/A
y2<br>y2*	DA<br>(-1 Da) **D**A*	205.0819<br>204.0741	205.0822<br>Not Found	-1.53<br>N/A

Figure S34. Tandem Mass Spectrometry of msPapA D23^{D}D variant Sequence: LKQINVIAGVKEPIRAYG**C**SAN^{D}**D**A

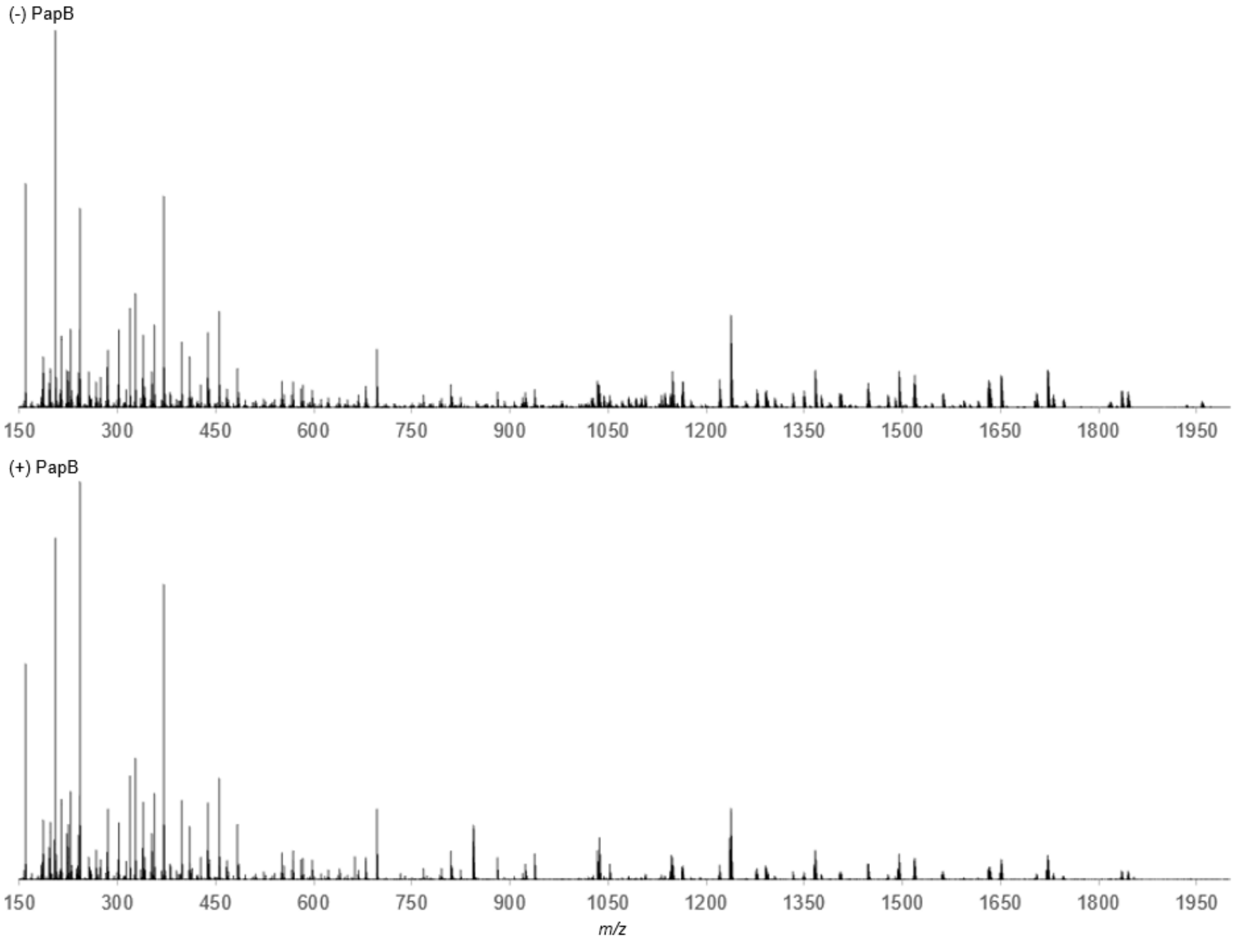

Unhighlighted indicates *z* = 1 charge state.
Gold highlight indicates *z* = 2 charge state.
Teal highlight indicates *z* = 3 charge state.
* Indicates the fragment originated from the modified peptide.

Ion	Sequence	Expected Monoisotopic Mass	Observed Monoisotopic Mass	Ppm Error
b2	LK	242.1863	242.1866	-1.06
b2*	LK*	242.1863	242.1860	1.20
b3	LKQ	370.2449	370.2452	-0.85
b3*	LKQ*	370.2449	370.2456	-1.85
b4	LKQI	483.3289	483.3301	-2.53
b4*	LKQI*	483.3289	483.3279	2.02
b5	LKQIN	597.3719	597.3726	-1.11
b5*	LKQIN*	597.3719	597.3728	-1.48

b6<br>b6*	LKQINV<br>LKQINV*	696.4403<br>696.4403	696.4422<br>696.4422	-2.66<br>-2.79
b7<br>b7*	LKQINVI<br>LKQINVI*	809.5244<br>809.5244	809.5250<br>809.5249	-0.68<br>-0.56
b8<br>b8*	LKQINVIA<br>LKQINVIA*	880.5615<br>880.5615	880.5603<br>880.5601	1.36<br>1.63
b9<br>b9*	LKQINVIAG<br>LKQINVIAG*	937.5829<br>937.5829	937.5827<br>937.5823	0.21<br>0.60
b10<br>b10*	LKQINVIAGV<br>LKQINVIAGV*	1036.6513<br>1036.6513	1036.6500<br>1036.6494	1.30<br>1.88
b11<br>b11*	LKQINVIAGVK<br>LKQINVIAGVK*	1164.7463<br>1164.7463	1164.7433<br>1164.7423	2.61<br>3.43
b12<br>b12*	LKQINVIAGVKE<br>LKQINVIAGVKE*	1293.7889<br>1293.7889	1293.7854<br>1293.7840	2.73<br>3.77
b13<br>b13*	LKQINVIAGVKEP<br>LKQINVIAGVKEP*	1390.8417<br>1390.8417	1390.8467<br>1390.8451	-3.62<br>-2.41
b14<br>b14*	LKQINVIAGVKEPI<br>LKQINVIAGVKEPI*	1503.9257<br>1503.9257	1503.9317<br>1503.9296	-4.00<br>-2.61
b15<br>b15*	LKQINVIAGVKEPIR<br>LKQINVIAGVKEPIR*	1660.0268<br>1660.0268	1660.0241<br>Not Found	1.61<br>N/A
b16<br>b16*	LKQINVIAGVKEPIRA<br>LKQINVIAGVKEPIRA*	866.0356<br>866.0356	866.0350<br>Not Found	0.73<br>N/A
b17<br>b17*	LKQINVIAGVKEPIRAY<br>LKQINVIAGVKEPIRAY*	947.5673<br>947.5673	947.5654<br>947.5651	1.96<br>2.37
b18<br>b18*	LKQINVIAGVKEPIRAYG<br>LKQINVIAGVKEPIRAYG*	976.0780<br>976.0780	976.0772<br>976.0767	0.85<br>1.31
b19<br>b19*	LKQINVIAGVKEPIRAYGC<br>(-1 Da) LKQINVIAGVKEPIRAYGC*	1027.5826<br>1027.0787	1027.5855<br>Not Found	-2.87<br>N/A
b20<br>b20*	LKQINVIAGVKEPIRAYGCS<br>(-1 Da) LKQINVIAGVKEPIRAYGCS*	1071.0986<br>1070.5947	1071.1022<br>Not Found	-3.37<br>N/A
b21<br>b21*	LKQINVIAGVKEPIRAYGCSA<br>(-1 Da) LKQINVIAGVKEPIRAYGCSA*	1106.6172<br>1106.1133	1106.6198<br>Not Found	-2.38<br>N/A
b22<br>b22*	LKQINVIAGVKEPIRAYGCSAN<br>(-1 Da) LKQINVIAGVKEPIRAYGCSAN*	776.0948<br>775.7589	776.0952<br>Not Found	-0.49<br>N/A
b23<br>b23*	LKQINVIAGVKEPIRAYGCSAN^{D}D<br>(-2 Da) LKQINVIAGVKEPIRAYGCSAN^{D}D*	814.4372<br>813.7653	814.4347<br>813.7627	3.07<br>3.19
[M]<br>[M]*	LKQINVIAGVKEPIRAYGCSAN^{D}DA<br>(-2 Da) LKQINVIAGVKEPIRAYGCSAN^{D}DA*	844.1197<br>843.4478	844.1190<br>843.4461	0.83<br>2.05
y23<br>y23*	KQINVIAGVKEPIRAYGCSAN^{D}DA<br>(-2 Da) KQINVIAGVKEPIRAYGCSAN^{D}DA*	806.4250<br>805.7532	806.4266<br>805.7537	-2.00<br>-0.58
y22<br>y22*	QINVIAGVKEPIRAYGCSAN^{D}DA<br>(-2 Da) QINVIAGVKEPIRAYGCSAN^{D}DA*	763.7267<br>763.0548	Not Found<br>763.0568	N/A<br>-2.58
y21<br>y21*	INVIAGVKEPIRAYGCSAN^{D}DA<br>(-2 Da) INVIAGVKEPIRAYGCSAN^{D}DA*	721.0405<br>720.3686	Not Found<br>720.3678	N/A<br>1.11

y20	NVIAGVKEPIRAYGCSAN^{D}DA	1024.5151	1024.5153	-0.22
y20*	(-2 Da) NVIAGVKEPIRAYG**C**SAN$^{\mathbf{D}}$**DA***	1023.5073	1023.5048	2.49
y19	VIAGVKEPIRAYGCSAN^{D}DA	967.4936	967.4942	-0.58
y19*	(-2 Da) VIAGVKEPIRAYG**C**SAN$^{\mathbf{D}}$**DA***	966.4858	966.4885	-2.78
y18	IAGVKEPIRAYGCSAN^{D}DA	917.9594	917.9594	-0.04
y18*	(-2 Da) IAGVKEPIRAYG**C**SAN$^{\mathbf{D}}$**DA***	916.9516	916.9511	0.60
y17	AGVKEPIRAYGCSAN^{D}DA	861.4174	861.4188	-1.65
y17*	(-2 Da) AGVKEPIRAYG**C**SAN$^{\mathbf{D}}$**DA***	860.4096	Not Found	N/A
y16	GVKEPIRAYGCSAN^{D}DA	1650.7904	1650.7940	-2.19
y16*	(-2 Da) GVKEPIRAYG**C**SAN$^{\mathbf{D}}$**DA***	1648.7748	1648.7736	0.74
y15	VKEPIRAYGCSAN^{D}DA	1593.7690	1593.7630	3.74
y15*	(-2 Da) VKEPIRAYG**C**SAN$^{\mathbf{D}}$**DA***	1591.7533	1591.7561	-1.77
y14	KEPIRAYGCSAN^{D}DA	1494.7006	1494.6958	3.23
y14*	(-2 Da) KEPIRAYG**C**SAN$^{\mathbf{D}}$**DA***	1492.6849	1492.6817	2.14
y13	EPIRAYGCSAN^{D}DA	1366.6056	1366.6087	-2.29
y13*	(-2 Da) EPIRAYG**C**SAN$^{\mathbf{D}}$**DA***	1364.5899	1364.5851	3.54
y12	PIRAYGCSAN^{D}DA	1237.5630	1237.5621	0.75
y12*	(-2 Da) PIRAYG**C**SAN$^{\mathbf{D}}$**DA***	1235.5473	1235.5505	-2.61
y11	IRAYGCSAN^{D}DA	1140.5102	1140.5108	-0.50
y11*	(-2 Da) IRAYG**C**SAN$^{\mathbf{D}}$**DA***	1138.4946	1138.4944	0.20
y10	RAYGCSAN^{D}DA	1027.4262	1027.4294	-3.08
y10*	(-2 Da) RAYG**C**SAN$^{\mathbf{D}}$**DA***	1025.4105	1025.4095	1.02
y9	AYGCSAN^{D}DA	871.3251	871.3222	3.38
y9*	(-2 Da) AYG**C**SAN$^{\mathbf{D}}$**DA***	869.3094	869.3070	2.76
y8	YGCSAN^{D}DA	800.2879	800.2884	-0.65
y8*	(-2 Da) YG**C**SAN$^{\mathbf{D}}$**DA***	798.2723	798.2739	-1.99
y7	GCSAN^{D}DA	637.2246	637.2263	-2.66
y7*	(-2 Da) G**C**SAN$^{\mathbf{D}}$**DA***	635.2090	635.2099	-1.48
y6	CSAN^{D}DA	580.2032	580.2044	-2.08
y6*	(-2 Da) **C**SAN$^{\mathbf{D}}$**DA***	578.1875	578.1887	-2.00
y5	SAN^{D}DA	477.1940	477.1941	-0.27
y5*	(-1 Da) SAN$^{\mathbf{D}}$**DA***	476.1861	Not Found	N/A
y4	AN^{D}DA	390.1619	390.1619	-0.06
y4*	(-1 Da) AN$^{\mathbf{D}}$**DA***	389.1541	Not Found	N/A
y3	N^{D}DA	319.1248	319.1254	-1.78
y3*	(-1 Da) N$^{\mathbf{D}}$**DA***	318.1170	Not Found	N/A
y2	^{D}DA	205.0819	205.0816	1.28
y2*	(-1 Da) $^{\mathbf{D}}$**DA***	204.0741	204.0739	0.79

Figure S35. Tandem Mass Spectrometry of msPapA C19^{D}C/D23^{D}D variant
Sequence: LKQINVIAGVKEPIRAYG^{D}**C**SAN^{D}**D**A

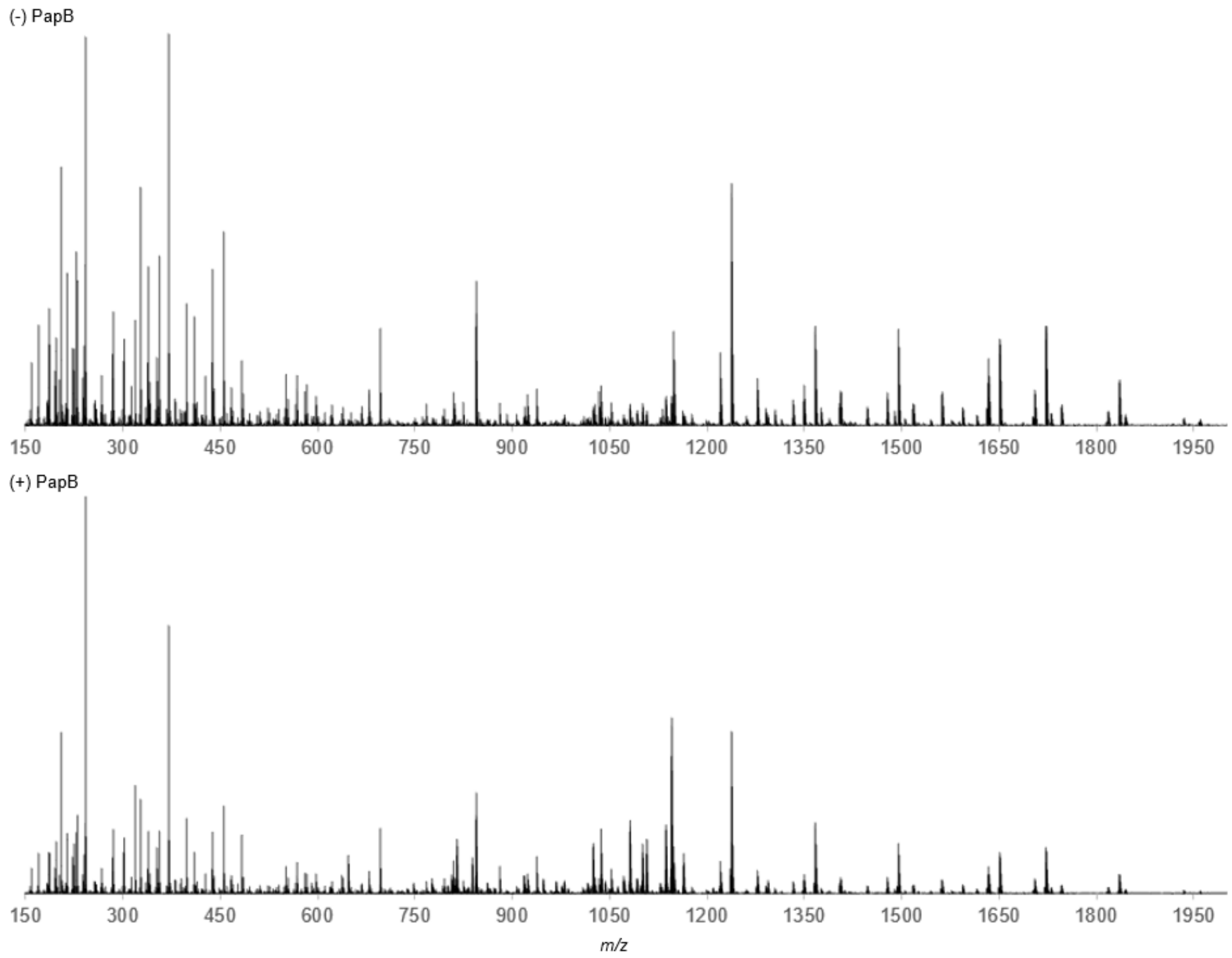

Unhighlighted indicates z = 1 charge state.
Gold highlight indicates z = 2 charge state.
Teal highlight indicates z = 3 charge state.
* Indicates the fragment originated from the modified peptide.

Ion	Sequence	Expected Monoisotopic Mass	Observed Monoisotopic Mass	Ppm Error
b2	LK	242.1863	242.1865	-0.83
b2*	LK*	242.1863	242.1867	-1.51
b3	LKQ	370.2449	370.2450	-0.40
b3*	LKQ*	370.2449	370.2455	-1.67
b4	LKQI	483.3289	483.3298	-1.91
b4*	LKQI*	483.3289	483.3281	1.59
b5	LKQIN	597.3719	597.3721	-0.34
b5*	LKQIN*	597.3719	597.3734	-2.45
b6	LKQINV	696.4403	696.4415	-1.76

b6*	LKQINV*	696.4403	696.4389	2.07
b7	LKQINVI	809.5244	809.5241	0.34
b7*	LKQINVI*	809.5244	809.5263	-2.41
b8	LKQINVIA	880.5615	880.5593	2.46
b8*	LKQINVIA*	880.5615	880.5619	-0.48
b9	LKQINVIAG	937.5829	937.5816	1.37
b9*	LKQINVIAG*	937.5829	937.5845	-1.72
b10	LKQINVIAGV	1036.6513	1036.6486	2.56
b10*	LKQINVIAGV*	1036.6513	1036.6521	-0.78
b11	LKQINVIAGVK	1164.7463	1164.7416	3.99
b11*	LKQINVIAGVK*	1164.7463	1164.7459	0.34
b12	LKQINVIAGVKE	1293.7889	1293.7834	4.22
b12*	LKQINVIAGVKE*	1293.7889	1293.7885	0.28
b13	LKQINVIAGVKEP	1390.8417	Not Found	N/A
b13*	LKQINVIAGVKEP*	1390.8417	1390.8380	2.65
b14	LKQINVIAGVKEPI	1503.9257	1503.9292	-2.33
b14*	LKQINVIAGVKEPI*	1503.9257	1503.9220	2.48
b15	LKQINVIAGVKEPIR	1660.0268	1660.0211	3.40
b15*	LKQINVIAGVKEPIR*	1660.0268	Not Found	N/A
b16	LKQINVIAGVKEPIRA	866.0356	866.0340	1.82
b16*	LKQINVIAGVKEPIRA*	866.0356	866.0365	-1.08
b17	LKQINVIAGVKEPIRAY	947.5673	947.5643	3.13
b17*	LKQINVIAGVKEPIRAY*	947.5673	947.5673	0.01
b18	LKQINVIAGVKEPIRAYG	976.0780	976.0760	2.05
b18*	LKQINVIAGVKEPIRAYG*	976.0780	976.0791	-1.15
b19	LKQINVIAGVKEPIRAYG^{D}C	1027.5826	1027.5843	-1.62
b19*	(-1 Da) LKQINVIAGVKEPIRAYG^{D}C*	1027.0787	1027.0802	-1.48
b20	LKQINVIAGVKEPIRAYG^{D}CS	1071.0986	1071.1008	-2.08
b20*	(-1 Da) LKQINVIAGVKEPIRAYG^{D}CS*	1070.5947	1070.5977	-2.79
b21	LKQINVIAGVKEPIRAYG^{D}CSA	1106.6172	1106.6184	-1.06
b21*	(-1 Da) LKQINVIAGVKEPIRAYG^{D}CSA*	1106.1133	1106.1162	-2.62
b22	LKQINVIAGVKEPIRAYG^{D}CSAN	776.0948	776.0944	0.50
b22*	(-1 Da) LKQINVIAGVKEPIRAYG^{D}CSAN*	775.7589	775.7583	0.83
b23	LKQINVIAGVKEPIRAYG^{D}CSAN^{D}D	814.4372	814.4394	-2.67
b23*	(-2 Da) LKQINVIAGVKEPIRAYG^{D}CSAN^{D}D*	813.7653	813.7642	1.32
[M]	LKQINVIAGVKEPIRAYG^{D}CSAN^{D}DA	844.1197	844.1194	0.31
[M]*	(-2 Da) LKQINVIAGVKEPIRAYG^{D}CSAN^{D}DA*	843.4478	843.4477	0.07
y23	KQINVIAGVKEPIRAYG^{D}CSAN^{D}DA	806.4250	806.4258	-0.97
y23*	(-2 Da) KQINVIAGVKEPIRAYG^{D}CSAN^{D}DA*	805.7532	805.7551	-2.41
y22	QINVIAGVKEPIRAYG^{D}CSAN^{D}DA	763.7267	Not Found	N/A
y22*	(-2 Da) QINVIAGVKEPIRAYG^{D}CSAN^{D}DA*	763.0548	763.0530	2.31
y21	INVIAGVKEPIRAYG^{D}CSAN^{D}DA	721.0405	Not Found	N/A
y21*	(-2 Da) INVIAGVKEPIRAYG^{D}CSAN^{D}DA*	720.3686	Not Found	N/A
y20	NVIAGVKEPIRAYG^{D}CSAN^{D}DA	1024.5151	1024.5140	1.03

y20*	(-2 Da) NVIAGVKEPIRAYG$^{\mathbf{D}}$**C**SAN$^{\mathbf{D}}$**DA***	1023.5073	1023.5074	-0.14
y19	VIAGVKEPIRAYG^{D}CSAN^{D}DA	967.4936	967.4930	0.61
y19*	(-2 Da) VIAGVKEPIRAYG$^{\mathbf{D}}$**C**SAN$^{\mathbf{D}}$**DA***	966.4858	966.4837	2.17
y18	IAGVKEPIRAYG^{D}CSAN^{D}DA	917.9594	917.9584	1.10
y18*	(-2 Da) IAGVKEPIRAYG$^{\mathbf{D}}$**C**SAN$^{\mathbf{D}}$**DA***	916.9516	916.9531	-1.65
y17	AGVKEPIRAYG^{D}CSAN^{D}DA	861.4174	861.4179	-0.57
y17*	(-2 Da) AGVKEPIRAYG$^{\mathbf{D}}$**C**SAN$^{\mathbf{D}}$**DA***	860.4096	860.4082	1.59
y16	GVKEPIRAYG^{D}CSAN^{D}DA	1650.7904	1650.7911	-0.41
y16*	(-2 Da) GVKEPIRAYG$^{\mathbf{D}}$**C**SAN$^{\mathbf{D}}$**DA***	1648.7748	1648.7810	-3.77
y15	VKEPIRAYG^{D}CSAN^{D}DA	1593.7690	1593.7753	-3.98
y15*	(-2 Da) VKEPIRAYG$^{\mathbf{D}}$**C**SAN$^{\mathbf{D}}$**DA***	1591.7533	1591.7480	3.33
y14	KEPIRAYG^{D}CSAN^{D}DA	1494.7006	1494.7070	-4.28
y14*	(-2 Da) KEPIRAYG$^{\mathbf{D}}$**C**SAN$^{\mathbf{D}}$**DA***	1492.6849	1492.6878	-1.94
y13	EPIRAYG^{D}CSAN^{D}DA	1366.6056	1366.6066	-0.73
y13*	(-2 Da) EPIRAYG$^{\mathbf{D}}$**C**SAN$^{\mathbf{D}}$**DA***	1364.5899	1364.5901	-0.17
y12	PIRAYG^{D}CSAN^{D}DA	1237.5630	1237.5603	2.20
y12*	(-2 Da) PIRAYG$^{\mathbf{D}}$**C**SAN$^{\mathbf{D}}$**DA***	1235.5473	1235.5443	2.41
y11	IRAYG^{D}CSAN^{D}DA	1140.5102	1140.5092	0.86
y11*	(-2 Da) IRAYG$^{\mathbf{D}}$**C**SAN$^{\mathbf{D}}$**DA***	1138.4946	1138.4978	-2.80
y10	RAYG^{D}CSAN^{D}DA	1027.4262	1027.4281	-1.83
y10*	(-2 Da) RAYG$^{\mathbf{D}}$**C**SAN$^{\mathbf{D}}$**DA***	1025.4105	1025.4122	-1.61
y9	AYG^{D}CSAN^{D}DA	871.3251	871.3273	-2.53
y9*	(-2 Da) AYG$^{\mathbf{D}}$**C**SAN$^{\mathbf{D}}$**DA***	869.3094	869.3088	0.69
y8	YG^{D}CSAN^{D}DA	800.2879	800.2876	0.37
y8*	(-2 Da) YG$^{\mathbf{D}}$**C**SAN$^{\mathbf{D}}$**DA***	798.2723	798.2700	2.91
y7	G^{D}CSAN^{D}DA	637.2246	637.2258	-1.83
y7*	(-2 Da) G$^{\mathbf{D}}$**C**SAN$^{\mathbf{D}}$**DA***	635.2090	635.2107	-2.61
y6	^{D}CSAN^{D}DA	580.2032	580.2040	-1.32
y6*	(-2 Da) $^{\mathbf{D}}$**C**SAN$^{\mathbf{D}}$**DA***	578.1875	578.1859	2.82
y5	SAN^{D}DA	477.1940	477.1938	0.34
y5*	(-1 Da) SAN$^{\mathbf{D}}$**DA***	476.1861	Not Found	N/A
y4	AN^{D}DA	390.1619	390.1617	0.43
y4*	(-1 Da) AN$^{\mathbf{D}}$**DA***	389.1541	Not Found	N/A
y3	N^{D}DA	319.1248	319.1253	-1.41
y3*	(-1 Da) N$^{\mathbf{D}}$**DA***	318.1170	318.1170	0.14
y2	^{D}DA	205.0819	205.0816	1.43
y2*	(-1 Da) $^{\mathbf{D}}$**DA***	204.0741	Not Found	N/A

Figure S36. Iodoacetic Acid Treatment of unmodified and modified Leader-^{D}CSANDA peptides
Leader = LKQINVIAGVKEPIRAYG

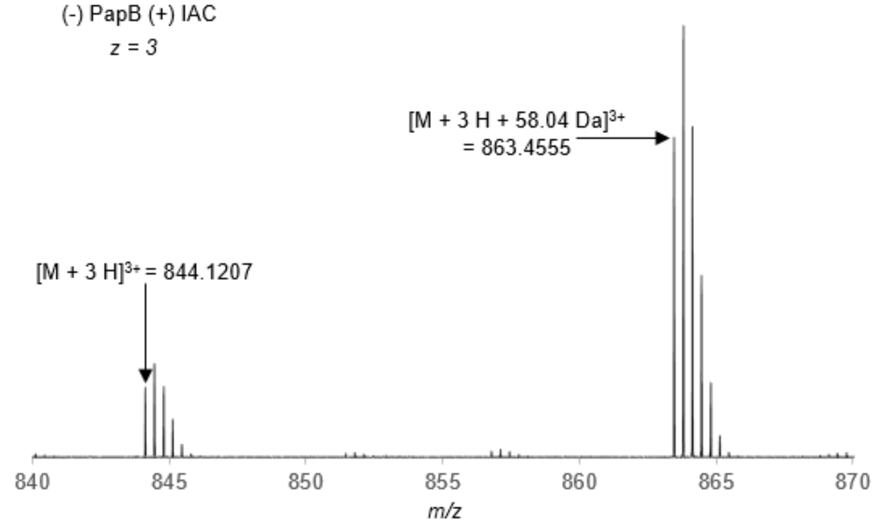

Sequence	Expected Monoisotopic Mass	Observed Monoisotopic Mass	Ppm Error
Leader-^{D}CSANDA	(-) PapB: 844.1197	844.1207	1.18
Leader-^{D}CSANDA + S-carboxymethyl	(-) PapB + (58.04 Da): 863.4549	863.4555	0.69

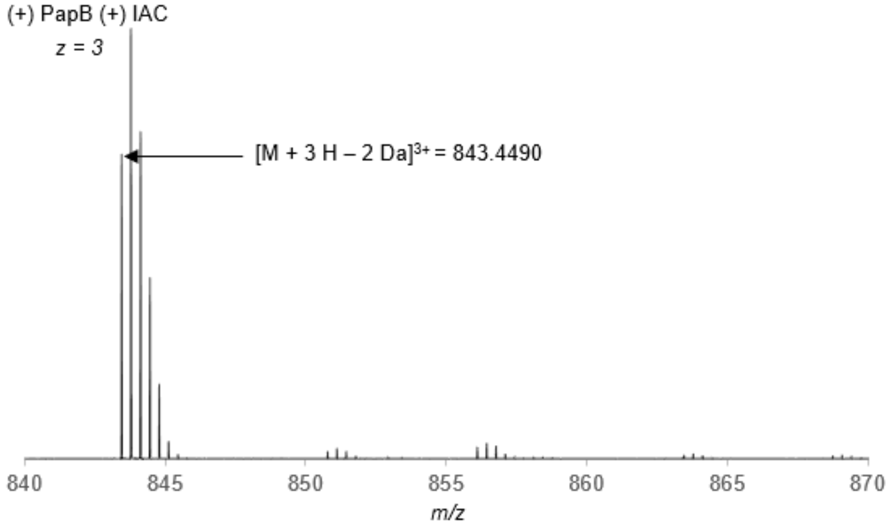

Sequence	Expected Monoisotopic Mass	Observed Monoisotopic Mass	Ppm Error
Leader-^{D}CSANDA	(+) PapB - (2 Da): 843.4478	843.4490	1.42
Leader-^{D}CSANDA + S-carboxymethyl	(-) PapB + (58.04 Da): 863.4549	Not Found	N/A

Figure S37. Iodoacetic Acid Treatment of unmodified and modified Leader-CSAND^{D}A peptides
Leader = LKQINVIAGVKEPIRAYG

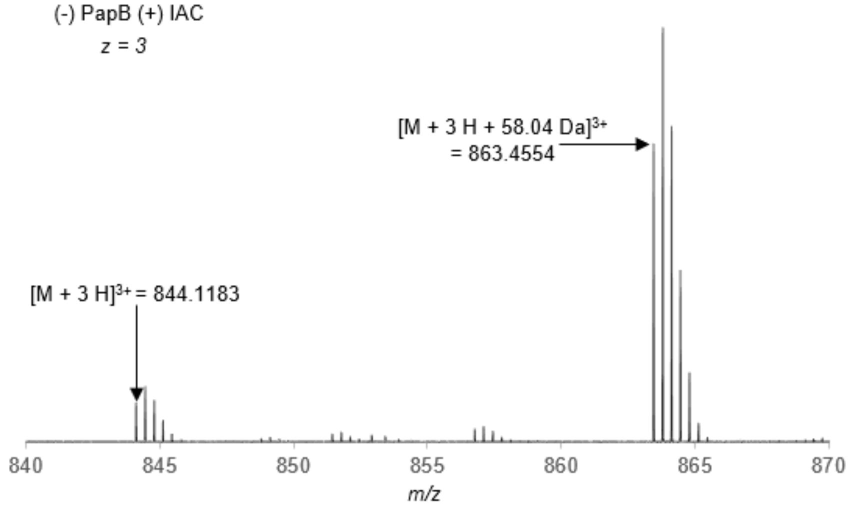

Sequence	Expected Monoisotopic Mass	Observed Monoisotopic Mass	Ppm Error
Leader-CSAN^{D}DA	(-) PapB: 844.1197	844.1183	-1.66
Leader-CSAN^{D}DA + S-carboxymethyl	(-) PapB + (58.04 Da): 863.4549	863.4554	0.58

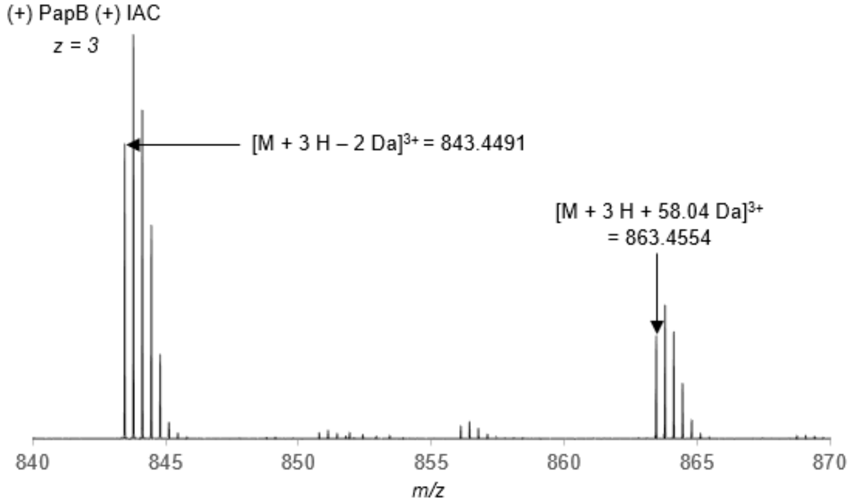

Sequence	Expected Monoisotopic Mass	Observed Monoisotopic Mass	Ppm Error
Leader-CSAN^{D}DA	(+) PapB - (2 Da): 843.4478	843.4491	1.54
Leader-CSAN^{D}DA + S-carboxymethyl	(-) PapB + (58.04 Da): 863.4549	863.4553	0.46

Figure S38. Iodoacetic Acid Treatment of unmodified and modified Leader-^{D}CSAND^{D}A peptide
Leader = LKQINVIAGVKEPIRAYG

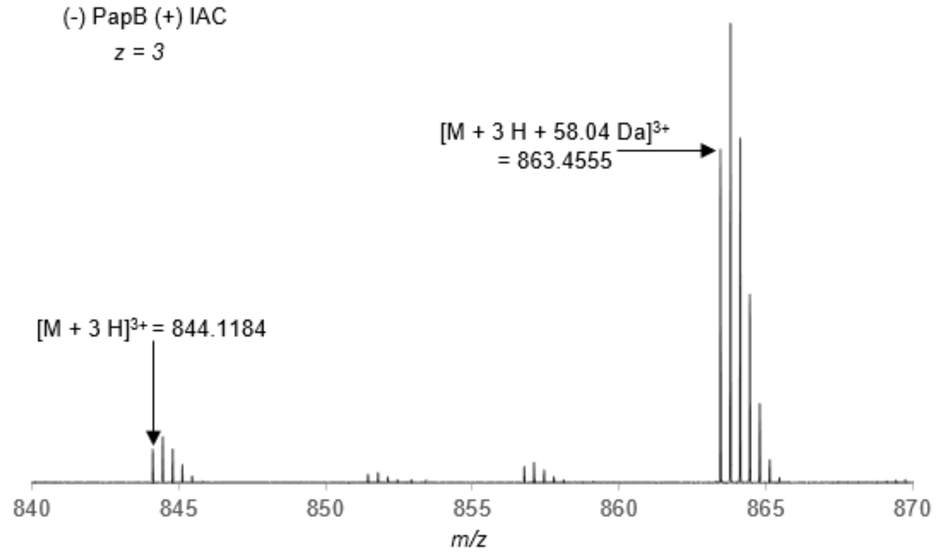

Sequence	Expected Monoisotopic Mass	Observed Monoisotopic Mass	Ppm Error
Leader-^{D}CSAN^{D}DA	(-) PapB: 844.1197	844.1184	-1.54
Leader-^{D}CSAN^{D}DA + S-carboxymethyl	(-) PapB + (58.04 Da): 863.4549	863.4555	0.69

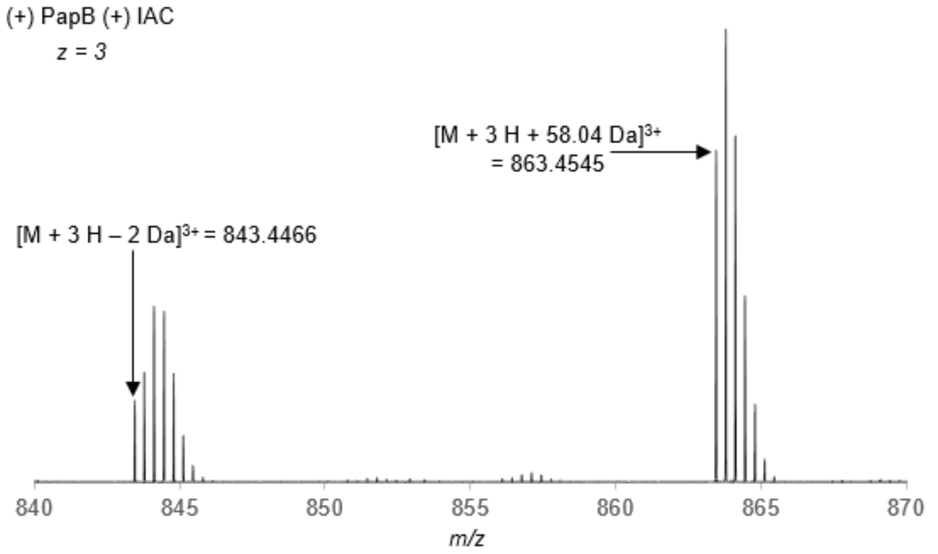

Sequence	Expected Monoisotopic Mass	Observed Monoisotopic Mass	Ppm Error
Leader-^{D}CSAN^{D}DA	(+) PapB - (2 Da): 843.4478	843.4466	-1.42
Leader-^{D}CSAN^{D}DA + S-carboxymethyl	(-) PapB + (58.04 Da): 863.4549	863.4545	-0.46

Figure S39. Mass Spectra of msPapA peptides with interchanged D and C residues

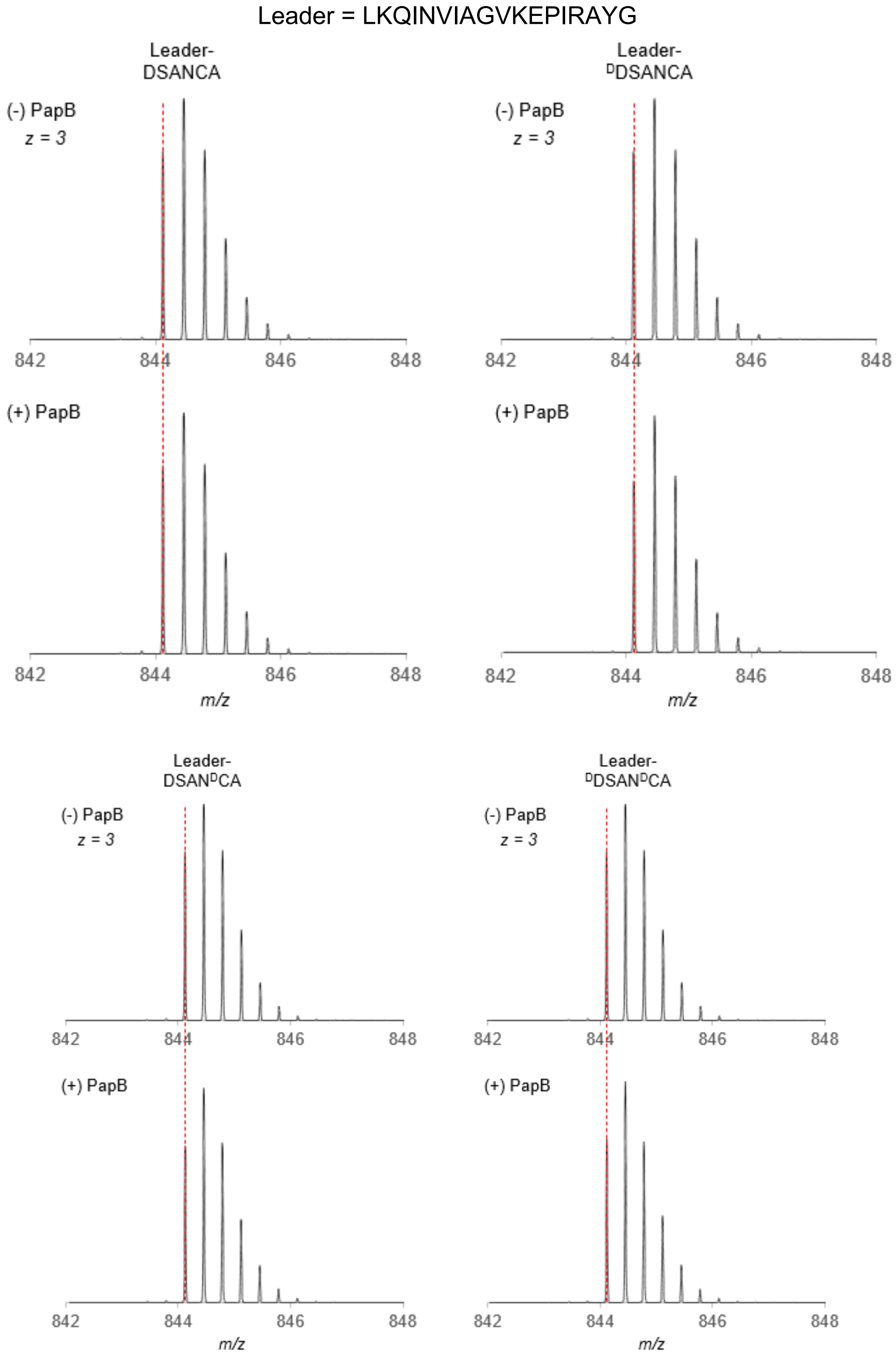

Table S40. Expected and observed monoisotopic masses for octreotide analogs

Sequence	Expected Monoisotopic Mass	Observed Monoisotopic Mass	Ppm Error
Leader-FCFAKTETA (z = 3)	(-) PapB: 989.8757<br>(+) PapB (-2Da): 989.2038	989.8746<br>989.2042	-1.111<br>0.404
Leader-ENLYFQ ^{D}F**C**F^{D}WKT**E**T (z = 3)	(-) PapB: 1250.3235<br>(+) PapB (-2Da): 1249.6516	1250.3226<br>1249.6507	-0.720<br>-0.720
^{D}F**C**F^{D}WKT**E**T (z = 2)	(-) PapB: 531.2417<br>(+) PapB (-2Da): 530.2339	531.2427<br>530.2354	1.882<br>2.829

Figure S41. Tandem Mass Spectrometry analysis of the octreotide analog Leader-FCFAKTETA

Sequence: LKQINVIAGVKEPIRAYGF**C**FAKT**E**TA

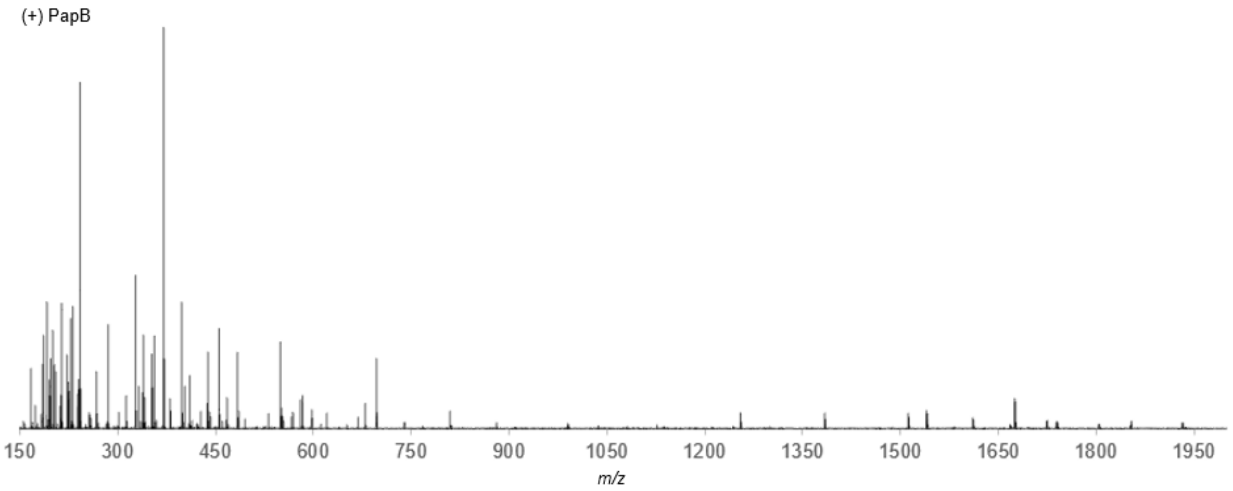

Unhighlighted indicates *z* = 1 charge state.
Gold highlight indicates *z* = 2 charge state.
Teal highlight indicates *z* = 3 charge state.
* Indicates the fragment originated from the modified peptide.

Ion	Sequence	Expected Monoisotopic Mass	Observed Monoisotopic Mass	Ppm Error
b2*	LK*	242.1863	242.1862	0.40
b3*	LKQ*	370.2449	370.2444	1.26
b4*	LKQI*	483.3289	483.3289	0.07
b5*	LKQIN*	597.3719	597.3708	1.93
b6*	LKQINV*	696.4403	696.4398	0.73
b7*	LKQINVI*	809.5244	809.5219	3.08
b8*	LKQINVIA*	880.5615	880.5630	-1.70
b9*	LKQINVIAG*	937.5829	937.5856	-2.89

b10*	LKQINVIAGV*	1036.6513	1036.6533	-1.89
b11*	LKQINVIAGVK*	1164.7463	1164.7471	-0.69
b12*	LKQINVIAGVKE*	1293.7889	1293.7898	-0.68
b13*	LKQINVIAGVKEP*	1390.8417	1390.8393	1.74
b14*	LKQINVIAGVKEPI*	1503.9257	1503.9232	1.63
b15*	LKQINVIAGVKEPIR*	1660.0268	1660.0302	-2.05
b16*	LKQINVIAGVKEPIRA*	866.0356	866.0376	-2.31
b17*	LKQINVIAGVKEPIRAY*	947.5673	Not Found	N/A
b18*	LKQINVIAGVKEPIRAYG*	976.0780	976.0802	-2.29
b19*	LKQINVIAGVKEPIRAYGF*	1049.6122	Not Found	N/A
b20*	LKQINVIAGVKEPIRAYGF**C***	1100.6129	Not Found	N/A
b21*	LKQINVIAGVKEPIRAYGF**C**F*	783.1005	Not Found	N/A
b22*	LKQINVIAGVKEPIRAYGF**C**FA*	806.7795	Not Found	N/A
b23*	LKQINVIAGVKEPIRAYGF**C**FAK*	849.4779	Not Found	N/A
b24*	LKQINVIAGVKEPIRAYGF**C**FAKT*	883.1604	Not Found	N/A
b25*	LKQINVIAGVKEPIRAYGF**C**FAKT**E***	925.8387	925.8365	2.38
b26*	LKQINVIAGVKEPIRAYGF**C**FAKT**E**T*	959.5212	959.5200	1.28
[M]*	LKQINVIAGVKEPIRAYGF**C**FAKT**E**TA*	989.2038	989.2037	0.06
y26*	KQINVIAGVKEPIRAYGF**C**FAKT**E**TA*	951.5091	951.5088	0.36
y25*	QINVIAGVKEPIRAYGF**C**FAKT**E**TA*	908.8108	908.8126	-2.02
y24*	INVIAGVKEPIRAYGF**C**FAKT**E**TA*	866.1246	866.1222	2.77
y23*	NVIAGVKEPIRAYGF**C**FAKT**E**TA*	828.4299	828.4302	-0.40
y22*	VIAGVKEPIRAYGF**C**FAKT**E**TA*	790.4156	790.4152	0.46
y21*	IAGVKEPIRAYGF**C**FAKT**E**TA*	757.3928	757.3936	-1.00
y20*	AGVKEPIRAYGF**C**FAKT**E**TA*	1079.0435	1079.0453	-1.65
y19*	GVKEPIRAYGF**C**FAKT**E**TA*	1043.5249	Not Found	N/A
y18*	VKEPIRAYGF**C**FAKT**E**TA*	1015.0142	1015.0142	0.00
y17*	KEPIRAYGF**C**FAKT**E**TA*	965.4800	965.4811	-1.18
y16*	EPIRAYGF**C**FAKT**E**TA*	901.4325	901.4332	-0.77
y15*	PIRAYGF**C**FAKT**E**TA*	1672.8152	1672.8202	-2.99
y14*	IRAYGF**C**FAKT**E**TA*	1575.7624	1575.7599	1.58
y13*	RAYGF**C**FAKT**E**TA*	1462.6784	1462.6848	-4.38
y12*	AYGF**C**FAKT**E**TA*	1306.5773	1306.5724	3.78
y11*	YGF**C**FAKT**E**TA*	1235.5401	1235.5352	3.93
y10*	GF**C**FAKT**E**TA*	1072.4768	1072.4783	-1.44
y9*	F**C**FAKT**E**TA*	1015.4553	1015.4590	-3.68
y8*	**C**FAKT**E**TA*	868.3869	868.3869	0
y7*	FAKT**E**TA*	766.3856	Not Found	N/A
y6*	AKT**E**TA*	619.3172	Not Found	N/A
y5*	KT**E**TA*	548.2800	Not Found	N/A
y4*	T**E**TA*	420.1851	Not Found	N/A
y3*	**E**TA*	319.1374	Not Found	N/A
y2*	TA*	191.1026	191.1023	1.53

Figure S42. Tandem Mass Spectrometric analysis of modified Leader-TEV-OctreotideC7E peptide peptide

Sequence: LKQINVIAGVKEPIRAYGENLYFQ^{D}F**C**F^{D}WKT**E**T

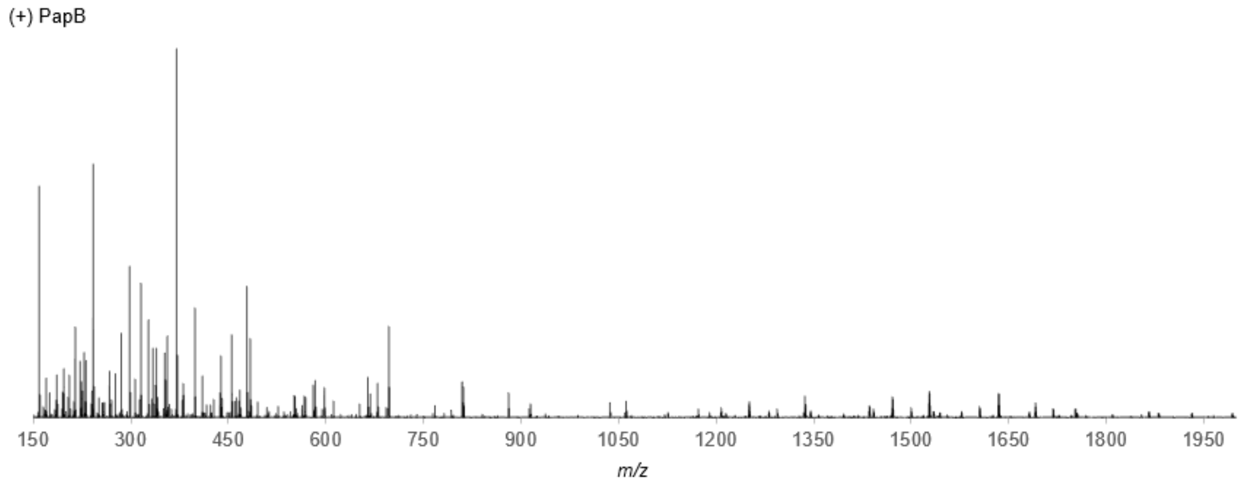

Gold highlight indicates z = 2 charge state.
Teal highlight indicates z = 3 charge state.
* Indicates the fragment originated from the modified peptide.

Ion	Sequence	Expected Monoisotopic Mass	Observed Monoisotopic Mass	Ppm Error
b2*	LK*	242.1863	242.1863	0.16
b3*	LKQ*	370.2449	370.2442	1.82
b4*	LKQI*	483.3289	483.3283	1.24
b5*	LKQIN*	597.3719	597.3732	-2.16
b6*	LKQINV*	696.4403	696.4383	2.86
b7*	LKQINVI*	809.5244	809.5253	-1.08
b8*	LKQINVIA*	880.5615	880.5605	1.16
b9*	LKQINVIAG*	937.5829	937.5827	0.17
b10*	LKQINVIAGV*	1036.6513	1036.6497	1.51
b11*	LKQINVIAGVK*	1164.7463	1164.7426	3.14
b12*	LKQINVIAGVKE*	1293.7889	1293.7843	3.56
b13*	LKQINVIAGVKEP*	1390.8417	Not Found	N/A
b14*	LKQINVIAGVKEPI*	1503.9257	1503.9298	-2.71
b15*	LKQINVIAGVKEPIR*	830.5171	830.5180	-1.14
b16*	LKQINVIAGVKEPIRA*	866.0356	866.0352	0.49
b17*	LKQINVIAGVKEPIRAY*	947.5673	947.5655	1.94
b18*	LKQINVIAGVKEPIRAYE*	1012.0886	1012.0883	0.25
b19*	LKQINVIAGVKEPIRAYEN*	1069.1100	1069.1100	0.03
b20*	LKQINVIAGVKEPIRAYENL*	1125.6521	1125.6530	-0.79
b21*	LKQINVIAGVKEPIRAYENLY*	1207.1837	1207.1862	-2.06

b22*	LKQINVIAGVKEPIRAYENLYF*	854.1477	854.1463	1.61
b23*	LKQINVIAGVKEPIRAYENLYFQ*	896.8339	896.8345	-0.69
b24*	LKQINVIAGVKEPIRAYENLYFQF*	945.8567	945.8596	-3.06
b25*	LKQINVIAGVKEPIRAYENLYFQF**C***	979.8572	Not Found	N/A
b26*	LKQINVIAGVKEPIRAYENLYFQF**C**F*	1028.8800	Not Found	N/A
b27*	LKQINVIAGVKEPIRAYENLYFQF**C**FW*	1090.9064	Not Found	N/A
b28*	LKQINVIAGVKEPIRAYENLYFQF**C**FWK*	1133.6047	Not Found	N/A
b29*	LKQINVIAGVKEPIRAYENLYFQF**C**FWKT*	1167.2873	Not Found	N/A
b30*	LKQINVIAGVKEPIRAYENLYFQF**C**FWKT**E***	1209.9655	1209.9655	0.01
[M]*	LKQINVIAGVKEPIRAYENLYFQF**C**FWKT**E**T*	1249.6516	1249.6538	-1.75
y30*	KQINVIAGVKEPIRAYENLYFQF**C**FWKT**E**T*	1211.9569	1211.9537	2.62
y29*	QINVIAGVKEPIRAYENLYFQF**C**FWKT**E**T*	1169.2586	1169.2607	-1.82
y28*	INVIAGVKEPIRAYENLYFQF**C**FWKT**E**T*	1126.5724	1126.5758	-3.06
y27*	NVIAGVKEPIRAYENLYFQF**C**FWKT**E**T*	1088.8777	1088.8819	-3.86
y26*	VIAGVKEPIRAYENLYFQF**C**FWKT**E**T*	1050.8634	1050.8645	-1.01
y25*	IAGVKEPIRAYENLYFQF**C**FWKT**E**T*	1017.8406	1017.8382	2.39
y24*	AGVKEPIRAYENLYFQF**C**FWKT**E**T*	980.1459	Not Found	N/A
y23*	GVKEPIRAYENLYFQF**C**FWKT**E**T*	956.4669	956.4659	1.06
y22*	VKEPIRAYENLYFQF**C**FWKT**E**T*	1405.6860	1405.6838	1.54
y21*	KEPIRAYENLYFQF**C**FWKT**E**T*	1356.1518	1356.1510	0.58
y20*	EPIRAYENLYFQF**C**FWKT**E**T*	1292.1043	1292.1088	-3.51
y19*	PIRAYENLYFQF**C**FWKT**E**T*	1227.5830	1227.5785	3.64
y18*	IRAYENLYFQF**C**FWKT**E**T*	1179.0566	1179.0574	-0.64
y17*	RAYENLYFQF**C**FWKT**E**T*	1122.5146	1122.5166	-1.81
y16*	AYENLYFQF**C**FWKT**E**T*	1044.4640	1044.4647	-0.66
y15*	YENLYFQF**C**FWKT**E**T*	1008.9455	1008.9429	2.59
y14*	ENLYFQF**C**FWKT**E**T*	927.4138	Not Found	N/A
y13*	NLYFQF**C**FWKT**E**T*	862.8925	Not Found	N/A
y12*	LYFQF**C**FWKT**E**T*	1610.7348	1610.7303	2.81
y11*	YFQF**C**FWKT**E**T*	1497.6507	1497.6439	4.52
y10*	FQF**C**FWKT**E**T*	1334.5874	1334.5889	-1.11
y9*	QF**C**FWKT**E**T*	1187.5190	1187.5209	-1.61
y8*	F**C**FWKT**E**T*	1059.4604	1059.4621	-1.64
y7*	**C**FWKT**E**T*	912.3920	912.3932	-1.29
y6*	FWKT**E**T*	810.3907	Not Found	N/A
y5*	WKT**E**T*	663.3222	Not Found	N/A
y4*	KT**E**T*	477.2429	Not Found	N/A
y3*	T**E**T*	349.1480	Not Found	N/A
y2*	**E**T*	248.1003	Not Found	N/A

References

(1) Precord, T. W.; Mahanata, N.; Mitchell, D. A. Reconstitution and Substrate Specificity of the Thioether-Forming Radical S-Adenosylmethionine Enzyme in Freyrasin Biosynthesis. ACS Chem. Biol. 2019, 14 (9), 1981-89.

(2) Outten, F.W.; Djaman, O.; Storz, G. A suf operon requirement for Fe–S cluster assembly during iron starvation in Escherichia coli. Mol. Microbio. 2004, 52, 861-872.

(3) Wollers, S.; Layer, G.; Garcia-Serres, R.; Signor, L.; Clemancey, M.; Latour, J. M.; Fontecave, M.; Ollagnier de Choudens, S. Iron-sulfur (Fe-S) cluster assembly: the SufBCD complex is a new type of Fe-S scaffold with a flavin redox cofactor. J. Biol. Chem., 2010, 285 (30), 23331–23341.

(4) Bruender, N. A.; and Bandarian, V. SkfB Abstracts a Hydrogen Atom from Cα on SkfA to Initiate Thioether Cross-Link Formation. Biochemistry, 2016, 55, 4131–4134.

(5) Young, A. P.; Bandarian, V. TYW1: A Radical SAM Enzyme Involved in the Biosynthesis of Wybutosine Bases. Methods Enzymol. 2018, 606, 119-153.
