# Supplementary material for: Leveraging Substrate Promiscuity of a Radical S-Adenosyl-L-methionine RiPP Maturase toward Intramolecular Peptide Cross-Linking Applications
Source: ACS Cent Sci. 2022 Aug 1;8(8):1209–17. doi: 10.1021/acscentsci.2c00501 (PMC9413430; doi:10.1021/acscentsci.2c00501)
Supplement: Supplementary file 2 — oc2c00501_si_002.pdf [file oc2c00501_si_002.pdf]

oc-2022-00501h.R1

Name: Peer Review Information for "Leveraging substrate promiscuity of a radical SAM RiPP maturase towards intramolecular peptide crosslinking applications"

First Round of Reviewer Comments

Reviewer: 1

Comments to the Author

The authors describe in this manuscript the use of the radical SAM (rSAM) protein PapB for generation of macrocycles in peptides. PapB crosslinks the sulfur of Cys with the carbon adjacent to the side chain carboxylates of Asp and Glu. This study provides data illustrating the substantial tolerance of the enzyme towards changes in the sequence of the substrate, including spacings between a leader peptide and the Cys-Asp/Glu pair to be crosslinked, and spacings and sequence between the Cys and Asp/Glu.

The authors show that many ring sizes can be accessed and that even the presence of D-amino acids is tolerated, but that preparation of rings larger than 6 amino acids or composed of just 2 amino acids is not feasible. This flexibility rules out that the enzyme introduces the crosslinks based on the side chains of the Cys and Asp/Glu being on one side of an alpha helix as had been proposed, and suggests the enzyme recognizes specifically the Cys and Asp side chains irrespective of their location in a peptide (provided they are spaced by 1-5 amino acids). They use this knowledge to make a series of ring patterns including non-overlapping and nested rings. They also show that a single ring installed in a heterologous host in a recent application of PapB is not what the enzyme does in vitro.

Finally, the authors illustrate the flexibility of the PapB enzyme by synthesizing an analog of the drug octreotide, a peptide containing to D-amino acids that is cyclized by a disulfide linkage. PapB tolerated the D-amino acids, and also of note, the TEV protease cleaved next to one of them (D-Phe) to liberate the cyclic peptide from the leader peptide.

Given the high contemporary interest in cyclic peptides as a new drug modality that can recognize protein surfaces and the associated interest in new methods of accessing these structures in high yields and with high substrate scope, this study is very well suited for ACS Cent Sci. It combines rigor in chemical analysis of enzymatic products with recognition of synthetic utility of biological catalysts. This work provides a thorough evaluation of what can and what cannot be done with PapB and will be of great value to the community for the design of substrates to access cyclic peptide libraries. Since ACS Cent Sci has a broad readership, the authors can improve the accessibility of the paper to non-specialists. Some suggestions are listed below.

The concept of a leader peptide is not really explained and the word leader first appears in the legend of Fig 2 without ever having been explained. A short discussion of leader peptides in RiPP biosynthesis would be important for non-specialists, and I suggest that in the legend of Fig 1 the authors indicate that the blue sequence in panel D is the leader peptide (I also suggest to make the font of that panel smaller

as it seems unnecessarily large). I would also suggest adding the ring pattern of freyrasin to Figure 1 to help the reader understand several sentences in the paper (e.g. page 4, left, lines 33-36 and page 5, right, lines 45-48). The authors can box certain patterns to indicate what they mean by in-line and nested.

Similarly, I believe that it would benefit the reader if the authors show the structure of octreotide in Figure 5.

Figure 3 is confusing. In Figure 2 the authors nicely line up the peptide under investigation, with the MS data on enzyme turnover, and the tandem MS on that peptide to illustrate the crosslink. But in Fig 3 that correlation is missing, with the peptides at the top in panel A not correlating with the MS data in panel B and the tandem MS fragments in panel C. Please make the peptides in panel A in line with those in panels B and C, or if the point is to illustrate the ring patterns, arrange them such that they do not appear to correspond to the data in panels B and C.

Specific comments (paragon numbering):

Page 1, line 41 (left). When citing ref 5, the authors should indicate that PTM is challenging using chemical methods (which is what the citation discusses). By using the word PTM most readers will assume the authors are discussing enzymatic methods which are not the topic of this cited work (which focuses on chemical reactions on peptides).

The net reaction is a two-electron oxidation. Should Fig 2A therefore show 2 electrons?

Page 3, line 29-31. "As with above". Please reword.

Several occasions (incl p. 4, line 41): "within <" This wording is redundant.

Page 4 line 42-43. Mass spectroscopy should be mass spectrometry

Page 4 lines 48-53. "The results with ……processed peptide." The authors twice se the term recognition sequence, but I think they mean two different things (the leader peptide in one instance and the CXnD motif in the other). Pleas reword to make this more clear.

Page 4, right. "in from"

Figure 4 legend "Fig. SX"

Page 5. Perhaps change the wording "colocalized in an alpha helix" to "located on the same face of an alpha helix"

Page 5 right. change "of a conserved Arg residue" to "of a conserved Arg residue on PapB". (because the entire paragraph has been about residues on PapA).

Fig. 5. Legend. A sentence appears incomplete: "After modification by PapB."

Page 6, right. remains should be remain

Page 7, left. "with one of the auxiliary clusters". This is the first time this is introduced and although it will be obvious to rSAM experts, it will not be to the general reader. Changing to "with one of the

auxiliary FeS clusters" would already help. Adding a reference where auxiliary clusters are discussed would also be beneficial.

Page 7. Write out vDW

Abbreviations. The list contains several abbreviations that are not used in the main text (and some typos).

References:

ref 6 shows first names instead of last names (also formatting issues in the refs).

Reviewer: 2

Comments to the Author

This is a very thorough dissection of the substrate promiscuity of the radical SAM ranthionine synthase, PapB by Eastman et al. Follows on from the 2019 report from Precord et al., which first characterized the enzyme (ref. 24), and subsequent work by King et al. (ref. 29), which used the enzyme in an in vivo evolution system. The results are highly complimentary to those two studies and serve to answer some important open questions. The authors do a basic kinetic characterization of the enzyme and validation of activity before submitting to modification of a number of mutant versions of a minimized substrate. The main focus of the substrate promiscuity analysis is on changes in size and sequence diversity between the crosslinking positions (Cys and typically Asp or Glu). The enzyme shows a remarkable dedication to cross linking at these charged, Asp/Glu side chains, seemingly despite the distance from the partner Cys residue. Importantly, the authors show that this selectivity seems independent of the secondary structure of the interstitial peptide, through studies that incorporate D-amino acids and other features that would counteract or break up any innate helicity. These data are informative, as they set the ground for the application of this enzyme to generating a non-reducible disulfide isostere in an analog of the FDA-approved octreotide. The paper tracks well with the field of RiPPs enzymology, which, while still concerned with the elucidation of new peptide modifying enzymes has begun to turn on their application in synthetic and drug discovery-type efforts. Overall, I feel that this strong work that is worthy of consideration for publication in ACS Central Science, but I have two concerns that I recommend be addressed before publication.

1) Fuller characterization of the ranthipeptide linkages (major): Data from the original PapB report suggests that, at least in two instances, the position of thioether bond formation (beta or gamma to the main chain carbonyl) is dictated by interaction of the carboxylate on the crosslink (D or E) partner with an active site Arg. In that paper, the specific position was characterized by detailed NMR of the peptide, which would not be possible on the number of substrates in the current (Eastman et al.) manuscript, yet there is still the question of whether the thioether is always installed alpha to the side chain carboxylate. These RS domains are used in a number of RiPP enzymes, including ranthi and sactipeptides, as well as epimerases, and a number of other reaction processes, where the BDEs for abstraction are close enough to suggest that positioning relative the radical center is as important as the redox potential of the given SAM-enzyme intermediate radical. Therefore, it is possible, even likely that substantially modifying the substrate may move the crosslink reaction from alpha to beta or even further along on the chain. The

data in the current manuscript suggests that the enzyme is consistent because the linkage is always to the D or E, but there is no deeper analysis of the MS/MS data to confirm that it is always at the same relative carbon. Mitchell et al. used MS/MS to first demonstrate that the ranthis were not sactis, is there a similarly distinctive MS/MS pattern that could be used here to confirm the site of cross link on the acidic acceptor residue?

2) Novelty (minor): Although the peptide sequences investigated as RiPP RS substrates here are novel and represent sincere departures from the level of alteration that has been previously reported, it should be noted that the level of promiscuity is far from unprecedented. In addition to the King et al work, which the authors sited there are several examples of extreme promiscuity in this family that suggests that also somewhat diminish the novelty (and potential impact) of the current work: Himes et al., 2016 (doi.org/10.1021/acschembio.6b00042), Burkhart et al., 2017 (doi.org/10.1021/acscentsci.7b00141), Vagstad et al., 2019 (https://doi.org/10.1002/anie.201809508), and Korneli et al., 2021, (doi.org/10.1021/acssynbio.0c00470). This is a minor concern, but the authors might consider siting some of this work to better contextualize their own.

Author's Response to Peer Review Comments:

Please see attached file.

Dear Editor:

We are grateful for the thoughtful reviews that we received from the reviewers and for the opportunity to submit this revised manuscript. It was quite gratifying to see that the reviewers assessed the work to be significant and of importance to the readership of ACS Central Science. We have carefully addressed each of their concerns as detailed in response below. In addition, we include responses to the editorial changes that were requested. Along with the revised copy of the manuscript, we are also submitting a version where the changes are

We look forward to hearing your decision on this manuscript.

Sincerely,

Vahe Bandarian

Editorial change requests:

AU EMAIL: Please include the email address of the corresponding author on the first page of the manuscript, and the Supporting Information if submitted, with an asterisk next to their name in the author list. Please be sure to label "email."

The revised manuscript includes corresponding author e-mail.

SYNOPSIS MISSING: The synopsis should be no more than 200 characters (including spaces) and should reasonably correlate with the TOC graphic. The synopsis is intended to explain the importance of the article to a broader readership across the sciences. Please place your synopsis in the manuscript file after the TOC graphic.

A 189-character synopsis has been added to the manuscript.

TOC needs a label

Done.

SI PG#S: The supporting information pages must be numbered consecutively, starting with page S1.

Done.

SI figures are not in order

This has been fixed.

Reviewer(s)' Comments to Author:

Reviewer: 1

The authors describe in this manuscript the use of the … Some suggestions are listed below.

The concept of a leader peptide is not really explained and the word leader first appears in the legend of Fig 2 without ever having been explained. A short discussion of leader peptides in RiPP biosynthesis would be important for non-specialists, and I suggest that in the legend of Fig 1 the authors indicate that the blue sequence in panel D is the leader peptide (I also suggest to make the font of that panel smaller as it seems unnecessarily large). I would also suggest adding the ring pattern of freyrasin to Figure 1 to help the reader understand several sentences in the paper (e.g. page 4, left, lines 33-36 and page 5, right, lines 45-48). The authors can box certain patterns to indicate what they mean by in-line and nested.

We thank the reviewer for these suggestions. We have modified Figure 1 as the reviewer proposed to show the freyrasin ring structure and reduced the font size. The distinction between in-line and nested crosslinks is shown schematically in a revised Figure 3.

Similarly, I believe that it would benefit the reader if the authors show the structure of octreotide in Figure 5.

The structure of octreotide is shown in the updated Figure 5.

Figure 3 is confusing. In Figure 2 the authors nicely line up the peptide under investigation, with the MS data on enzyme turnover, and the tandem MS on that peptide to illustrate the crosslink. But in Fig 3 that correlation is missing, with the peptides at the top in panel A not correlating with the MS data in panel B and the tandem MS fragments in panel C. Please make the peptides in panel A in line with those in panels B and C, or if the point is to illustrate the ring patterns, arrange them such that they do not appear to correspond to the data in panels B and C.

We thank the reviewer for this suggestion. We have made the changes that are suggested and the new figure is superior to the one that was in the original manuscript. In addition, we have now included a graphical representation of the nested and in-line crosslinks in the figure.

Specific comments (paragon numbering):

Page 1, line 41 (left). When citing ref 5, the authors should indicate that PTM is challenging using chemical methods (which is what the citation discusses). By using the word PTM most readers will assume the authors are discussing enzymatic methods which are not the topic of this cited work (which focuses on chemical reactions on peptides).

This has been addressed as suggested. The sentence now reads: "Posttranslational modification of amino acid residues is generally challenging using traditional synthetic chemistry methods[5]."

The net reaction is a two-electron oxidation. Should Fig 2A therefore show 2 electrons?

As the reviewer would appreciate, if we were to show the actual stoichiometry explicitly it would unnecessarily complicate the schematic representation. However, we replaced the "e^-" which was in the original figure with "+Reductant" so as to be consistent with the requirements for reaction, which also include SAM and PapB (both shown explicitly above the arrow).

Page 3, line 29-31. "As with above". Please reword.

Done. The phrase was removed.

Several occasions (incl p. 4, line 41): "within <" This wording is redundant.

We have removed the "within" and reworded as needed. Where errors are mentioned, we generally use the symbol "<".

Page 4 line 42-43. Mass spectroscopy should be mass spectrometry

Done.

Page 4 lines 48-53. "The results with ……processed peptide." The authors twice use the term recognition sequence, but I think they mean two different things (the leader peptide in one instance and the CXnD motif in the other). Pleas reword to make this more clear.

We have modified that section to read: "The results with expansions of the CX_nD motif in the previous section demonstrate a lack of defined specificity in the recognition sequence, beyond the preference for Cys and Asp. The data with the nested crosslinks above extend this to include distance from the leader peptide recognition sequence, as well as the individual amino acids within the processed peptide."

Page 4, right. "in from"

Fixed.

Figure 4 legend "Fig. SX"

Fixed.

Page 5. Perhaps change the wording "colocalized in an alpha helix" to "located on the same face of an alpha helix"

Done.

Page 5 right. change "of a conserved Arg residue" to "of a conserved Arg residue on PapB". (because the entire paragraph has been about residues on PapA).

The paragraph has been modified as follows: "…Previous studies have shown that mutation of a conserved Arg residue in PapB (Arg372) to an Ala abolishes activity[24]. While there are no structures of the substrate-bound enzyme, structural models suggest that this Arg residue could be near the carboxylate moiety of PapA. Inversion of the sidechain would similarly eliminate the interaction leading to no crosslinking."

Fig. 5. Legend. A sentence appears incomplete: "After modification by PapB."

Fixed.

Page 6, right. remains should be remain

Fixed.

Page 7, left. "with one of the auxiliary clusters". This is the first time this is introduced and although it will be obvious to rSAM experts, it will not be to the general reader. Changing to "with one of the auxiliary FeS clusters" would already help. Adding a reference where auxiliary clusters are discussed would also be beneficial.

We have updated that paragraph as follows to address the reviewer's comment. "…While there are no structural data, the PapB is homologous to SPASM superfamily enzymes that in addition to the RS cluster that binds and activates SAM, also house at least two additional Fe/S clusters[36-38]. It has been proposed that in the thioether crosslinking enzymes, the thiolate of the Cys can interact with one of the auxiliary Fe/S clusters[19,34-38]. …"

Page 7. Write out vDW

Done.

Abbreviations. The list contains several abbreviations that are not used in the main text (and some typos).

Updated and fixed.

References: ref 6 shows first names instead of last names (also formatting issues in the refs).

We have reviewed and updated all the references.

Reviewer: 2

This is a very thorough dissection of the substrate promiscuity … but I have two concerns that I recommend be addressed before publication.

1) Fuller characterization of the ranthipeptide linkages (major): Data from the original PapB report suggests that, at least in two instances, the position of thioether bond formation (beta or gamma to the main chain carbonyl) is dictated by interaction of the carboxylate on the crosslink (D or E) partner with an active site Arg. In that paper, the specific position was characterized by

detailed NMR of the peptide, which would not be possible on the number of substrates in the current (Eastman et al.) manuscript, yet there is still the question of whether the thioether is always installed alpha to the side chain carboxylate. These RS domains are used in a number of RiPP enzymes, including ranthi and sactipeptides, as well as epimerases, and a number of other reaction processes, where the BDEs for abstraction are close enough to suggest that positioning relative the radical center is as important as the redox potential of the given SAM-enzyme intermediate radical. Therefore, it is possible, even likely that substantially modifying the substrate may move the crosslink reaction from alpha to beta or even further along on the chain. The data in the current manuscript suggests that the enzyme is consistent because the linkage is always to the D or E, but there is no deeper analysis of the MS/MS data to confirm that it is always at the same relative carbon. Mitchell et al. used MS/MS to first demonstrate that the ranthis were not sactis, is there a similarly distinctive MS/MS pattern that could be used here to confirm the site of cross link on the acidic acceptor residue?

We thank the reviewer for this thoughtful comment. We had in fact examined all of our MS/MS data for fragments that would be expected in the case of an alpha crosslink, but had not indicated we did not see any of those in the data for the peptides shown. In the revised manuscript, we have done as the reviewer suggests and now indicate this explicitly in the text where appropriate.

2) Novelty (minor): Although the peptide sequences investigated as RiPP RS substrates here are novel and represent sincere departures from the level of alteration that has been previously reported, it should be noted that the level of promiscuity is far from unprecedented. In addition to the King et al work, which the authors sited there are several examples of extreme promiscuity in this family that suggests that also somewhat diminish the novelty (and potential impact) of the current work: Himes et al., 2016 (doi.org/10.1021/acschembio.6b00042), Burkhart et al., 2017 (doi.org/10.1021/acscentsci.7b00141), Vagstad et al., 2019 (https://doi.org/10.1002/anie.201809508), and Korneli et al., 2021, (doi.org/10.1021/acssynbio.0c00470). This is a minor concern, but the authors might consider siting some of this work to better contextualize their own.

We thank the reviewer for these suggestions and have incorporated the references into the text (references 29, 39-42).
